# The efficacy and safety of Chinese herbal medicine as an add-on therapy for type 2 diabetes mellitus patients with carotid atherosclerosis: An updated meta-analysis of 27 randomized controlled trials

**DOI:** 10.3389/fphar.2023.1091718

**Published:** 2023-03-23

**Authors:** Zehua Zhang, Yulin Leng, Zhengtao Chen, Xiaoxu Fu, Qingzhi Liang, Xi Peng, Hongyan Xie, Hong Gao, Chunguang Xie

**Affiliations:** ^1^ Hospital of Chengdu University of Traditional Chinese Medicine, Chengdu, China; ^2^ TCM Regulating Metabolic Diseases Key Laboratory of Sichuan Province, Hospital of Chengdu University of Traditional Chinese Medicine, Chengdu, China

**Keywords:** type 2 diabetes mellitus, carotid atherosclerosis, Chinese herbal medicine, Systematic review, meta-analysis

## Abstract

**Background:** Type 2 diabetes mellitus (T2DM) is a clinical metabolic syndrome characterized by persistent hyperglycemia. Patients with T2DM are more likely to have carotid atherosclerosis (CAS), which can lead to dizziness, amaurosis or even stroke. Chinese herbal medicine (CHM) has shown possible efficacy and safety in treating T2DM patients with CAS. However, the existing evidence was not robust enough and the results were out of date.

**Objective:** This meta-analysis aimed to summarize the current evidence and systematically evaluate the effects of CHM on carotid plaque, glucose and lipid metabolism and vascular endothelial parameters in T2DM patients with CAS, providing a reference for subsequent research and clinical practice.

**Methods:** This study was registered in PROSPERO as CRD42022346274. Both Chinese and English databases were searched from their inceptions to 16 July 2022. All retrieved studies were screened according to inclusion and exclusion criteria. Randomized controlled trials (RCTs) using oral CHM to treat T2DM patients with CAS were included. The literature quality was assessed using the risk of bias assessment tool in the Cochrane Handbook. Data extraction was conducted on the selected studies. Review Manager 5.4 and Stata 16.0 were used for meta-analysis. Sources of heterogeneity were explored by meta-regression or subgroup analysis. Funnel plot and Egger’s test were used to assess publication bias and the evidence quality was assessed by Grading of Recommendations Assessment, Development and Evaluation (GRADE).

**Results:** 27 eligible studies, involving 2638 patients, were included in this study. Compared with western medicine (WM) alone, the addition of CHM was significantly better in improving carotid intima-media thickness (CIMT) [mean difference (MD) = -0.11mm, 95% confidence interval (CI): −0.15 to −0.07, *p* < 0.01], carotid plaque Crouse score [MD = −1.21, 95%CI: −1.35 to −1.07, *p* < 0.01], total cholesterol (TC) [MD = −0.34 mmol/L, 95%CI: −0.54 to −0.14, *p* < 0.01], triglyceride (TG) [MD = −0.26 mmol/L, 95%CI: −0.37 to −0.15, *p* < 0.01], low-density lipoprotein cholesterol (LDL-C) [MD = −0.36 mmol/L, 95%CI: −0.47 to −0.25, *p* < 0.01], high-density lipoprotein cholesterol (HDL-C) [MD = 0.22 mmol/L, 95%CI: 0.13 to 0.30, *p* < 0.01], glycated hemoglobin (HbA1c) [MD = −0.36%, 95%CI: −0.51 to −0.21, *p* < 0.01], fasting blood glucose (FBG) [MD = −0.33 mmol/L, 95%CI: −0.50 to −0.16, *p* < 0.01], 2-h postprandial glucose (2hPG) [MD = −0.52 mmol/L, 95%CI: −0.95 to −0.09, *p* < 0.01], homeostasis model assessment of insulin resistance (HOMA-IR) [standardized mean difference (SMD) = −0.88, 95%CI: −1.36 to −0.41, *p* < 0.01] and homeostasis model assessment of beta-cell function (HOMA-β) [MD = 0.80, 95%CI: 0.51 to 1.09, *p* < 0.01]. Due to the small number of included studies, it is unclear whether CHM has an improving effect on nitric oxide (NO), endothelin-1 (ET-1), peak systolic velocity (PSV) and resistance index (RI). No serious adverse events were observed.

**Conclusion:** Based on this meta-analysis, we found that in the treatment of T2DM patients with CAS, combined with CHM may have more advantages than WM alone, which can further reduce CIMT and carotid plaque Crouse score, regulate glucose and lipid metabolism, improve insulin resistance and enhance islet β-cell function. Meanwhile, CHM is relatively safe. However, limited by the quality and heterogeneity of included studies, the efficacy and safety of CHM remain uncertain. More high-quality studies are still needed to provide more reliable evidence for the clinical application of CHM.

**Systematic Review Registration:** https://www.crd.york.ac.uk/PROSPERO/, identifier CRD42022346274

## Introduction

Type 2 diabetes mellitus (T2DM) is a chronic progressive metabolic disease characterized by persistent hyperglycemia, insulin resistance and islet β-cell dysfunction (Roden and Shulman, 2019; Galicia-Garcia et al., 2020). With changes in lifestyle and dietary structure, the prevalence of T2DM continues to increase (Zheng et al., 2018; Wang et al., 2021a). In 2021, there were about 573 million adults with diabetes worldwide, of whom about 90% had T2DM (IDF, 2021). In the process of T2DM, pathological factors such as glucose and lipid metabolism disorders, inflammatory stimulation or oxidative stress impair vascular endothelial cells, causing vascular endothelial dysfunction and subintimal lipid accumulation, eventually atherosclerosis (La Sala et al., 2019; Poznyak et al., 2020; Ye et al., 2022a). The carotid arteries are the earliest involved blood vessels in the development of atherosclerosis (Beckman et al., 2002), which initially manifests as thickening of carotid intima-media. As the disease progresses, carotid plaques gradually form, resulting in carotid stenosis or occlusion. Carotid atherosclerosis (CAS) is a significant risk factor for ischemic cerebrovascular diseases such as stroke or transient ischemic attack (Kamtchum-Tatuene et al., 2020; Bos et al., 2021). In addition, CAS indirectly reflects the overall atherosclerotic burden of large and medium arteries, and its severity is positively correlated with cardiovascular events (Nonin et al., 2017; Polak et al., 2017; Sillesen et al., 2018). More than 70% of T2DM patients suffer from CAS (Mostaza et al., 2015). Chinese Atherosclerosis Risk Evaluation study has shown that T2DM patients are more likely to have carotid plaque with calcification and lipid-rich necrotic cores than people without T2DM, suggesting that T2DM patients may develop more severe atherosclerotic disease (Gao et al., 2019). Moreover, T2DM patients have about twice the risk of stroke as those without T2DM and have a worse prognosis after stroke (Chen et al., 2016; Pikula et al., 2018). Therefore, early intervention for T2DM patients with CAS plays an important role in delaying the occurrence and development of cardiovascular and cerebrovascular events.

The current treatment for T2DM patients with CAS mainly focuses on controlling blood glucose, regulating lipids, stabilizing plaque and anti-platelet aggregation (CSN, 2017; CDS, 2021). For patients with severe carotid artery stenosis and apparent symptoms, surgical treatment such as carotid endarterectomy or carotid artery stenting can be considered (CSN, 2017; Naylor et al., 2018). Statins are important drugs for regulating blood lipids and stabilizing plaques. However, these may cause adverse effects such as liver function damage, myopathy or hyperglycemia (Auer et al., 2016; Wang et al., 2022b). Hypoglycemic drugs such as biguanides, sodium-glucose cotransporter-2 (SGLT-2) inhibitors and glucagon-like peptide-1 (GLP-1) receptor agonists may have potential effects on atherosclerosis when lowering blood glucose (Liu et al., 2021; Jonik et al., 2022; Poznyak et al., 2022). However, there may be adverse effects such as gastrointestinal discomfort, decreased vitamin B12 concentration or urinary tract infection (Baye et al., 2021). Although surgical intervention could relieve severe carotid stenosis, it cannot prevent the formation and development of atherosclerosis from the source (Copur et al., 2021; Yao et al., 2022). Considering the high risk during surgery and postoperative vascular restenosis, whether interventional therapy can bring sufficient benefit to patients is still controversial (Naylor et al., 2018; Wangqin et al., 2019; Achim et al., 2022; Reiff et al., 2022). Thus, there is an urgent need to find safer and more effective treatments for T2DM patients with carotid atherosclerosis.

Chinese herbal medicine (CHM), one of the important therapeutic methods in traditional Chinese medicine (TCM), has a complete theoretical system and has long been used in combination with Western medicine (WM) to treat various diseases (Wang et al., 2013; Zhou et al., 2022). CHM is mainly derived from traditional botanicals, animals and minerals, of which botanicals are the most common. Over the years, researchers have explored the effects of CHM in treating T2DM patients with CAS. Results from case reports, retrospective studies and randomized controlled trials (RCTs) suggest that CHM may be able to reduce the atherosclerosis degree, regulate glucose and lipid metabolism, improve vascular endothelial cell function and relieve clinical symptoms in T2DM patients with CAS (Yang, 2007; Zhou et al., 2011; He, 2013; Zhang et al., 2014; Cheng, 2016; Lu et al., 2022). However, the clinical efficacy remains uncertain due to limited sample size, inconsistent trial design and uneven trial quality. A previous meta-analysis showed that CHM is relatively safe and effective in treating T2DM patients with CAS (Huang, 2015). However, this study could not provide reliable results due to methodological flaws such as lack of registration, incomplete database searches and neglect on heterogeneity analysis. Some new clinical studies have been conducted since the publication of this study, making it necessary to update the evidence. Therefore, we collected RCTs rigorously and systematically evaluated the clinical efficacy and safety of CHM in treating T2DM patients with CAS, aiming to summarize the latest evidence and provide reference for subsequent research and clinical practice.

## Materials and methods

### Study registration

This systematic review and meta-analysis were performed under the guidance of the Cochrane Handbook for Systematic Reviews of Interventions version 6.3 (updated 2022) (Cochrane, 2022) and the Preferred Reporting Items for Systematic Review and Meta-Analysis (PRISMA) 2020 Statement (Moher et al., 2009; Page et al., 2021). The PRISMA 2020 checklist is provided in Supplementary Material S1. Before starting, this study was registered in the International Prospective Register of Systematic Reviews (PROSPERO) (Registration number: CRD42022346274). Data were derived from published clinical studies.

### Database and search strategies

We conducted a comprehensive search of eight electronic databases, including PubMed, EMBASE, Cochrane Library, Web of Science (WOS), China National Knowledge Infrastructure (CNKI), Wan Fang Database, China Science and Technology Journal Database (VIP) and the China Biomedical Medicine database (CBM), from their inceptions to July 2022. The clinical trials evaluating the efficacy and safety of CHM in treating T2DM patients with CAS were retrieved using a combination of subject terms and text words. The search terms mainly included: “Medicine, Chinese Traditional”, “Chung I Hsueh”, “Zhong Yi Xue”, “Chinese Herbal Drugs”, “Type 2 Diabetes Mellitus”, “Diabetes Mellitus, Type 2”, “Type 2 Diabetes”, “Non-insulin-Dependent Diabetes Mellitus”, “Carotid Artery Diseases”, “Carotid Atherosclerosis”, “Carotid Atherosclerotic Plaque” and “Carotid Intima-Media Thickness”. The detailed search strategies containing more search terms are provided in Supplementary Material S2. To understand ongoing studies, the ClinicalTrials.gov database and Chinese Clinical Trial Registry (CHiCTR) were also retrieved. Additionally, references of related reviews and meta-analyses were also screened to discover literature that may be missed in online searches. All literature was selected according to inclusion criteria and exclusion criteria.

### Inclusion criteria

#### Type of studies

RCTs were included without restriction on countries or publication language.

#### Type of participants

Adults (≥18 years old) with a definite diagnosis of T2DM with CAS were included, and there were no restrictions on age, race or gender.

#### Type of interventions and comparisons

The intervention was any orally administered CHM, including Chinese herbal compounds, Chinese patent medicine, single herb or Chinese herbal extracts, without limit to dosage form (decoction, pills, granules or capsules), dosage or treatment duration. The patients in the experimental group were treated with CHM combined with WM, and the control group received the same WM alone or in combination with placebo. WM included hypoglycemic drugs, lipid-lowering drugs, anti-platelet aggregation drugs and drugs for other underlying diseases. These drugs must be consistently recommended by experts.

#### Type of outcome measures

To comprehensively evaluate the efficacy and safety of CHM in the treatment of T2DM patients with CAS, RCTs assessing any of the following outcomes were included.

1) Primary Outcomes

Carotid intima-media thickness (CIMT), Carotid plaque Crouse score.

2) Secondary Outcomes

Lipid metabolism index: Total Cholesterol (TC), Triglyceride (TG), High-Density Liptein Cholesterol (HDL-C), Low-Density Lipoprotein Cholesterol (LDL-C).

Glucose metabolism index: Fasting blood glucose (FBG), 2-h postprandial glucose (2hPG), Glycated hemoglobin (HbA1c).

Vascular endothelial function and vascular resistance: Nitric oxide (NO), Endothelin-1 (ET-1), Peak systolic velocity (PSV), Resistance index (RI).

Insulin resistance index: Homeostasis model assessment of insulin resistance (HOMA-IR).

Pancreatic islet function index: Homeostasis model assessment of beta-cell function (HOMA-β).

If a study reported multiple time points, the result with the longest time point was included in the analysis.

3) Safety Outcomes

Any adverse events that occurred during the study should be recorded, such as the incidence of gastrointestinal reactions, the incidence of abnormal liver function, the incidence of hypoglycemia, the incidence of adverse events and the incidence of serious adverse events.

### Exclusion criteria

#### Type of studies

1) Studies designed as non-RCTs, such as cohort studies, case-control studies, cross-sectional studies, case reports and reviews.
2) Basic studies, such as experiments on animals, cells or tissues.
3) Meeting abstracts were excluded if no relevant data were provided.
4) Studies were excluded if the full text could not be obtained by searching online or contacting the authors.

#### Type of participants

1) Indicators related to CAS were reported, while the study subjects were not T2DM patients with CAS.
2) Carotid ultrasound showed complete occlusion of the bilateral carotid lumen.
3) Patients with acute metabolic disorders, such as diabetic ketoacidosis or infections.
4) Patients with severe hepatic and renal impairment, severe cardiovascular or cerebrovascular diseases, pregnancy or lactation were excluded.

#### Type of interventions and comparisons

1) The interventions used TCM treatments other than CHM, such as acupuncture, moxibustion, massage or acupoint injection.
2) The control group used measures other than WM.

#### Type of outcome measures

Studies with obvious data errors, incomplete data, questionable authenticity and lack of required indicators were excluded. Obvious errors refer to inconsistent descriptions of data in the context, articles published before the completion of trial, incorrect choice in statistical method or random grouping method.

### Study selection and data extraction

The search results were imported into EndNote X9 software in the form of bibliography to establish a database. Two researchers independently screened the literature according to the inclusion and exclusion criteria. Firstly, duplicate literature was deleted. Secondly, the literature that did not meet the criteria was excluded by reading the title and abstract. The literature that was uncertain in the preliminary screening was read in full. After reading the full text, literature that still did not meet the criteria was excluded. If there was any difference, it was determined after discussion or consultation with XF. Two researchers independently extracted data from included studies according to the pre-designed data extraction table. If some additional data were needed, we contacted the authors by email. The research data extracted mainly included: first author, publication year, study design, study period, sample size, gender, average age, course of disease, body mass index (BMI), treatment duration, intervention measures, outcome indicators, comorbidity, adverse events and was cross-checked. If the same RCT has published several articles successively, the one published first was selected.

### Risk of bias assessment

The quality of the literature was assessed using the bias risk assessment tool in Review Manager 5.4. The assessment content consists of seven domains, including random sequence generation, allocation concealment, blinding of participants and personnel, blinding of outcome assessment, incomplete outcome data, selective reporting and other bias. The bias risk assessment was carried out for each included study from these seven domains. The judgment of each domain was divided into “high risk”, “low risk” and “unclear risk”. ZZ and YL performed independently and examined each other. If there were different opinions on the evaluation results, it was determined after discussion or consultation with XF.

### Statistical analysis

Review Manager 5.4 and Stata 16.0 were used for meta-analysis. For binary variables, the relative risk (RR) and 95% confidence interval (CI) were used to express the effect size. For continuous variables, when the same outcome indicator used the same unit, the mean difference (MD) and 95% CI were used to represent the effect size; otherwise, the standardized mean difference (SMD) and 95% CI were used. Heterogeneity was evaluated according to χ^2^ test and I^2^ test. If *p* > 0.1, I^2^<50%, it indicated that the heterogeneity between studies was small, and the fixed effect model was used to calculate the pooled effect size. If *p* ≤ 0.1, I^2^ ≥ 50%, it suggested significant statistical heterogeneity among the studies; therefore, the random effect model was used. If more than 10 studies were included for a study indicator, meta-regression was used to analyze sources of heterogeneity; otherwise, subgroup analysis was used. Sensitivity analysis was performed to judge the stability of the research results. Meanwhile, we performed funnel plot and Egger’s test to evaluate publication bias for indicators that included more than 10 studies. *p* > 0.05 indicated no obvious publication bias, and *p* < 0.05 indicated possible publication bias. Finally, we used the Grading of Recommendations Assessment, Development and Evaluation (GRADE) method to assess the evidence quality.

### Subgroup analysis and meta-regression

Subgroup analyses or meta-regressions were performed according to the following factors to explore the sources of heterogeneity and to discover the factors that might influence the effect: Average age (≤60 years old or >60 years old); Treatment duration (<6 months or ≥6 months); CHM dosage form (decoction or other dosage form).

### Identification of commonly used Chinese herbal medicines

We summarized the CHMs used in all included studies and sorted CHMs according to occurrence frequency to explore the commonly used CHMs.

## Results

### Database search results

**FIGURE 1:**
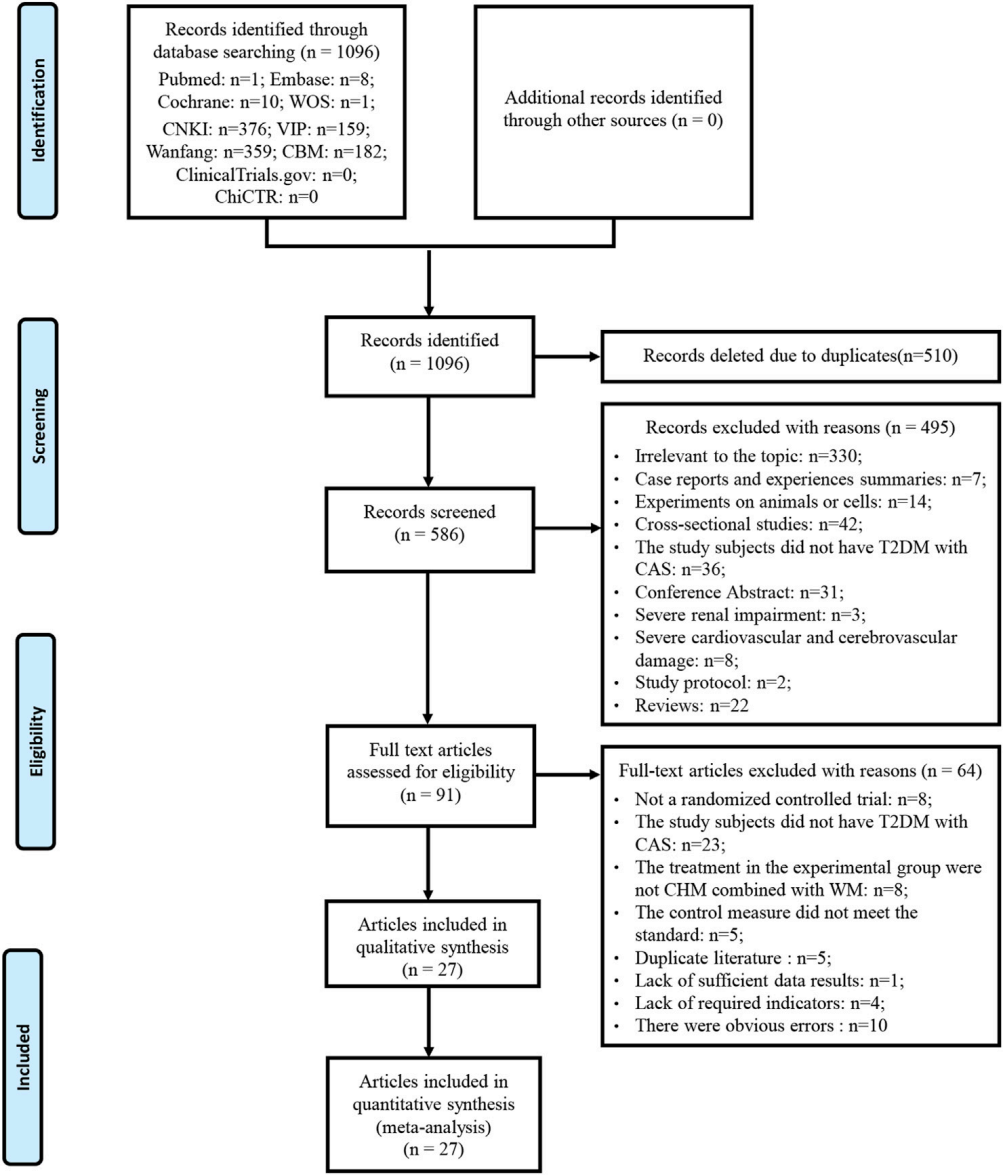
Flowchart of study selection and identification.

A total of 1096 articles were obtained by searching PubMed, EMBASE, Cochrane Library, WOS, CNKI, Wan Fang Database, VIP and the CBM. Five hundred and ten articles were removed due to duplication. Of the remaining 586 articles, 495 articles were excluded after reading titles and abstracts. By reading the full text of the remaining 91 articles, 64 articles were excluded according to the inclusion and exclusion criteria. No additional studies were identified by screening references of relevant reviews and meta-analyses. A total of 27 studies were included after removing duplicates published by the same study. Literature excluded after reading the full text and reasons is listed in Supplementary Material S3. A detailed flowchart for screening eligible studies is shown in Figure 1.

### Characteristics of included studies

A total of 27 RCTs were included in this study, all completed in China and published between 2011 and 2021 (Sun and Fan, 2011; Zhou et al., 2011; Deng et al., 2012; Shao, 2012; Fang et al., 2013; Li, 2013; Wang, 2013; Yang, 2013; Zuo et al., 2013; Fu, 2014; Li et al., 2014; Luo, 2015; Sun et al., 2015; Yang et al., 2016b; Cong, 2016; Lu et al., 2016; Lyu et al., 2016; Shi and Lu, 2016; Xu, 2016; Chen et al., 2017; Yang, 2017; Bu et al., 2018; Fan et al., 2020; Guo, 2020; Mao, 2020; Wang et al., 2021c; Jiao et al., 2021). A total of 2638 T2DM patients with CAS were included in this study, including 1324 in experimental group and 1314 in control group. The sample size of each study ranged from 50 to 268, the average age ranged from 43.52 to 81.13 years old and the treatment duration ranged from 1 to 12 months. In the control group, 26 studies (Sun and Fan, 2011; Zhou et al., 2011; Deng et al., 2012; Shao, 2012; Fang et al., 2013; Li, 2013; Wang, 2013; Yang, 2013; Zuo et al., 2013; Fu, 2014; Li et al., 2014; Sun et al., 2015; Yang et al., 2016b; Cong, 2016; Lu et al., 2016; Lyu et al., 2016; Shi and Lu, 2016; Xu, 2016; Chen et al., 2017; Yang, 2017; Bu et al., 2018; Fan et al., 2020; Guo, 2020; Mao, 2020; Wang et al., 2021c; Jiao et al., 2021) used WM alone and 1 study (Luo, 2015) used WM plus placebo. The intervention in the experimental group was a combination with CHM based on the WM. 25 studies used Chinese herbal compounds or Chinese patent medicine (Sun and Fan, 2011; Zhou et al., 2011; Deng et al., 2012; Shao, 2012; Fang et al., 2013; Li, 2013; Wang, 2013; Yang, 2013; Zuo et al., 2013; Fu, 2014; Li et al., 2014; Luo, 2015; Sun et al., 2015; Cong, 2016; Lu et al., 2016; Lyu et al., 2016; Shi and Lu, 2016; Xu, 2016; Chen et al., 2017; Yang, 2017; Bu et al., 2018; Fan et al., 2020; Mao, 2020; Wang et al., 2021c; Jiao et al., 2021), all composed of multiple herbs. The remaining two studies used a single herb (Yang et al., 2016b; Guo, 2020). CHM was taken in the form of decoction in 13 studies (Sun and Fan, 2011; Deng et al., 2012; Li, 2013; Wang, 2013; Yang, 2013; Cong, 2016; Lu et al., 2016; Xu, 2016; Chen et al., 2017; Yang, 2017; Bu et al., 2018; Wang et al., 2021c; Jiao et al., 2021), capsule in nine studies (Zhou et al., 2011; Fang et al., 2013; Zuo et al., 2013; Li et al., 2014; Luo, 2015; Sun et al., 2015; Lyu et al., 2016; Shi and Lu, 2016; Mao, 2020), granule in two studies (Fu, 2014; Yang et al., 2016b), powder in two study (Shao, 2012; Guo, 2020) and pill in 1 study (Fan et al., 2020). The basic characteristics of the included studies are shown in Table 1 and the detailed components of CHM are shown in Table 2.

**TABLE 1:** The characteristics of the included studies.

First author (year)	Zhou et al. (2011)	Sun and Fan (2011)	Deng et al. (2012)	Shao (2012)	Fang et al. (2013)
Study design	RCT	RCT	RCT	RCT	RCT
Study period	2008.3–2009.12	2007.3–2009.5	2009.7–2011.6	2010.8–2011.3	2011.3–2011.12
Sample size (randomized/analyzed) (E/C)	62/62; 33/29	58/58; 30/28	60/60; 30/30	50/50; 25/25	196/179; 92/87
Gender (M/F) (E/C)	16/17; 15/14	37/21	18/12; 17/13	16/9; 15/10	58/40; 56/42
Average age (years) (E/C)	69.4 ± 6.5; 69.7 ± 6.0	60.4 ± 4.6	60–89; 61–90	35–70/35–70	57.56 ± 9.89; 58.24 ± 10.43
Course of disease (years) (E/C)	13.0 ± 5.5; 13.0 ± 6.0	4–16	5.0–8.4; 4.8–8.0	NR	6.74 ± 4.67; 7.11 ± 8.20
BMI	25.2 ± 2.6; 24.8 ± 2.8	24.6 ± 0.2	NR	NR	NR
Treatment duration	8 weeks	12 weeks	4 weeks	12 months	12 months
Co-intervention	NR	NR	NR	Diabetes health education + Diet and exercise intervention	Diet and exercise intervention
Treatment group interventions	Xiaoke Huayu capsule, 6 capsules/per, tid + CG	Huatan Tongluo decoction, 1 dose/per day, bid + CG	Qinghua Xiaoyu decoction, 1 dose/per day, 200 mL/per, bid + CG	Qimai Shenlian powder, 3 g/per, tid + CG	Danzhi Jiangtang capsule, 9 g, qn + CG
Control group interventions	Hypoglycemic drugs + Hypolipidemic drugs + Antihypertensive drugs + Antiplatelet aggregation drugs	Insulin subcutaneous injection or oral hypoglycemic drugs + Simvastatin 20 mg qd	Hypoglycemic drugs + Hypolipidemic drugs	Hypoglycemic drugs + Hypolipidemic drugs + Antihypertensive drugs	Hypoglycemic drugs + Atorvastatin 20 mg qn
Outcome index	①⑩⑪	①③④⑤⑥	①③④⑤⑦⑧⑨	①	①③④⑤⑥⑦⑨⑭
Baseline difference	NSD	NSD	NSD	NSD	NSD
Comorbidity	NR	NR	NR	NR	NR
Adverse events	NR	NR	NR	No obvious discomfort was found after taking medicine. No abnormality was found in safety indicators	Treatment group: 8 gastrointestinal reaction, 1 fatigue; Control group: 1 headache, 4 gastrointestinal reaction. All adverse events were mild and resolved after symptomatic treatment
Country	China	China	China	China	China
Funding	NR	NR	Shenzhen Science and Technology Plan Project, No.200903215	NR	National TCM Clinical Research Base Construction Project, No.20081211. National Key Discipline of TCM, No.20091221. National Science and Technology Support Program of the Ministry of Science and Technology, No.2012BA126B00

First author (year)	Li (2013)	Wang (2013)	Yang (2013)	Zuo et al. (2013)	Fu (2014)
Study design	RCT	RCT	RCT	RCT	RCT
Study period	2011.4–2012.10	2011.10–2012.11	NR	2010.1–2011.6	2013.4–2013.12
Sample size (randomized/analyzed) (E/C)	50/50; 25/25	62/60; 30/30	64/61; 31/30	99/99; 50/49	50/50; 25/25
Gender (M/F) (E/C)	14/11; 13/12	20/10; 18/12	17/14; 16/14	32/18; 29/20	14/11; 14/11
Average age (years) (E/C)	55.82 ± 6.37	81.13 ± 6.06; 79.57 ± 8.07	69.45 ± 4.71; 71.59 ± 5.59	58.16 ± 5.78; 56.61 ± 5.42	63.48 ± 9.78; 65.68 ± 9.65
Course of disease (years) (E/C)	NR	NR	7.17 ± 4.97; 7.53 ± 4.20	15.20 ± 5.32; 15.78 ± 4.75	9.41 ± 7.09; 10.28 ± 8.34
BMI	NR	NR	NR	25.40 ± 2.65; 25.50 ± 2.00	24.09 ± 3.33; 24.08 ± 3.04
Treatment duration	12 months	8 weeks	8 weeks	6 months	6 months
Co-intervention	Diabetes health education + Diet and exercise intervention	NR	Diabetes health education + Diet and exercise intervention	Diet and exercise intervention	Diabetes health education + Diet intervention
Treatment group interventions	Tongluo Jiedu decoction, 1 dose/per day, 200 mL/per, bid + CG	Zishui Tongmai decoction, 100 mL/per, bid + CG	Bushen Yiqi Huoxue decoction, 1 dose/per day, 400 mL, bid + CG	Jinchai Xiaoke capsule, 5 capsules/per, tid + CG	Luomaitong granule + CG
Control group interventions	Hypoglycemic drugs	Simvastatin 40 mg qd + Aspirin 100 mg qd + Hypoglycemic drugs + Antihypertensive drugs	Hypoglycemic drugs	Insulin subcutaneous injection or oral hypoglycemic drugs. If combined with hyperlipidemia, atorvastatin was given at 20 mg once a day	Insulin subcutaneous injection or oral hypoglycemic drugs + Atorvastatin 20 mg qn + Aspirin 0.1 g qn
Outcome index	①③④⑤⑥⑦⑧⑩⑪	①②③④⑤⑥⑦⑨	①③④⑤⑥⑦⑧⑨⑩⑪	①③④⑤⑥	③④⑤⑥⑦⑧⑨⑫⑬⑭
Baseline difference	NSD	NSD	NSD	NSD	NSD
Comorbidity	NR	Hypertension or hyperlipidemia	Coronary heart disease, hypertension or hyperlipidemia	NR	Hypertension
Adverse events	No obvious discomfort was found after taking medicine, and no abnormality was found in safety indicators	No obvious abnormality was found in safety indicators after treatment. No adverse reactions occurred	Treatment group: 2 diarrhea; Control group: 1 dizziness. All of which resolved spontaneously without treatment. No obvious abnormality was found in safety indicators	NR	No obvious discomfort was found after taking medicine, and no abnormality was found in vital signs and safety indicators
Country	China	China	China	China	China
Funding	NR	Fund Project of Yueyang Hospital of Integrated Traditional Chinese and Western Medicine, No.30304115248	NR	Xuzhou Science and Technology Bureau Social Development Project, No.XF10C024	NR

First author (year)	Li et al. (2014)	Sun et al. (2015)	Luo (2015)	Cong (2016)	Lu et al. (2016)
Study design	RCT	RCT	RCT	RCT	RCT
Study period	2011.2–2013.9	2013.4–2015.4	2012.8–2013.8	2014.10–2015.10	2014.3–2015.12
Sample size (randomized/analyzed) (E/C)	240/240; 120/120	205/205; 103/102	136/124; 64/60	60/60; 30/30	60/60; 30/30
Gender (M/F) (E/C)	75/45; 67/53	57/46; 58/44	36/32; 38/30	15/15; 11/19	18/12; 20/10
Average age (years) (E/C)	58.2 ± 10.9; 57.9 ± 12.2	46.7 ± 11.5; 45.8 ± 10.8	57.26 ± 5.00; 56.26 ± 6.01	57.77 ± 1.29; 58.50 ± 1.10	79.13 ± 7.65; 80.7 ± 6.11
Course of disease (years) (E/C)	NR	NR	7.62 ± 2.15; 7.73 ± 2.15	7.17 ± 4.97; 7.53 ± 4.20	14.15 ± 6.52; 14.38 ± 7.42
BMI	26.5 ± 2.48; 26.2 ± 2.35	NR	23.59 ± 1.62; 23.72 ± 1.50	NR	NR
Treatment duration	6 months	3 months	12 months	12 weeks	3 months
Co-intervention	Diet and exercise intervention	Diet and exercise intervention	Diet and exercise intervention	Diabetes health education + Diet and exercise intervention	NR
Treatment group interventions	Tongxinluo capsule, 2 capsules/per, tid + CG	Tongxinluo capsule, 4 capsules/per, tid + CG	Danzhi Jiangtang capsule, 3 capsules, tid + CG	Jianpi Tongmai decoction, 1 dose/day, 200mL/per, bid + CG	Modified Simiao Yongan decoction + CG
Control group interventions	Insulin subcutaneous injection or oral hypoglycemic drugs + Atorvastatin 20 mg qn	Insulin subcutaneous injection or oral hypoglycemic drugs + Rosuvastatin 20 mg qn	Metformin 0.5 g bid or glimepiride 2 mg qd or gliclazide 30 mg qd + Atorvastatin 10 mg qn + Placebo 3 capsules tid	Hypoglycemic drugs + Simvastatin 20 mg qn	Insulin subcutaneous injection or oral hypoglycemic drugs + Hypolipidemic drugs
Outcome index	①②③④⑤⑥⑦⑧⑨⑭	③④⑤⑥⑦⑧⑨⑭⑮	①③④⑤⑥⑦⑧⑨⑩⑪	①③④⑤⑥⑦⑧⑨	①②③④⑦⑨
Baseline difference	NSD	NSD	NSD	NSD	NSD
Comorbidity	NR	NR	NR	NR	NR
Adverse events	Treatment group: 8 gastrointestinal reaction, 3 abnormal liver function, 2 abnormal renal function; Control group: 7 gastrointestinal reaction, 2 abnormal liver function, 1 abnormal renal function. The incidence of adverse reactions in the two groups was not statistically significant	Treatment group: 3 nausea and vomiting, 3 dizziness and headache, 1 skin itching; Control group: 2 nausea and vomiting, 2 dizziness and headache. The incidence of adverse reactions in the two groups was not statistically significant	Treatment group: 6 gastrointestinal reaction, 4 skin pruritus, 2 hypoglycemia reaction; Control group: 4 gastrointestinal reaction, 2 skin pruritus, 4 hypoglycemia reaction. Abdominal distention disappeared after continuing to take medicine, hypoglycemia reaction was relieved after eating and the skin itching disappeared without intervention	No adverse reactions occurred during treatment, and no significant abnormality was found in the safety indicators	NR
Country	China	China	China	China	China
Funding	NR	NR	NR	NR	Jinhua TCM Science and Technology Research Project, No. 2014-4-014

First author (year)	Lyu et al. (2016)	Shi and Lu (2016)	Xu (2016)	Yang et al. (2016b)	Chen et al. (2017)	Yang (2017)
Study design	RCT	RCT	RCT	RCT	RCT	RCT
Study period	2012.1–2014.12	2014.1–2015.1	2015.3–2015.11	2011.9–2014.9	2013.9–2016.9	2016.3–2017.3
Sample size (randomized/analyzed) (E/C)	120/120; 60/60	62/60; 30/30	60/60; 30/30	152/152; 76/76	268/257; 124/133	60/60; 30/30
Gender (M/F) (E/C)	31/29; 32/28	15/16; 16/15	14/16; 15/15	45/31; 43/33	63/69; 65/71	19/11; 21/9
Average age (years) (E/C)	58.1 ± 2.7; 52.1 ± 3.7	64.1 ± 4.9; 63.8 ± 5.1	49.40 ± 9.18; 53.53 ± 7.86	59.6 ± 10.9; 60.2 ± 9.3	64.5 ± 12.8; 63.8 ± 13.6	57.07 ± 7.49; 57.37 ± 6.61
Course of disease (years) (E/C)	NR	12.9 ± 6.3; 13.7 ± 4.5	NR	NR	10.6 ± 5.5; 11.3 ± 4.8	9.27 ± 4.55; 9.67 ± 4.69
BMI	26.3 ± 2.5; 26.7 ± 2.6	NR	NR	NR	NR	NR
Treatment duration	6 months	24 weeks	6 months	6 months	12 weeks	3 months
Co-intervention	Diabetes health education + Diet and exercise intervention + Blood glucose monitoring	Diabetes health education + Diet and exercise intervention	Diet and exercise intervention	Lifestyle intervention	Diabetes health education + Diet and exercise intervention	Diabetes health education + Diet intervention
Treatment group interventions	Tongxinluo capsule, 2 capsules/per, tid + CG	Danzhi Jiangtang capsule, 3 capsules/per, tid + CG	Jianpi Xiaozhi decoction, 1 dose/per day, 200 mL, bid + CG	Hawthorn formula granule, 1 package, qn + CG	Yishen Huoxue Huatan decoction, 1 dose/per day, 400 mL, bid + CG	Jiawei Zicui Tongmai decoction, 1 dose/per day, 200 mL, bid + CG
Control group interventions	Insulin subcutaneous injection or oral hypoglycemic drugs + Atorvastatin 20 mg qn	Hypoglycemic drugs (metformin or acarbose or glimepiride or gliclazide) + Atorvastatin	Hypoglycemic drugs + Atorvastatin 20 mg qd	Insulin subcutaneous injection, acarbose was added to patients with high postprandial blood glucose + Simvastatin 20 mg qn	Oral hypoglycemic drugs. Based on the condition, hypolipidemic drugs, antiplatelet aggregation drugs, antihypertensive drugs and other symptomatic treatments were given	Insulin or oral hypoglycemic drugs were given to maintain FBG<7 mmol/L and 2hPG<10 mmol/L + Atorvastatin 10 mg qn
Outcome index	①③④⑤⑥⑦⑧⑨⑭	①③④⑤⑦⑧⑨	①③④⑤⑥⑦⑭	①③④⑤⑥⑦⑨	①③④⑤⑦⑨⑫⑬	①③④⑤⑥ ⑦⑧⑨
Baseline difference	NSD	NSD	NSD	NSD	NSD	NSD
Comorbidity	NR	NR	NR	NR	Hypertension, stable coronary heart disease, chronic gastritis, lacunar cerebral infarction or fatty liver	NR
Adverse events	NR	NR	There were no adverse events in the treatment group. Control group: 1 diarrhea, 1 anorexia. The symptoms were relieved after stopping the drug	After taking medicine, the liver and kidney functions were all within the normal range, and there were no adverse reactions	Treatment group: 1 abdominal distension and nausea. Control group: 2 abnormal liver function	Treatment group: No significant abnormality was found in vital signs and safety indicators. Control group: 2 mild transaminase elevation, no special treatment was given
Country	China	China	China	China	China	China
Funding	NR	NR	NR	NR	NR	NR

First author (year)	Bu et al. (2018)	Fan et al. (2020)	Guo (2020)	Mao (2020)	Jiao et al. (2021)	Wang et al. (2021c)
Study design	RCT	RCT	RCT	RCT	RCT	RCT
Study period	2016.10–2017.7	2016.1–2018.8	2018.4–2019.3	2017.5–2018.5	2017.4–2019.4	2018.1–2019.12
Sample size (randomized/analyzed) (E/C)	60/60; 30/30	70/67; 34/33	60/60; 30/30	126/126; 63/63	98/98; 49/49	100/100; 50/50
Gender (M/F) (E/C)	18/12; 14/16	19/16; 17/18	19/11; 16/14	30/33; 29/34	28/21; 29/20	31/19; 33/17
Average age (years) (E/C)	43.52 ± 4.83; 43.78 ± 4.26	58 ± 10.12; 57 ± 10.23	63.76 ± 12.02; 63.10 ± 11.62	45.4 ± 10.1; 46.1 ± 10.9	61.46 ± 4.49; 61.44 ± 4.51	67.33 ± 7.29; 66.52 ± 7.33
Course of disease (years) (E/C)	9.47 ± 1.62; 9.46 ± 1.64	NR	9.36 ± 2.90; 9.23 ± 4.61	NR	7.09 ± 1.43; 7.11 ± 1.45	NR
BMI	NR	NR	25.56 ± 4.01; 24.52 ± 3.51	NR	26.09 ± 0.62; 26.11 ± 0.64	NR
Treatment duration	4 weeks	12 months	6 months	3 months	8 weeks	3 months
Co-intervention	Diet intervention	Diet and exercise intervention	Diabetes health education + Diet intervention	Diet and exercise intervention	Diet and exercise intervention	NR
Treatment group interventions	Modified Taohong Siwu decoction, 200 mL, qd + CG	Tongmai Yuban pill, 10g/per, tid + CG	Notoginseng powder, 3 g qd + CG	Yudan Shengui capsule, 4 capsules/per, tid + CG	Modified Yuye decoction, 1 dose/per day, 300mL, bid + CG	Furong Tongmai decoction, 1 dose/per day, bid + CG
Control group interventions	Insulin 10u qd subcutaneous injection or oral hypoglycemic drugs were given to maintain FBG within 4.0–7.0 mmol/L and 2hPG within 4.0–10.0 mmol/L	Aspirin 100 mg qd + Atorvastatin 10 mg qn + Metformin 500 mg qd	Insulin or oral hypoglycemic drugs + Aspirin 100 mg qn + Atorvastatin 20 mg qn	Insulin or oral hypoglycemic drugs + Rosuvastatin 20 mg qd	Insulin or oral hypoglycemic drugs + Aspirin 100 mg qd. If dyslipidemia, atorvastatin was given at 10 mg per day	Hypoglycemic drugs + Antihypertensive drugs + Simvastatin 20 mg qd
Outcome index	①⑦⑧⑨	①⑤⑦⑨	①③④⑤⑥⑦⑧⑨	①②③④⑤⑥	①⑦⑧⑨	①⑦⑧⑨
Baseline difference	NSD	NSD	NSD	NSD	NSD	NSD
Comorbidity	NR	Hypertension or coronary heart disease	NR	NR	Hypertension, hyperlipidemia or osteoporosis	Hypertension or hyperlipidemia
Adverse events	NR	There was no obvious abnormality in safety indicators in both groups after treatment	During the treatment, the safety indicators were all within the normal range, and the patient had no adverse reactions	Treatment group: no obvious abnormality in safety indicators. Control group: 1 nausea and vomiting, 1 dizziness and headache	NR	NR
Country	China	China	China	China	China	China
Funding	NR	Henan Province TCM Scientific Research Special Project, No.2015ZY02055	NR	NR	Shandong Province Medical and Health Science and Technology Development Project, No.2015QZ008	Subject of Hebei Science and Technology Department, No.17277786D

Abbreviations: RCT, randomized controlled trial; NR, not reported; CG: control group interventions; NSD, no significant difference; TCM, traditional Chinese medicine; Outcome index, ①CIMT; ②Crouse score; ③TC; ④TG; ⑤LDL-C; ⑥HDL-C; ⑦FBG; ⑧2hPG; ⑨HbA1c; ⑩NO; ⑪ET-1; ⑫PSV; ⑬RI; ⑭HOMA-IR; ⑮HOMA-β.

**TABLE 2:** Detailed components of CHM.

Study	Prescription name	Ingredients of herb prescription	Preparations
Zhou et al. (2011)	Xiaoke Huayu capsule	Figwort Root (Xuanshen, Scrophularia ningpoensis Hemsl.) 15 g, Dwarf Lilyturf Tuber (Maidong, Ophiopogon japonicus (Thunb.) Ker Gawl.) 10g, Chinese Magnoliavine Fruit (Wuweizi, Schisandra chinensis (Turcz.) Baill.) 10g, Milkvetch Root (Huangqi, Astragalus mongholicus Bunge) 30 g, Scorpion (Quanxie, Scorpio) 6g, Hawthorn Fruit (Shanzha, Crataegus pinnatifida Bunge) 15 g, Earthworm (Dilong, Pheretima) 10 g, Twotoothed Achyranthes Root (Niuxi, Achyranthes bidentata Blume) 20 g, Danshen Root (Danshen, Salvia miltiorrhiza Bunge) 30 g and Safflower (Honghua, Carthamus tinctorius L.) 10 g	Capsule
Sun and Fan (2011)	Huatan Tongluo decoction	Milkvetch Root (Huangqi, Astragalus mongholicus Bunge) 20 g, Hawthorn Fruit (Shanzha, Crataegus pinnatifida Bunge) 20 g, Szechwan Lovage Rhizome (Chuanxiong, Ligusticum striatum DC.) 10 g, Rhizoma Curcumae (Ezhu, Curcuma aromatica Salisb.) 10g, Virgate Wormwood Herb (Yinchen, Artemisia capillaris Thunb.) 15g, Danshen Root (Danshen, Salvia miltiorrhiza Bunge) 15g, Coix Seed (Yiyiren, Coix lacryma-jobi L.) 30 g and Liquorice Root (Gancao, Glycyrrhiza glabra L.) 5 g. If numbness and pain of limbs was identified, Frankincense (Ruxiang, Boswellia sacra Flück.) 10 g and Common Clubmoss Herb (Shenjincao, Lycopodium japonicum Thunb.) 20 g were added. If yellow greasy coating was identified, Chinese Cork-tree (Huangbai, Phellodendron amurense Rupr.) 10 g and Herba Plantaginis (Cheqiancao, Plantago asiatica L.) 15 g were added. If emotional disorder was identified, Chinese Thorowax Root (Chaihu, Bupleurum falcatum L.) 10 g and White Paeony Root (Baishao, Paeonia lactiflora Pall.) 15 g were added	Decoction
Deng et al. (2012)	Qinghua Xiaoyu decoction	Milkvetch Root (Huangqi, Astragalus mongholicus Bunge) 20 g, Tangshen (Dangshen, Codonopsis pilosula (Franch.) Nannf.) 15 g, Hawthorn Fruit (Shanzha, Crataegus pinnatifida Bunge) 10 g, Baical Skullcap Root (Huangqin, Scutellaria baicalensis Georgi) 10 g, Purslane Herb (Machixian, Portulaca oleracea L.) 15 g, Giant Knotweed Rhizome (Huzhang, Reynoutria japonica Houtt.) 10 g, Fleeceflower Root (Heshouwu, Reynoutria multiflora (Thunb.) Moldenke) 15 g, Oriental Water Plantain Rhizome (Zexie, Alisma plantago-aquatica L.) 10 g, Sweet Wormwood Herb (Qinghao, Artemisia annua L.) 10 g, Rhubarb (Dahuang, Rheum palmatum L.) 5 g, Danshen Root (Danshen, Salvia miltiorrhiza Bunge) 8 g and Largehead Atractylodes Rh (Baizhu, Atractylodes macrocephala Koidz.) 10 g	Decoction
Shao (2012)	Qimai Shenlian powder	Milkvetch Root (Huangqi, Astragalus mongholicus Bunge), Dwarf Lilyturf Tuber (Maidong, Ophiopogon japonicus (Thunb.) Ker Gawl.), Danshen Root (Danshen, Salvia miltiorrhiza Bunge), Rhizoma Coptidis (Huanglian, Coptis chinensis Franch.), Honeysuckle Flower (Jinyinhua, *Lonicera japonica* Thunb.) and Pseudo-ginseng (Sanqi, Panax notoginseng (Burkill) F.H.Chen). Proportion: 10: 10: 10: 3: 10: 1	Powder
Fang et al. (2013)	Danzhi Jiangtang capsule	Heterophylly Falsestarwort Root (Taizishen, Pseudostellaria heterophylla (Miq.) Pax), Rehmannia Root (Dihuang, Rehmannia glutinosa (Gaertn.) DC.), Tree Peony Bark (Mudanpi, Paeonia × suffruticosa Andrews), Dodder Seed (Tusizi, Cuscuta chinensis Lam.), Oriental Water Plantain Rhizome (Zexie, Alisma plantago-aquatica L.) and Leech (Shuizhi, Hirudo)	Capsule
Li (2013)	Tongluo Jiedu decoction	Milkvetch Root (Huangqi, Astragalus mongholicus Bunge), Rehmannia Root (Dihuang, Rehmannia glutinosa (Gaertn.) DC.), Danshen Root (Danshen, Salvia miltiorrhiza Bunge), Thomson Kudzuvine Root (Gegen, Pueraria montana var. thomsonii (Benth.) M.R.Almeida), Rhizoma Coptidis (Huanglian, Coptis chinensis Franch.) and Honeysuckle Flower (Jinyinhua, *Lonicera japonica* Thunb.).	Decoction
Wang (2013)	Zishui Tongmai decoction	Milkvetch Root (Huangqi, Astragalus mongholicus Bunge) 30 g, Danshen Root (Danshen, Salvia miltiorrhiza Bunge) 15 g, Radix Angelicae Sinensis (Danggui, Angelica sinensis (Oliv.) Diels) 15 g, Fructus Mori (Sangshen, Morus alba L.) 15 g, Earthworm (Dilong, Pheretima) 9g, Tea Tree Root (Chashugen, Camellia sinensis L.) Kuntze) 10 g, Grassleaf Sweetflag Rhizome (Shichangpu, Acorus gramineus Aiton) 9 g, Oriental Water Plantain Rhizome (Zexie, Alisma plantago-aquatica L.) 9 g, Thomson Kudzuvine Root (Gegen, Pueraria montana var. thomsonii (Benth.) M.R.Almeida) 12 g and Common Anemarrhena Rhizome (Zhimu, Anemarrhena asphodeloides Bunge) 12 g	Decoction
Yang (2013)	Bushen Yiqi Huoxue decoction	Rehmannia Root (Dihuang, Rehmannia glutinosa (Gaertn.) DC.) 30 g, Ginseng (Renshen, Panax ginseng C.A.Mey.) 10g, Milkvetch Root (Huangqi, Astragalus mongholicus Bunge) 30 g, Asiatic Cornelian Cherry Fruit (Shanzhuyu, Cornus officinalis Siebold & Zucc.) 15 g, Common Yam Rhizome (Shanyao, Dioscorea oppositifolia L.) 30 g, Dodder Seed (Tusizi, Cuscuta chinensis Lam.) 15 g, Danshen Root (Danshen, Salvia miltiorrhiza Bunge) 18 g, Safflower (Honghua, Carthamus tinctorius L.) 12 g, Thomson Kudzuvine Root (Gegen, Pueraria montana var. thomsonii (Benth.) M.R.Almeida) 30 g and Chinese Magnoliavine Fruit (Wuweizi, Schisandra chinensis (Turcz.) Baill.) 10 g	Decoction
Zuo et al. (2013)	Jinchai Xiaoke capsule	Chinese Thorowax Root (Chaihu, Bupleurum falcatum L.), White Paeony Root (Baishao, Paeonia lactiflora Pall.), Turmeric Root Tuber (Yujin, Curcuma longa L.), Lychee Seed (Lizhihe, Litchi chinensis Sonn.) and Rehmannia Root (Dihuang, Rehmannia glutinosa (Gaertn.) DC.).	Capsule
Fu (2014)	Luomaitong granule	Chinese Taxillus Herb (Sangjisheng, Taxillus chinensis (DC.) Danser), Fleeceflower Root (Heshouwu, Reynoutria multiflora (Thunb.) Moldenke), Asiatic Cornelian Cherry Fruit (Shanzhuyu, Cornus officinalis Siebold & Zucc.), Ramulus Euonymi Alati (Guijianyu, Euonymus alatus (Thunb.) Sieb.), Turmeric (Jianghuang, Curcuma longa L.), Leech (Shuizhi, Hirudo), Stiff Silkworm (Jiangcan, Batryticatus Bombyx), Danshen Root (Danshen, Salvia miltiorrhiza Bunge) and Pseudo-ginseng (Sanqi, Panax notoginseng (Burkill) F.H.Chen)	Granule
Li et al. (2014)	Tongxinluo capsule	Ginseng (Renshen, Panax ginseng C.A.Mey.), Leech (Shuizhi, Hirudo), Scorpion (Quanxie, Scorpio), Red Peony Root (Chishao, Paeonia lactiflora Pall.), Cicada Slough (Chantui, Cicadae Periostracum), Ground Beetle (Tubiechong, Eupolyphaga Seu Steleophaga), Centipede (Wugong, Scolopendra), Sandalwood (Tanxiang, Santalum album L.), Rosewood (Jiangxiang, Dalbergia odorifera T.C.Chen), Frankincense (Ruxiang, Boswellia sacra Flück.), Spine Date Seed (Suanzaoren, Ziziphus jujuba Mill.) and Borneol (Bingpian, Dryobalanops aromatica Gaertn.f.).	Capsule
Sun et al. (2015)	Tongxinluo capsule	Ginseng (Renshen, Panax ginseng C.A.Mey.), Leech (Shuizhi, Hirudo), Scorpion (Quanxie, Scorpio), Red Peony Root (Chishao, Paeonia lactiflora Pall.), Cicada Slough (Chantui, Cicadae Periostracum), Ground Beetle (Tubiechong, Eupolyphaga Seu Steleophaga), Centipede (Wugong, Scolopendra), Sandalwood (Tanxiang, Santalum album L.), Rosewood (Jiangxiang, Dalbergia odorifera T.C.Chen), Frankincense (Ruxiang, Boswellia sacra Flück.), Spine Date Seed (Suanzaoren, Ziziphus jujuba Mill.) and Borneol (Bingpian, Dryobalanops aromatica Gaertn.f.).	Capsule
Luo (2015)	Danzhi Jiangtang capsule	Heterophylly Falsestarwort Root (Taizishen, Pseudostellaria heterophylla (Miq.) Pax), Rehmannia Root (Dihuang, Rehmannia glutinosa (Gaertn.) DC.), Tree Peony Bark (Mudanpi, Paeonia × suffruticosa Andrews), Dodder Seed (Tusizi, Cuscuta chinensis Lam.), Oriental Water Plantain Rhizome (Zexie, Alisma plantago-aquatica L.) and Leech (Shuizhi, Hirudo)	Capsule
Cong (2016)	Jianpi Tongmai decoction	Milkvetch Root (Huangqi, Astragalus mongholicus Bunge), Solomonseal Rhizome (Huangjing, Vitex negundo L.), Common Yam Rhizome (Shanyao, Dioscorea oppositifolia L.), Dendrobium (Shihu, Dendrobium nobile Lindl.), Dried Tangerine Peel (Chenpi, Citrus × aurantium L.), Pinellia Tuber (Banxia, Pinellia ternata (Thunb.) Makino), Oriental Water Plantain Rhizome (Zexie, Alisma plantago-aquatica L.), Thomson Kudzuvine Root (Gegen, Pueraria montana var. thomsonii (Benth.) M.R.Almeida), Villous Amomum Fruit (Sharen, Wurfbainia villosa (Lour.) Skornick. & A.D.Poulsen) and Pseudo-ginseng (Sanqi, Panax notoginseng (Burkill) F.H.Chen)	Decoction
Lu et al. (2016)	Modified Simiao Yongan decoction	Honeysuckle Flower (Jinyinhua, *Lonicera japonica* Thunb.) 30 g, Figwort Root (Xuanshen, Scrophularia ningpoensis Hemsl.) 9 g, Radix Angelicae Sinensis (Danggui, Angelica sinensis (Oliv.) Diels) 15 g and Liquorice Root (Gancao, Glycyrrhiza glabra L.) 6 g. If qi deficiency was identified, Milkvetch Root (Huangqi, Astragalus mongholicus Bunge) 15 g and Tangshen (Dangshen, Codonopsis pilosula (Franch.) Nannf.) 15 g were added. If phlegm turbidity was identified, Dried Tangerine Peel (Chenpi, Citrus × aurantium L.) 6 g, Indian Bread (Fuling, Poria cocos (Schw.) Wolf.) 12 g and Grassleaf Sweetflag Rhizome (Shichangpu, Acorus gramineus Aiton) 12 g were added. If qi stagnation was identified, Nutgrass Galingale Rhizome (Xiangfu, Cyperus rotundus L.) 12 g and Rhizoma Corydalis (Yanhusuo, Corydalis yanhusuo (Y.H.Chou & Chun C.Hsu) W.T.Wang ex Z.Y.Su & C.Y.Wu) 15 g were added. If yin deficiency was identified, Solomonseal Rhizome (Huangjing, Vitex negundo L.) 15 g and Rehmannia Root (Dihuang, Rehmannia glutinosa (Gaertn.) DC.) 12 g were added. If blood stasis was identified, Szechwan Lovage Rhizome (Chuanxiong, Ligusticum striatum DC.) 12g, Red Peony Root (Chishao, Paeonia lactiflora Pall.) 30 g and Danshen Root (Danshen, Salvia miltiorrhiza Bunge) 30 g were added	Decoction
Lyu et al. (2016)	Tongxinluo capsule	Ginseng (Renshen, Panax ginseng C.A.Mey.), Leech (Shuizhi, Hirudo), Scorpion (Quanxie, Scorpio), Red Peony Root (Chishao, Paeonia lactiflora Pall.), Cicada Slough (Chantui, Cicadae Periostracum), Ground Beetle (Tubiechong, Eupolyphaga Seu Steleophaga), Centipede (Wugong, Scolopendra), Sandalwood (Tanxiang, Santalum album L.), Rosewood (Jiangxiang, Dalbergia odorifera T.C.Chen), Frankincense (Ruxiang, Boswellia sacra Flück.), Spine Date Seed (Suanzaoren, Ziziphus jujuba Mill.) and Borneol (Bingpian, Dryobalanops aromatica Gaertn.f.).	Capsule
Shi and Lu (2016)	Danzhi Jiangtang capsule	Heterophylly Falsestarwort Root (Taizishen, Pseudostellaria heterophylla (Miq.) Pax), Rehmannia Root (Dihuang, Rehmannia glutinosa (Gaertn.) DC.), Tree Peony Bark (Mudanpi, Paeonia × suffruticosa Andrews), Dodder Seed (Tusizi, Cuscuta chinensis Lam.), Oriental Water Plantain Rhizome (Zexie, Alisma plantago-aquatica L.) and Leech (Shuizhi, Hirudo)	Capsule
Xu (2016)	Jianpi Xiaozhi decoction	Heterophylly Falsestarwort Root (Taizishen, Pseudostellaria heterophylla (Miq.) Pax) 12 g, Barbary Wolfberry Fruit (Gouqizi, Lycium barbarum L.) 15 g, Pinellia Tuber (Banxia, Pinellia ternata (Thunb.) Makino) 12 g, Bamboo Shavings (Zhuru, Bambusa tuldoides Munro) 12 g, Safflower (Honghua, Carthamus tinctorius L.) 9g, Earthworm (Dilong, Pheretima) 9 g, Szechwan Lovage Rhizome (Chuanxiong, Ligusticum striatum DC.) 9g, Dried Tangerine Peel (Chenpi, Citrus × aurantium L.) 6g, Largehead Atractylodes Rh (Baizhu, Atractylodes macrocephala Koidz.) 20 g, Cassia Seed (Juemingzi, Senna tora (L.) Roxb.) 9 g and Medicated Leaven (Shenqu, Massa Medicata Fermentata) 9 g	Decoction
Yang et al. (2016b)	Hawthorn formula granule	Hawthorn Fruit (Shanzha, Crataegus monogyna Jacq).	Granule
Chen et al. (2017)	Yishen Huoxue Huatan decoction	Rehmannia Root (Dihuang, Rehmannia glutinosa (Gaertn.) DC.) 20 g, Barbary Wolfberry Fruit (Gouqizi, Lycium barbarum L.) 15 g, Asiatic Cornelian Cherry Fruit (Shanzhuyu, Cornus officinalis Siebold & Zucc.) 12 g, Dodder Seed (Tusizi, Cuscuta chinensis Lam.) 12 g, Common Yam Rhizome (Shanyao, Dioscorea oppositifolia L.) 12 g, Red Peony Root (Chishao, Paeonia lactiflora Pall.) 15 g, Twotoothed Achyranthes Root (Niuxi, Achyranthes bidentata Blume) 15 g, Tree Peony Bark (Mudanpi, Paeonia × suffruticosa Andrews) 9g, Dried Tangerine Peel (Chenpi, Citrus × aurantium L.) 6 g and Pinellia Tuber (Banxia, Pinellia ternata (Thunb.) Makino) 9 g	Decoction
Yang (2017)	Jiawei Zicui Tongmai decoction	Rehmannia Root (Dihuang, Rehmannia glutinosa (Gaertn.) DC.) 20 g, Asiatic Cornelian Cherry Fruit (Shanzhuyu, Cornus officinalis Siebold & Zucc.) 15 g, Solomonseal Rhizome (Huangjing, Vitex negundo L.) 20 g, Milkvetch Root (Huangqi, Astragalus mongholicus Bunge) 30 g, Common Yam Rhizome (Shanyao, Dioscorea oppositifolia L.) 20g, Snakegourd Fruit (Gualou, Trichosanthes kirilowii Maxim.) 10 g, Stiff Silkworm (Jiangcan, Batryticatus Bombyx) 10 g, Danshen Root (Danshen, Salvia miltiorrhiza Bunge) 15 g, Ramulus Euonymi Alati (Guijianyu, Euonymus alatus (Thunb.) Sieb.) 10g, Cattail Pollen (Puhuang, Typha angustifolia L.) 8g, Earthworm (Dilong, Pheretima) 6g, Rhizoma Coptidis (Huanglian, Coptis chinensis Franch.) 6 g and Liquorice Root (Gancao, Glycyrrhiza glabra L.) 6 g	Decoction
Bu et al. (2018)	Modified Taohong Siwu decoction	Peach Seed (Taoren, Prunus persica L.) Batsch) 15g, Rehmannia Root (Dihuang, Rehmannia glutinosa (Gaertn.) DC.) 15 g, Danshen Root (Danshen, Salvia miltiorrhiza Bunge) 15g, Radix Angelicae Sinensis (Danggui, Angelica sinensis (Oliv.) Diels) 10 g, Red Peony Root (Chishao, Paeonia lactiflora Pall.) 10g, Szechwan Lovage Rhizome (Chuanxiong, Ligusticum striatum DC.) 10 g, Safflower (Honghua, Carthamus tinctorius L.) 10 g, Tree Peony Bark (Mudanpi, Paeonia × suffruticosa Andrews) 10 g and Immature Orange Fruit (Zhishi, Citrus aurantium L.) 10 g	Decoction
Fan et al. (2020)	Tongmai Yuban pill	Milkvetch Root (Huangqi, Astragalus mongholicus Bunge) 60g, Szechwan Lovage Rhizome (Chuanxiong, Ligusticum striatum DC.) 15g, Red Peony Root (Chishao, Paeonia lactiflora Pall.) 30g, Danshen Root (Danshen, Salvia miltiorrhiza Bunge) 30 g, Snakegourd Fruit (Gualou, Trichosanthes kirilowii Maxim.) 30 g, Longstamen Onion Bulb (Xiebai, Allium chinense G.Don) 15g, Pinellia Tuber (Banxia, Pinellia ternata (Thunb.) Makino) 15g, Radix Angelicae Sinensis (Danggui, Angelica sinensis (Oliv.) Diels) 30g, Safflower (Honghua, Carthamus tinctorius L.) 10g, Turmeric Root Tuber (Yujin, Curcuma longa L.) 10g, Hawthorn Fruit (Shanzha, Crataegus monogyna Jacq.) 30g, Leech (Shuizhi, Hirudo) 15g, Earthworm (Dilong, Pheretima) 20g, Giant Knotweed Rhizome (Huzhang, Reynoutria japonica Houtt.) 30 g and Ramulus Euonymi Alati (Guijianyu, Euonymus alatus (Thunb.) Sieb.) 15 g	Pill
Guo (2020)	Notoginseng powder	Pseudo-ginseng (Sanqi, Panax notoginseng (Burkill) F.H.Chen)	Powder
Mao (2020)	Yudan Shengui capsule	Ginseng (Renshen, Panax ginseng C.A.Mey.), Szechwan Lovage Rhizome (Chuanxiong, Ligusticum striatum DC.) and Cassia Twig (Guizhi, Neolitsea cassia L.) Kosterm.).	Capsule
Jiao et al. (2021)	Modified Yuye decoction	Common Yam Rhizome (Shanyao, Dioscorea oppositifolia L.) 20g, Heterophylly Falsestarwort Root (Taizishen, Pseudostellaria heterophylla (Miq.) Pax) 20g, Danshen Root (Danshen, Salvia miltiorrhiza Bunge) 20g, Snakegourd Root (Tianhuafen, Trichosanthes kirilowii Maxim.) 20g, Milkvetch Root (Huangqi, Astragalus mongholicus Bunge) 30g, Common Anemarrhena Rhizome (Zhimu, Anemarrhena asphodeloides Bunge) 10g, Thomson Kudzuvine Root (Gegen, Pueraria montana var. thomsonii (Benth.) M.R.Almeida) 10g, Chinese Magnoliavine Fruit (Wuweizi, Schisandra chinensis (Turcz.) Baill.) 10g, Chickens Gizzard-membrane (Jineijin, Gallus *gallus domesticus* Brisson) 10g, Szechwan Lovage Rhizome (Chuanxiong, Ligusticum striatum DC.) 10g, Dried Tangerine Peel (Chenpi, Citrus × aurantium L.) 10g, Red Rice (Hongqu, Monascus purpureus Went.) 5g, Liquorice Root (Gancao, Glycyrrhiza glabra L.) 5 g and Pseudo-ginseng (Sanqi, Panax notoginseng (Burkill) F.H.Chen) 3 g. If vexing heat in the chest, palms and soles was identified, Dwarf Lilyturf Tuber (Maidong, Ophiopogon japonicus (Thunb.) Ker Gawl.) 10 g and Rehmannia Root (Dihuang, Rehmannia glutinosa (Gaertn.) DC.) 10 g were added. If phlegm turbidity obstructing the spleen was identified, Rhizoma Atractylodis (Cangzhu, Atractylodes lancea (Thunb.) DC.) 10 g and Largehead Atractylodes Rh (Baizhu, Atractylodes macrocephala Koidz.) 10 g were added. If chest oppression and stabbing pain were identified, Safflower (Honghua, Carthamus tinctorius L.) 10 g and Earthworm (Dilong, Pheretima) 10 g were added. If palpitations and insomnia were identified, Chinese Arborvitae Kernel (Baiziren, Platycladus orientalis L.) Franco) 10 g and Spine Date Seed (Suanzaoren, Ziziphus jujuba Mill.) 10 g were added. If qi-deficiency sweatiness was identified, the doses of Heterophylly Falsestarwort Root (Taizishen, Pseudostellaria heterophylla (Miq.) Pax) and Milkvetch Root (Huangqi, Astragalus mongholicus Bunge) were increased. If tidal fever and night sweating were identified, Drgonsbones (Longgu, Apatite) 10 g and Oyster Shell (Shengmuli, Ostreae Concha) 10 g were added. If frequent urination was identified, Asiatic Cornelian Cherry Fruit (Shanzhuyu, Cornus officinalis Siebold & Zucc.) 10 g and Rehmannia Root (Dihuang, Rehmannia glutinosa (Gaertn.) DC.) 10 g were added	Decoction
Wang et al. (2021c)	Furong Tongmai decoction	Prepared Common Monkshood Daughter Root (Fuzi, Raphanus raphanistrum subsp. sativus L.) Domin) 7g, Leech (Shuizhi, Hirudo) 10g, Earthworm (Dilong, Pheretima) 15g, Scorpion (Quanxie, Scorpio) 3g, Milkvetch Root (Huangqi, Astragalus mongholicus Bunge) 20g, Radix Angelicae Sinensis (Danggui, Angelica sinensis (Oliv.) Diels) 15g, Figwort Root (Xuanshen, Scrophularia ningpoensis Hemsl.) 20g, Thomson Kudzuvine Root (Gegen, Pueraria montana var. thomsonii (Benth.) M.R.Almeida) 20g, Tuber Fleeceflower Stem (Shouwuteng, Reynoutria multiflora (Thunb.) Moldenke) 20g, Twotoothed Achyranthes Root (Niuxi, Achyranthes bidentata Blume) 15g, Chuanlong Yam (Chuanshanlong, Dioscorea nipponica Makino) 15 g and Liquorice Root (Gancao, Glycyrrhiza glabra L.) 10 g. If insomnia was identified, Thinleaf Milkwort Root (Yuanzhi, Polygala tenuifolia Willd.) 10g, Spine Date Seed (Suanzaoren, Ziziphus jujuba Mill.) 15 g and Silktree Albizia Bark (Hehuanpi, Albizia julibrissin Durazz.) 20 g were added. If fear of cold and cold limbs were identified, Cassia Twig (Guizhi, Neolitsea cassia L.) Kosterm.) 10 g was added. If poor blood pressure control was identified, Tall Gastrodia Tuber (Tianma, Gastrodia elata Blume) 10g, Gambir Plant (Gouteng, Uncaria rhynchophylla (Miq.) Miq.) 20 g and Abalone Shell (Shijueming, Haliotidis Concha) 15 g were added. If dizziness was identified, Tall Gastrodia Tuber (Tianma, Gastrodia elata Blume) 10 g and Chrysanthemum Flower (Juhua, Chrysanthemum × morifolium (Ramat.) Hemsl.) 15 g were added	Decoction

### Risk of bias assessment

**FIGURE 2:**
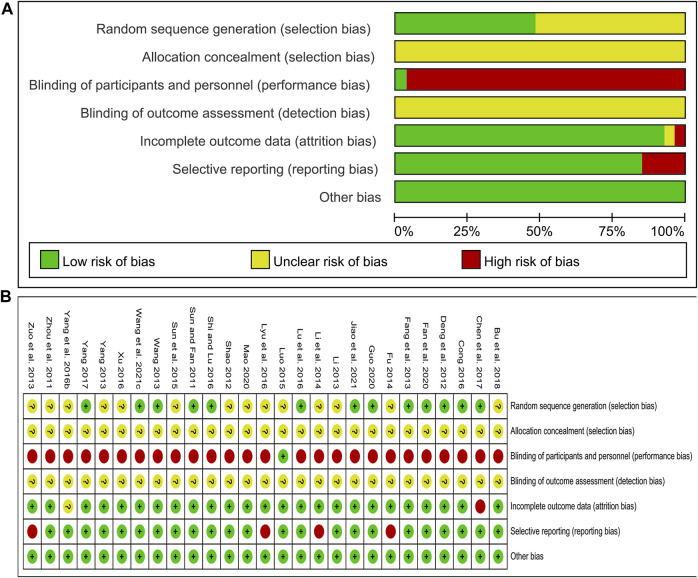
Risk of bias assessment for included studies: **(A)** Risk of bias graph; **(B)** Risk of bias summary.

The risk of bias was assessed using the Cochrane Risk of Bias tool. Of the included studies, 12 studies (Sun and Fan, 2011; Deng et al., 2012; Wang, 2013; Cong, 2016; Lu et al., 2016; Shi and Lu, 2016; Chen et al., 2017; Yang, 2017; Fan et al., 2020; Guo, 2020; Wang et al., 2021c; Jiao et al., 2021) used a random number table and 1 study (Fang et al., 2013) used a computer-generated random sequence, and these studies were marked as low risk. Other studies claimed to have performed randomization but did not report the specific methods used in the process, and these studies were marked as unclear risk. All studies did not report information about allocation concealment and were therefore marked as unclear risk. Only 1 study (Luo, 2015) blinded participants and researchers with an appropriate placebo and was assessed as low risk. In the remaining studies, the control group was treated with WM alone, and the treatment group was treated with CHM combined with WM. The number and dosage form of medications were different between the two groups, so the participants and researchers were not blinded, and these studies were evaluated as high risk. None of the studies stated whether outcome assessors were blinded and were marked as unclear risk. Concerning the outcome data completeness, 1 study (Chen et al., 2017) was rated as high risk due to the imbalance in the number and reasons of missing data between groups and the exclusion of missing data from the analysis. 1 study (Yang et al., 2016b) did not report the missing data and was rated as unclear risk. The remaining studies had no incomplete data. In terms of selective reporting, four studies (Zuo et al., 2013; Fu, 2014; Li et al., 2014; Lyu et al., 2016) did not report some indicators stated in the methods section, such as CIMT, glucose metabolism indicators, hemodynamic indicators or adverse events, and were therefore judged as high risk. All studies were marked as low risk for other biases because we did not find other important biases such as conflict of interest, baseline imbalance or pharmaceutical company funding. In general, the methodological quality of the included studies was not high. The risk of bias assessment results for included studies are shown in Figure 2.

### Primary Outcomes

#### CIMT

**FIGURE 3:**
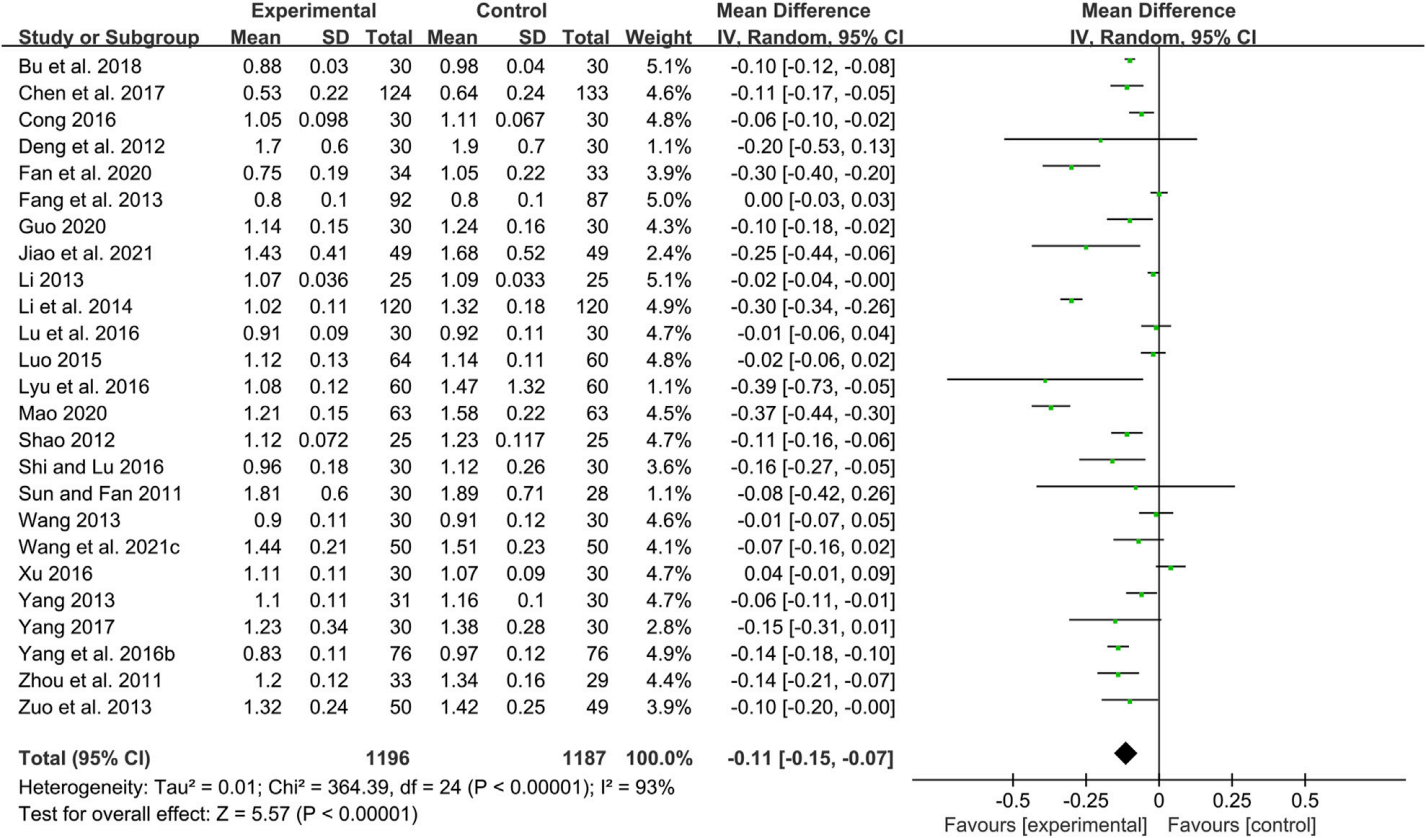
Forest plot of CIMT.

**FIGURE 4:**
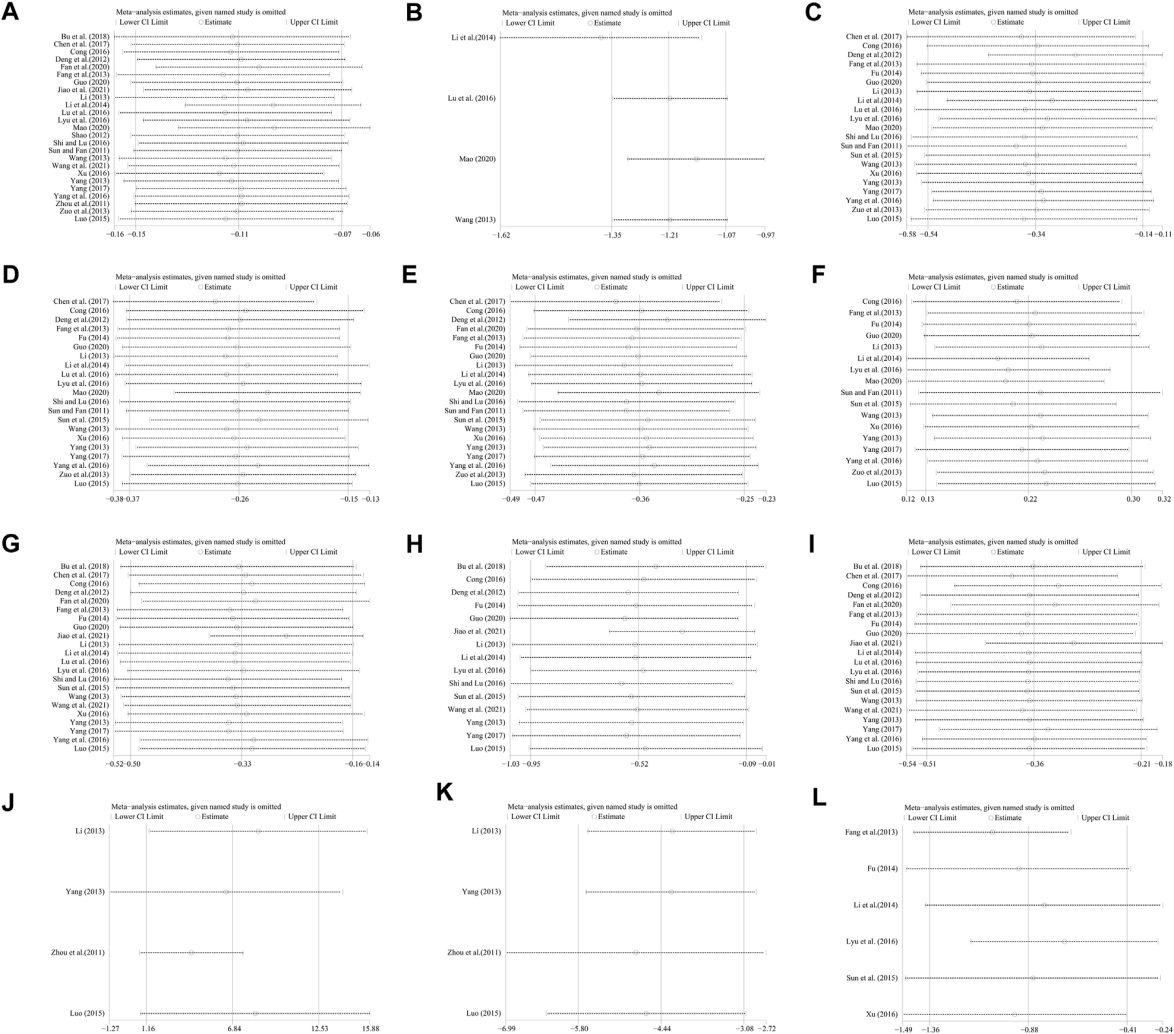
Sensitivity analysis: **(A)** CIMT; **(B)** Crouse score; **(C)** TC; **(D)** TG; **(E)** LDL-C; **(F)** HDL-C; **(G)** FBG; **(H)** 2hPG; **(I)** HbA1c; **(J)** NO; **(K)** ET-1; **(L)** HOMA-IR.

Twenty-five studies including 2383 patients compared CHM plus WM with WM (Sun and Fan, 2011; Zhou et al., 2011; Deng et al., 2012; Shao, 2012; Fang et al., 2013; Li, 2013; Wang, 2013; Yang, 2013; Zuo et al., 2013; Li et al., 2014; Luo, 2015; Yang et al., 2016b; Cong, 2016; Lu et al., 2016; Lyu et al., 2016; Shi and Lu, 2016; Xu, 2016; Chen et al., 2017; Yang, 2017; Bu et al., 2018; Fan et al., 2020; Guo, 2020; Mao, 2020; Wang et al., 2021c; Jiao et al., 2021). According to the heterogeneity test (*p* < 0.01, I^2^ = 93%), a random effect model was selected for statistical analysis. The pooled result showed that compared with WM, the combination with CHM could reduce the CIMT level, and the difference was statistically significant (MD = −0.11mm, 95%CI: −0.15 to −0.07, *p* < 0.01) (Figure 3). We performed meta-regression on average age, treatment duration and dosage form to identify possible sources of heterogeneity. The results showed that the dosage form might be one reason for the heterogeneity (*p* = 0.027, Adj R^2^ = 20.32%). We further analyzed the regression scatter plot and found that CHM in other dosage forms may have better effects on CIMT than in form of decoction (Supplementary Material S4). The reason may be that the efficacy of CHM in decoction is closely related to the standard of production process. There are strict requirements on the heat, time and water amount in the decoction process. Once the process does not meet the requirements, the efficacy may be affected. In contrast, the granule or capsule form is produced by qualified enterprises which process CHM in a specific method and concentrate them into standard doses. It could effectively control the factors which may result in changes in herb properties during storage, thus ensuring the quality and safety of CHM. At the same time, it is more convenient to take medicine, thus patients may have better compliance and the rate of medicine taking is guaranteed. We further performed a subgroup analysis on dosage form to explore which formulation is more effective for patients. The results showed that CHM may be more effective when taken in the form of pill (MD = −0.30 mm, 95%CI: −0.40 to −0.20, *p* < 0.01) or capsule (MD = −0.17 mm, 95%CI: −0.29 to −0.05, *p* < 0.01) than decoction (MD = −0.06 mm, 95%CI: −0.09 to −0.02, *p* < 0.01) (Supplementary Material S4). In addition, average age (*p* = 0.206, Adj R^2^ = 3.35%) and treatment duration (*p* = 0.618, Adj R^2^ = −3.84%) were not the sources of heterogeneity for CIMT (Supplementary Material S4). Sensitivity analysis showed that the pooled effect sizes were similar and the result was robust (Figure 4A; Supplementary Material S5).

#### Crouse score

**FIGURE 5:**
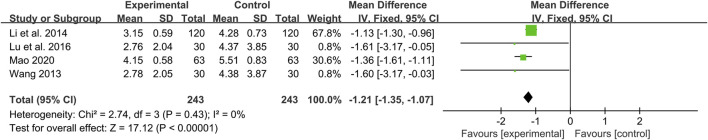
Forest plot of Crouse score.

Four studies including 486 patients evaluated the effectiveness of CHM plus WM with WM alone (Wang, 2013; Li et al., 2014; Lu et al., 2016; Mao, 2020). According to the heterogeneity test (*p* = 0.43, I^2^ = 0%), a fixed effect model was selected for statistical analysis. The result showed that combined with CHM could significantly reduce Crouse score, and the difference was statistically significant (MD = −1.21, 95%CI: −1.35 to −1.07, *p* < 0.01) (Figure 5). Subgroup analyses according to average age, treatment duration and dosage form were performed to explore the effect of these factors on the result. The results showed that combination treatment could reduce the Crouse score in T2DM patients with CAS of different average age, treatment duration and dosage form (Supplementary Material S6). Sensitivity analysis showed that the pooled effect sizes were similar and the result was relatively stable (Figure 4B, Supplementary Material S5).

### Secondary Outcomes

#### TC

**FIGURE 6:**
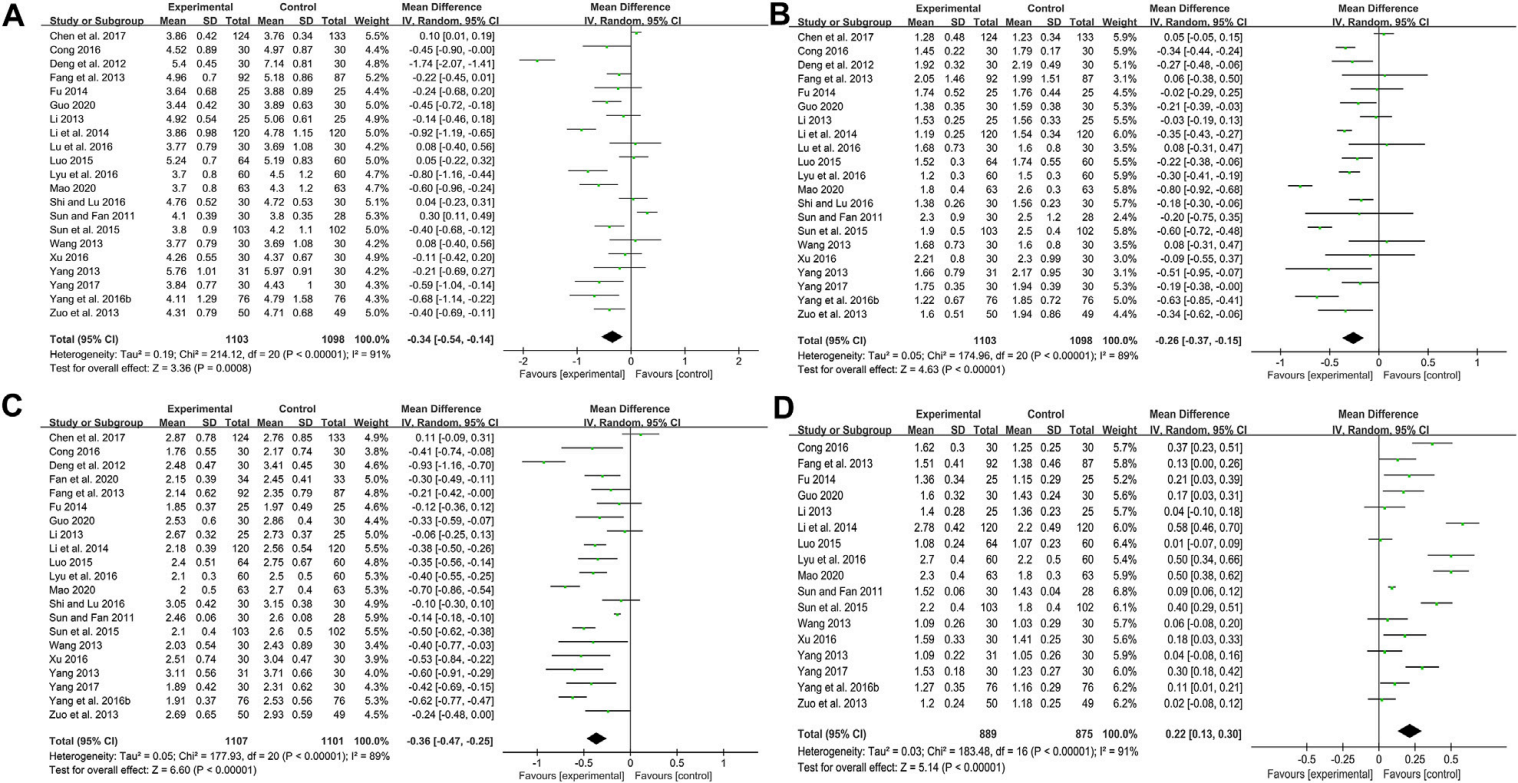
Forest plot of lipid metabolism index: **(A)** TC; **(B)** TG; **(C)** LDL-C; **(D)** HDL-C.

Twenty-one studies including 2201 patients compared CHM plus WM with WM (Sun and Fan, 2011; Deng et al., 2012; Fang et al., 2013; Li, 2013; Wang, 2013; Yang, 2013; Zuo et al., 2013; Fu, 2014; Li et al., 2014; Luo, 2015; Sun et al., 2015; Yang et al., 2016b; Cong, 2016; Lu et al., 2016; Lyu et al., 2016; Shi and Lu, 2016; Xu, 2016; Chen et al., 2017; Yang, 2017; Guo, 2020; Mao, 2020). According to the heterogeneity test (*p* < 0.01, I^2^ = 91%), a random effect model was selected for statistical analysis. The pooled result showed that when compared with WM, the combination with CHM could significantly reduce the TC level (MD = −0.34 mmol/L, 95%CI: −0.54 to −0.14, *p* < 0.01) (Figure 6A). Meta-regression was performed to explore possible sources of heterogeneity. In general, the average age (*p* = 0.922, Adj R^2^ = −5.70%), the treatment duration (*p* = 0.342, Adj R^2^ = −0.26%) and the dosage form (*p* = 0.466, Adj R^2^ = −2.42%) were not the sources of heterogeneity for the TC (Supplementary Material S4). Sensitivity analysis showed that the pooled effect sizes were similar and the result was robust (Figure 4C; Supplementary Material S5).

#### TG

Twenty-one studies including 2201 patients compared CHM plus WM with WM (Sun and Fan, 2011; Deng et al., 2012; Fang et al., 2013; Li, 2013; Wang, 2013; Yang, 2013; Zuo et al., 2013; Fu, 2014; Li et al., 2014; Luo, 2015; Sun et al., 2015; Yang et al., 2016b; Cong, 2016; Lu et al., 2016; Lyu et al., 2016; Shi and Lu, 2016; Xu, 2016; Chen et al., 2017; Yang, 2017; Guo, 2020; Mao, 2020). According to the heterogeneity test (*p* < 0.01, I^2^ = 89%), a random effect model was selected for statistical analysis. The pooled result showed that combined with WM might reduce TG in T2DM patients with CAS, and the difference was statistically significant (MD = −0.26 mmol/L, 95%CI: −0.37 to −0.15, *p* < 0.01) (Figure 6B). According to the meta-regression of average age, the scatters distribution showed a linear regularity, and the Tau^2^ decreased from 0.050 to 0.028, which suggested that age may be the source of heterogeneity and could explain 41.53% of the variation between studies (*p* = 0.006, Adj R^2^ = 41.53%) (Supplementary Material S4). We further analyzed the regression scatter plot and found that the efficacy on TG decreased with age, which means that the efficacy for young people may be better. We considered that the reason might be related to the simpler condition, fewer comorbidities and better response to herbs in young people. In addition, the treatment duration (*p* = 0.269, Adj R^2^ = 2.52%) and the dosage form (*p* = 0.052, Adj R^2^ = 20.13%) showed no significant difference on TG (Supplementary Material S4). Sensitivity analysis showed that the pooled effect sizes were similar and the result was robust (Figure4D; Supplementary Material S5).

#### LDL-C

Twenty-one studies including 2208 patients evaluated LDL-C levels (Sun and Fan, 2011; Deng et al., 2012; Fang et al., 2013; Li, 2013; Wang, 2013; Yang, 2013; Zuo et al., 2013; Fu, 2014; Li et al., 2014; Luo, 2015; Sun et al., 2015; Yang et al., 2016b; Cong, 2016; Lyu et al., 2016; Shi and Lu, 2016; Xu, 2016; Chen et al., 2017; Yang, 2017; Fan et al., 2020; Guo, 2020; Mao, 2020). According to the heterogeneity test (*p* < 0.01, I^2^ = 89%), a random effect model was selected for statistical analysis. The pooled result showed a significant lowering effect of CHM plus WM treatment on LDL-C (MD = −0.36 mmol/L, 95%CI: −0.47 to −0.25, *p* < 0.01) (Figure 6C). Meta-regression was performed to explore possible sources of heterogeneity. Altogether, the average age (*p* = 0.910, Adj R^2^ = −5.59%), the treatment duration (*p* = 0.098, Adj R^2^ = 9.76%) and the dosage form (*p* = 0.940, Adj R^2^ = −5.95%) were not the sources of heterogeneity for LDL-C (Supplementary Material S4). Sensitivity analysis showed that the pooled effect sizes were similar and the result was relatively stable (Figure 4E; Supplementary Material S5).

#### HDL-C

Seventeen studies including 1764 patients reported a comparison of HDL-C between combination treatment group and WM group (Sun and Fan, 2011; Fang et al., 2013; Li, 2013; Wang, 2013; Yang, 2013; Zuo et al., 2013; Fu, 2014; Li et al., 2014; Luo, 2015; Sun et al., 2015; Yang et al., 2016b; Cong, 2016; Lyu et al., 2016; Xu, 2016; Yang, 2017; Guo, 2020; Mao, 2020). According to the heterogeneity test (*p* < 0.01, I^2^ = 91%), a random effect model was selected for statistical analysis. The pooled result showed that compared with patients who only received WM treatment, the patients in the combination treatment group had higher HDL-C levels, and the difference was statistically significant (MD = 0.22 mmol/L, 95%CI: 0.13 to 0.30, *p* < 0.01) (Figure 6D). Meta-regression was performed to explore possible sources of heterogeneity. Meta-regression according to average age (*p* = 0.067, Adj R^2^ = 17.50%), the treatment duration (*p* = 0.217, Adj R^2^ = 4.43%) and the dosage form (*p* = 0.266, Adj R^2^ = 1.89%) showed no significant difference on HDL-C (Supplementary Material S4). Sensitivity analysis showed that the pooled effect sizes were similar and the result was relatively stable (Figure 4F; Supplementary Material S5).

#### FBG

**FIGURE 7:**
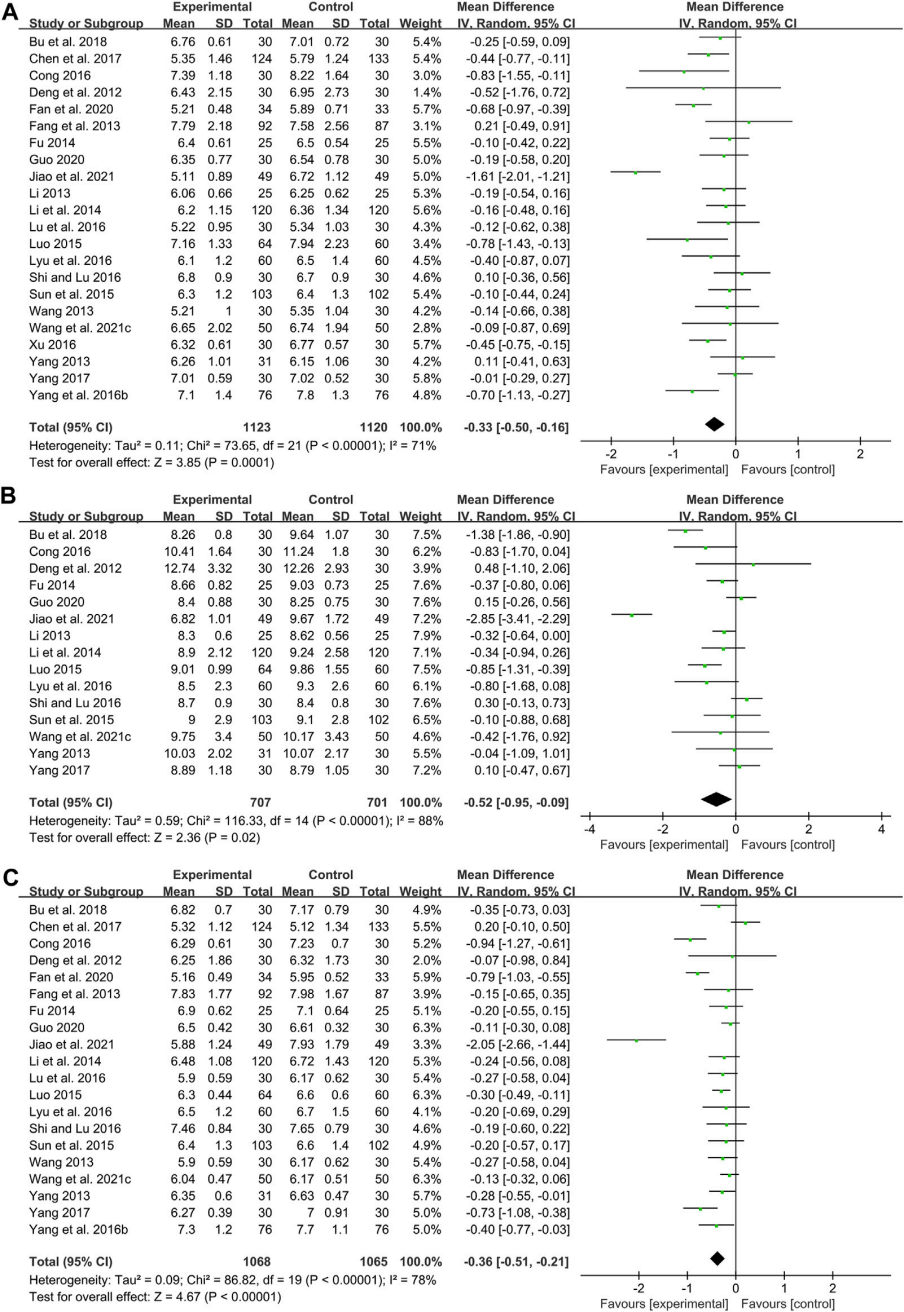
Forest plot of glucose metabolism index: **(A)** FBG; **(B)** 2hPG; **(C)** HbA1c.

Twenty-two studies including 2243 patients compared the FBG levels between combination treatment group and WM group (Deng et al., 2012; Fang et al., 2013; Li, 2013; Wang, 2013; Yang, 2013; Fu, 2014; Li et al., 2014; Luo, 2015; Sun et al., 2015; Yang et al., 2016b; Cong, 2016; Lu et al., 2016; Lyu et al., 2016; Shi and Lu, 2016; Xu, 2016; Chen et al., 2017; Yang, 2017; Bu et al., 2018; Fan et al., 2020; Guo, 2020; Wang et al., 2021c; Jiao et al., 2021). According to the heterogeneity test (*p* < 0.01, I^2^ = 71%), a random effect model was selected for statistical analysis. The pooled result illustrated that the combination treatment was remarkable for lowering FBG compared with WM (MD = −0.33 mmol/L, 95%CI: −0.50 to −0.16, *p* < 0.01) (Figure 7A). Meta-regression was performed to explore possible sources of heterogeneity. In general, the average age (*p* = 0.638, Adj R^2^ = −6.15%), the treatment duration (*p* = 0.992, Adj R^2^ = −7.23%) and the dosage form (*p* = 0.626, Adj R^2^ = −5.76%) were not significant sources of heterogeneity for FBG (Supplementary Material S4). Sensitivity analysis showed that the pooled effect sizes were similar and the result was robust (Figure 4G; Supplementary Material S5).

#### 2hPG

Fifteen studies including 1408 patients reported a comparison of 2hPG between combination treatment group and WM group (Deng et al., 2012; Li, 2013; Yang, 2013; Fu, 2014; Li et al., 2014; Luo, 2015; Sun et al., 2015; Cong, 2016; Lyu et al., 2016; Shi and Lu, 2016; Yang, 2017; Bu et al., 2018; Guo, 2020; Wang et al., 2021c; Jiao et al., 2021). According to the heterogeneity test (*p* < 0.01, I^2^ = 88%), a random effect model was selected for statistical analysis. The pooled result showed that compared with WM, combination with CHM could significantly reduce the 2hPG level (MD = −0.52 mmol/L, 95%CI: −0.95 to −0.09, *p* = 0.02) (Figure 7B). Meta-regression was performed to explore possible sources of heterogeneity. Meta-regression according to average age (*p* = 0.306, Adj R^2^ = 0.54%), the treatment duration (*p* = 0.561, Adj R^2^ = −4.08%) and the dosage form (*p* = 0.276, Adj R^2^ = 5.87%) showed no significant difference on 2hPG (Supplementary Material S4). Sensitivity analysis showed that the pooled effect sizes were similar and the result was relatively stable (Figure 4H; Supplementary Material S5).

#### HbA1c

Twenty studies including 2133 patients compared the HbA1c levels between combination treatment group and WM group (Deng et al., 2012; Fang et al., 2013; Wang, 2013; Yang, 2013; Fu, 2014; Li et al., 2014; Luo, 2015; Sun et al., 2015; Yang et al., 2016b; Cong, 2016; Lu et al., 2016; Lyu et al., 2016; Shi and Lu, 2016; Chen et al., 2017; Yang, 2017; Bu et al., 2018; Fan et al., 2020; Guo, 2020; Wang et al., 2021c; Jiao et al., 2021). According to the heterogeneity test (*p* < 0.01, I^2^ = 78%), a random effect model was selected for statistical analysis. The pooled result revealed that CHM plus WM was more effective in reducing HbA1c than WM, and the difference was statistically significant (MD = −0.36%, 95%CI: −0.51 to −0.21, *p* < 0.01) (Figure 7C). Meta-regression was performed to explore possible sources of heterogeneity. In general, the average age (*p* = 0.484, Adj R^2^ = −3.73%), the treatment duration (*p* = 0.835, Adj R^2^ = −10.52%) and the dosage form (*p* = 0.398, Adj R^2^ = −5.73%) were not the sources of heterogeneity for HbA1c (Supplementary Material S4). Sensitivity analysis showed that the pooled effect sizes were similar and the result was robust (Figure 4I; Supplementary Material S5).

#### NO

**FIGURE 8:**
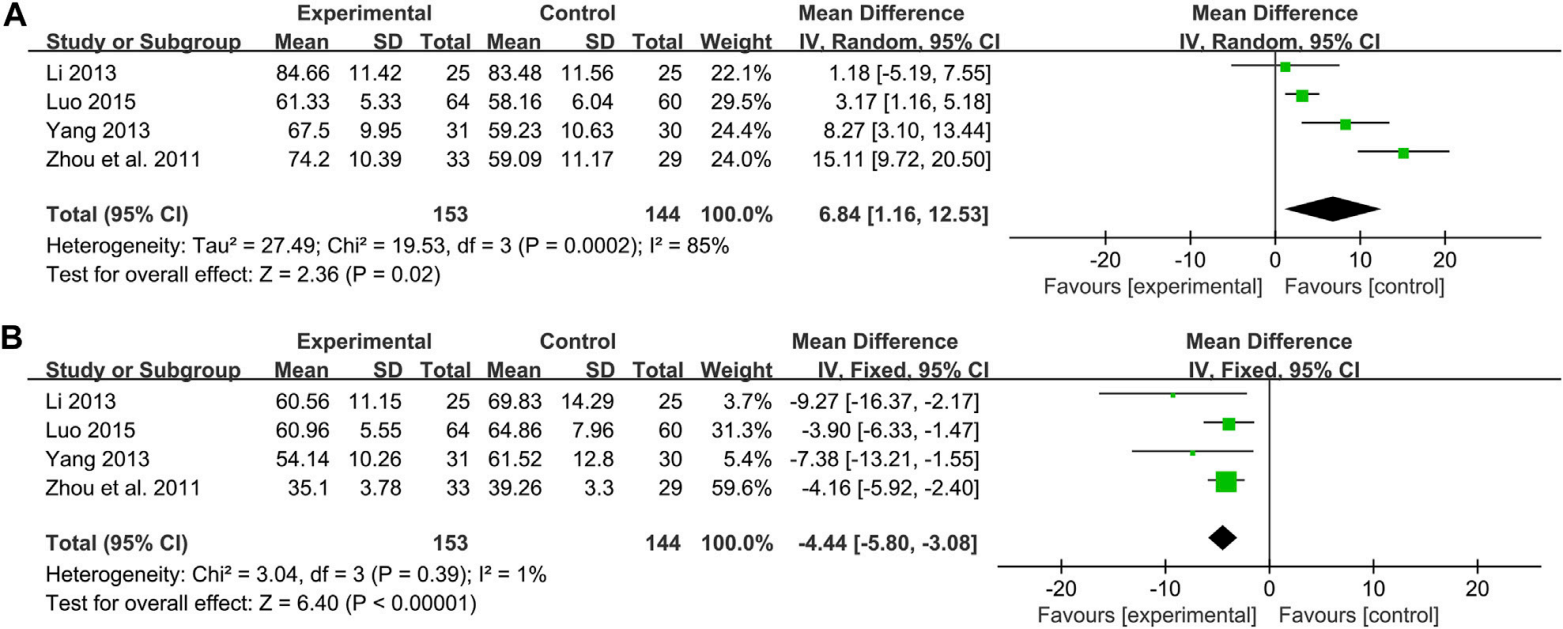
Forest plot of vascular endothelial function index: **(A)** NO; **(B)** ET-1.

Four studies including 297 patients evaluated the effectiveness of CHM plus WM to WM on NO (Zhou et al., 2011; Li, 2013; Yang, 2013; Luo, 2015). According to the heterogeneity test (*p* < 0.01, I^2^ = 85%), a random effect model was used. The pooled result found statistical significance between combination treatment and WM in NO (MD = 6.84 μmol/L, 95%CI: 1.16 to 12.53, *p* = 0.02) (Figure 8A). Due to the small number of included studies, there were only 1 or two articles within each subgroup, which may lead to poor representation. So, subgroup analysis to explore sources of heterogeneity cannot be performed well. We speculated that the reason for the heterogeneity might be related to the different disease stages of the patients between studies, resulting in different impairment degrees of vascular endothelial function. This is partly responsible for differences in baseline levels and drug responsiveness between study points. Sensitivity analysis showed that Yang, 2013 was highly sensitive. After excluding it, the pooled result was reversed and no statistical significance (MD = 6.42 μmol/L, 95%CI: −1.27 to 14.11, *p* = 0.10) (Figure 4J, Supplementary Material S5), indicating that the result is not robust. Yang, 2013 had a partial weight in the pooled result due to its moderate sample size and small standard deviation. Therefore, we are more convinced of the pooled effect size involved in this study, considering that the improvement of NO with combination treatment may be better than that of WM alone. However, due to the small number of included studies and limited sample size, this finding is not fully conclusive and further high-quality studies are needed to confirm it.

#### ET-1

Four studies including 297 patients compared the ET-1 levels between combination treatment group and the WM group (Zhou et al., 2011; Li, 2013; Yang, 2013; Luo, 2015). According to the heterogeneity test (*p* = 0.39, I^2^ = 1%), a fixed effect model was used. The pooled result revealed a significant decrease in ET-1 with combination treatment than with WM (MD = −4.44 pg/mL, 95%CI: −5.80 to −3.08, *p* < 0.01) (Figure 8B). Sensitivity analysis showed the result was robust (Figure 4K, Supplementary Material S5).

#### PSV

Two studies including 307 patients reported a comparison of PSV between combination treatment group and WM group (Fu, 2014; Chen et al., 2017). One study (Fu, 2014) including 50 patients reported that compared with WM alone, there were no significant differences on PSV in both left common carotid artery (MD = 4.28 cm/s, 95%CI: −0.45 to 9.01, *p* = 0.08) and right common carotid artery (MD = 1.61 cm/s, 95%CI: −4.07 to 7.30, *p* = 0.58) after 6 months combination treatment. However, another study (Chen et al., 2017) including 257 patients reported a significant improvement in mean PSV of bilateral common carotid arteries after 3 months combination treatment (MD = 5.91 cm/s, 95%CI: 2.53 to 9.29, *p* < 0.01). We speculated that the reason for the different study results may be related to the sample size. The former has a smaller sample size and may not have sufficient power to detect differences. Due to the small number of included studies, the efficacy of combination treatment on PSV is still unclear, and more high-quality and large-sample studies are still needed to explore it.

#### RI

Two studies including 307 patients evaluated RI outcomes (Fu, 2014; Chen et al., 2017). One study (Fu, 2014) including 50 patients reported that compared with WM, combined with CHM could reduce RI in both left common carotid artery (MD = −0.03, 95%CI: −0.06 to −0.00, *p* = 0.03) and right common carotid artery (MD = −0.02, 95%CI: −0.05 to −0.00, *p* = 0.04) after 6 months treatment, with a statistically significant difference. Another study (Chen et al., 2017), including 257 patients, similarly reported a significant reduction in mean RI of bilateral common carotid arteries after 3 months combination treatment (MD = −0.11, 95%CI: −0.12 to −0.10, *p* < 0.01). Due to the small sample size and large variation in results between studies, the efficacy of combination treatment on RI still needs more high-quality and large-sample studies to clarify.

#### HOMA-IR

**FIGURE 9:**
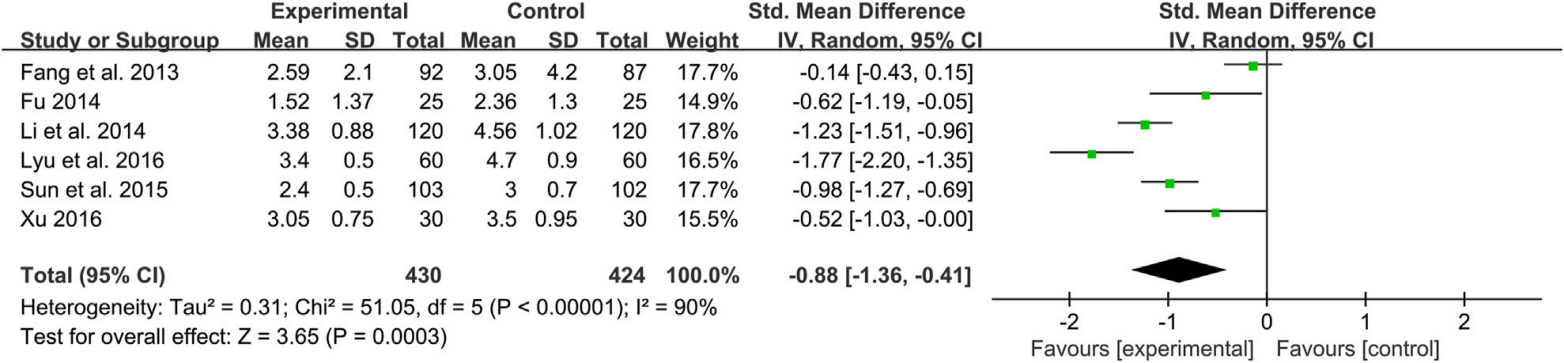
Forest plot of HOMA-IR.

Six studies including 854 patients reported a comparison of HOMA-IR between combination treatment group and WM group (Fang et al., 2013; Fu, 2014; Li et al., 2014; Sun et al., 2015; Lyu et al., 2016; Xu, 2016). According to the heterogeneity test (*p* < 0.01, I^2^ = 90%), a random effect model was used. The pooled result illustrated that combination with CHM was remarkable for reducing HOMA-IR compared with WM alone (SMD = −0.88, 95%CI: −1.36 to −0.41, *p* < 0.01) (Figure 9). Subgroup analyses according to average age, treatment duration and dosage form showed no significant reduction in heterogeneity within each subgroup, so these factors cannot be considered sources of heterogeneity at present (Supplementary Material S6). We speculated that the heterogeneity might be due to the large individual differences on HOMA-IR in T2DM patients with CAS, resulting in different HOMA-IR baseline levels and herb responsiveness between studies. It may also be related to measurement bias caused by different insulin detection methods. Sensitivity analysis showed that the pooled effect sizes were similar and the result was relatively stable (Figure 4L, Supplementary Material S5).

#### HOMA-β

Only 1 study including 205 patients evaluated HOMA-β outcomes. The result showed that compared with WM, the combination with CHM could improve HOMA-β, and the difference was statistically significant (MD = 0.80, 95%CI: 0.51 to 1.09, *p* < 0.01) (Sun et al., 2015).

### Adverse events

**FIGURE 10:**
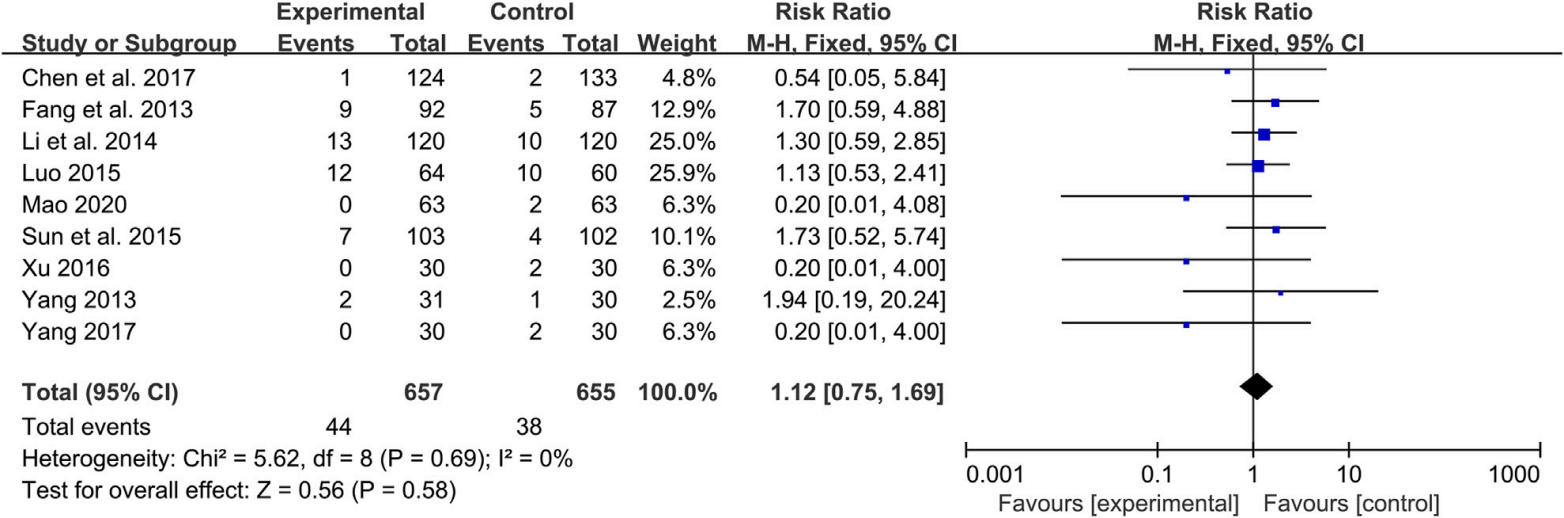
Forest plot of adverse events.

A total of 17 studies reported adverse events (Shao, 2012; Fang et al., 2013; Li, 2013; Wang, 2013; Yang, 2013; Fu, 2014; Li et al., 2014; Luo, 2015; Sun et al., 2015; Yang et al., 2016b; Cong, 2016; Xu, 2016; Chen et al., 2017; Yang, 2017; Fan et al., 2020; Guo, 2020; Mao, 2020). Among them, eight studies indicated that there were no adverse events occurred in both group during study period (Shao, 2012; Li, 2013; Wang, 2013; Fu, 2014; Yang et al., 2016b; Cong, 2016; Fan et al., 2020; Guo, 2020), and the remaining nine studies reported adverse events (Fang et al., 2013; Yang, 2013; Li et al., 2014; Luo, 2015; Sun et al., 2015; Xu, 2016; Chen et al., 2017; Yang, 2017; Mao, 2020). A total of 44/657 adverse events were reported in the experimental group and 38/655 in the control group. The meta-analysis showed that the incidence of adverse events in the combination treatment group was not significantly different from that in the control group (RR = 1.12, 95%CI: 0.75 to 1.69, *p* = 0.58) (Figure 10). As presented in Table 3, the most common adverse events were gastrointestinal reactions, including gastrointestinal discomfort, nausea, vomiting, diarrhea, abdominal distension and anorexia. Other adverse events such as abnormal liver function, dizziness, headache, skin itching, hypoglycemia, abnormal renal function and fatigue were also reported in these studies. These adverse reactions were all mild and can be relieved spontaneously or after symptomatic treatment. No serious adverse events or deaths were reported in the included studies. In general, CHM is relatively safe as an add-on treatment to Western medicine.

**TABLE 3:** Summary of adverse events.

	Number and severity reported in the treatment group	Number and severity reported in the control group
Gastrointestinal discomfort	22 mild	15 mild
Skin itching	5 mild	2 mild
Dizziness and headache	3 mild	5 mild
Abnormal liver function	3 mild	6 mild
Nausea and vomiting	3 mild	3 mild
Diarrhea	2 mild	1 mild
Abnormal renal function	2 mild	1 mild
Hypoglycemia reaction	2 mild	4 mild
Fatigue	1 mild	0
Abdominal distension	1 mild	0
Anorexia	0	1 mild
All adverse events reported in treatment or control group	44 mild	38 mild

### Publication bias

**FIGURE 11:**
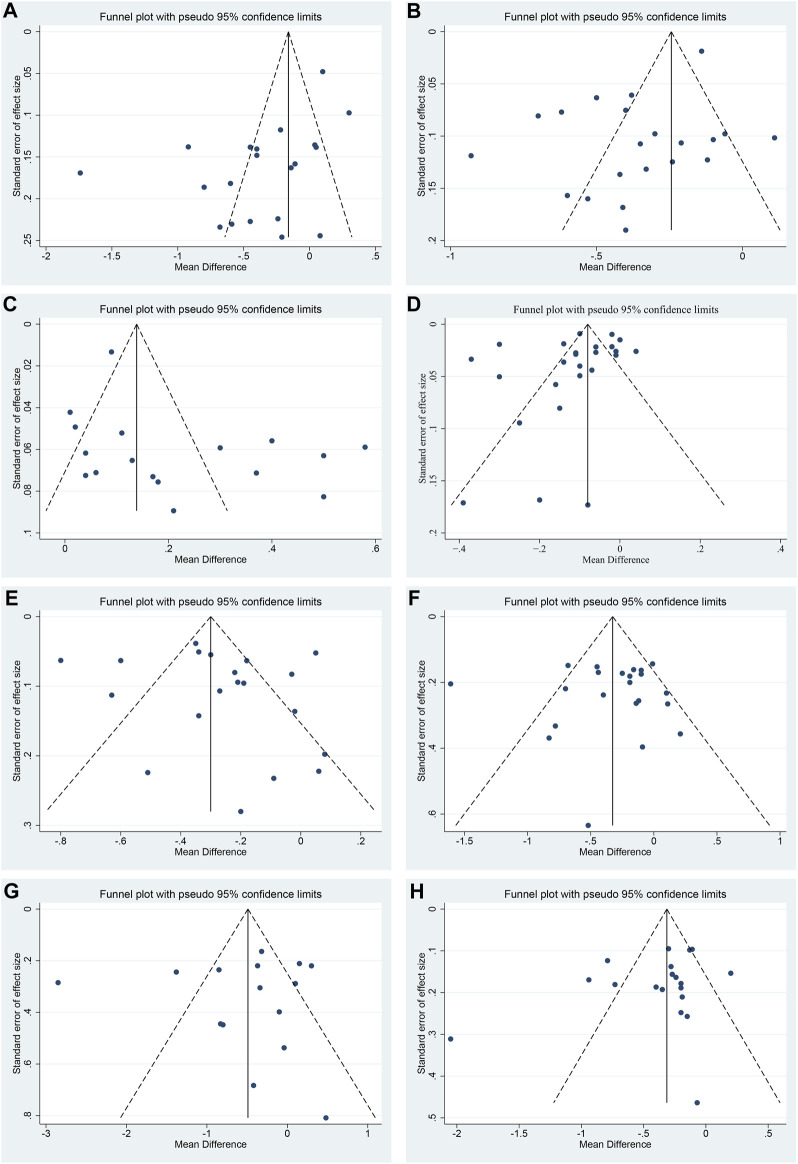
Funnel plots of **(A)** TC, **(B)** LDL-C, **(C)** HDL-C, **(D)** CIMT, **(E)** TG, **(F)** FBG, **(G)** 2hPG and **(H)** HbA1c.

Funnel plot and Egger’s test were performed to assess publication bias. For TC, LDL-C and HDL-C, the funnel plots showed left-right asymmetric distribution between study points (Figures 11A–C), and Egger’s test indicated possible publication bias (*p* = 0.011, 0.016 and 0.047, respectively) (Supplementary Material S7). For CIMT, TG, FBG, 2hPG and HbA1c, the funnel plots showed roughly inverted symmetrical distributions among study points (Figures 11D–H). Egger’s test showed no significant statistical difference (*p* = 0.190, 0.441, 0.910, 0.808 and 0.232, respectively) (Supplementary Material S7), suggesting there was no obvious publication bias.

### Assessment of evidence quality

The GRADE method was used to assess the evidence quality. The overall evidence quality for each outcome was moderate to very low. The decrease in the evidence certainty was mainly attributed to the high risk of bias, inconsistency between studies, imprecision in results and publication bias. Therefore, the results of this study should be applied with caution, and more high-quality studies are still needed to assess efficacy. Supplementary Material S8 showed a summary of the overall evidence for each outcome.

### Frequency distribution analysis of Chinese herb medicines

A total of 95 single CHMs were used in the included studies. We sorted the CHMs according to the occurrence frequency and listed the ones that appeared more than 5 times in Table 4. These CHMs could be further considered in the prescription for T2DM patients with CAS. The top four were Danshen Root (*Danshen*, Salvia miltiorrhiza Bunge), Milkvetch Root (*Huangqi*, Astragalus mongholicus Bunge), Rehmannia Root (*Dihuang*, Rehmannia glutinosa (Gaertn.) DC.) and Leech (*Shuizhi*, Hirudo).

**TABLE 4:** Frequency of CHMs (more than 5 times).

Chinese name	English name	Latin name	Number of studies (%)
Danshen	Danshen Root	Salvia miltiorrhiza Bunge	13 (48.15%)
Huangqi	Milkvetch Root	Astragalus mongholicus Bunge	13 (48.15%)
Dihuang	Rehmannia Root	Rehmannia glutinosa (Gaertn.) DC.	11 (40.74%)
Shuizhi	Leech	Hirudo	9 (33.33%)
Chishao	Red Peony Root	Paeonia lactiflora Pall	7 (25.93%)
Chuanxiong	Szechwan Lovage Rhizome	Ligusticum striatum DC.	7 (25.93%)
Dilong	Earthworm	Pheretima	7 (25.93%)
Gegen	Thomson Kudzuvine Root	Pueraria montana var. thomsonii (Benth.) M.R.Almeida	6 (22.22%)
Honghua	Safflower	Carthamus tinctorius L	6 (22.22%)
Zexie	Oriental Water Plantain Rhizome	Alisma plantago-aquatica L	6 (22.22%)
Chenpi	Dried Tangerine Peel	Citrus × aurantium L	5 (18.52%)
Danggui	Radix Angelicae Sinensis	Angelica sinensis (Oliv.) Diels	5 (18.52%)
Gancao	Liquorice Root	Glycyrrhiza glabra L	5 (18.52%)
Mudanpi	Tree Peony Bark	Paeonia × suffruticosa Andrews	5 (18.52%)
Quanxie	Scorpion	Scorpio	5 (18.52%)
Renshen	Ginseng	Panax ginseng C.A.Mey	5 (18.52%)
Sanqi	Pseudo-ginseng	Panax notoginseng (Burkill) F.H.Chen	5 (18.52%)
Shanyao	Common Yam Rhizome	Dioscorea oppositifolia L	5 (18.52%)
Shanzhuyu	Asiatic Cornelian Cherry Fruit	Cornus officinalis Siebold & Zucc	5 (18.52%)
Suanzaoren	Spine Date Seed	Ziziphus jujuba Mill	5 (18.52%)
Taizishen	Heterophylly Falsestarwort Root	Pseudostellaria heterophylla (Miq.) Pax	5 (18.52%)
Tusizi	Dodder Seed	Cuscuta chinensis Lam	5 (18.52%)
Shanzha	Hawthorn Fruit	Crataegus monogyna Jacq	5 (18.52%)

## Discussion

### Main results of this research

The occurrence and development of T2DM patients with CAS are closely related to various pathological factors such as glucose and lipid metabolism disorder, inflammatory response, oxidative stress, insulin resistance and vascular endothelial dysfunction (La Sala et al., 2019; Yuan et al., 2019; Poznyak et al., 2020). For T2DM patients with CAS, early intervention is of great significance in preventing cardiovascular and cerebrovascular events. CHM has played an important role in treating various diseases for its advantages in multi-component and multi-target synergistic regulation. Some clinical studies have shown that the application of CHM in the treatment of T2DM patients with CAS may achieve better efficacy.

Hence, we collected the latest evidence and updated a meta-analysis on the efficacy and safety of CHM in treating T2DM patients with CAS. We conducted a detailed and comprehensive search of Chinese and English databases and analyzed the effectiveness from multiple perspectives. Meta-regression and subgroup analysis were also performed to explore sources of heterogeneity, and GRADE was conducted to assess the evidence quality.

A total of 1096 articles were retrieved, and 27 studies including 2638 patients were included for analysis. The risk of bias assessment revealed that most studies needed to be concerned about the risk of bias, which was mainly related to the lack of detailed reporting on specific methods of random sequence generation and allocation concealment, and the absence of blinding.

Our main finding was that combined CHM with WM can significantly reduce the degree of carotid atherosclerosis in T2DM patients. Compared with WM alone, the combination with CHM could significantly reduce CIMT (MD = −0.11 mm, 95%CI: −0.15 to −0.07, *p* < 0.01), carotid plaque Crouse score (MD = −1.21, 95%CI: −1.35 to −1.07, *p* < 0.01), TC (MD = −0.34 mmol/L, 95%CI: −0.54 to −0.14, *p* < 0.01), TG (MD = −0.26 mmol/L, 95%CI: −0.37 to −0.15, *p* < 0.01), LDL-C (MD = −0.36 mmol/L, 95%CI: −0.47 to −0.25, *p* < 0.01) and improve HDL-C (MD = 0.22 mmol/L, 95%CI: 0.13 to 0.30, *p* < 0.01). Studies have shown that increased CIMT correlates well with ischemic stroke in the setting of T2DM (Kota et al., 2013). Each 0.1 mm increment in CIMT increased the risk of ischemic stroke by 17% (van den Oord et al., 2013). It can be seen that the combination treatment can achieve better efficacy in receding plaques and regulating blood lipids without increasing adverse effects, which means that CHM can be considered as a beneficial supplementary treatment for T2DM patients with CAS. In addition, we found that there was large heterogeneity in the results of CIMT, TC, TG, LDL-C and HDL-C, and meta-regression was used to explore the source of heterogeneity. The results revealed that the dosage form may be responsible for the heterogeneity of CIMT, and CHM may have a better efficacy on CIMT when it was not used in the form of decoction. This suggested that, in the future, in the long course of treatment, CHM can be considered made into granules, capsules or other convenient dosage forms to improve compliance and ensure the rate of medicine taking. Average age may be responsible for the heterogeneity of TG and CHM may be more effective in lowering TG in young people. Meta-regression did not find heterogeneity sources for TC, LDL and HDL. We speculated that, on the one hand, heterogeneity may be related to the uneven methodological quality of the included studies. On the other hand, CHM intervention’s specificity should also be considered. Syndrome differentiation and treatment is one of the characteristics of traditional Chinese medicine. For patients with different constitutions and pathogenesis, different CHMs will be selected to target the patient’s condition better, resulting in different herbal compositions and dosages for each formula. Moreover, different degrees of carotid atherosclerosis, such as carotid intima-media thickening, carotid atherosclerotic plaque or carotid stenosis, may respond differently to CHM treatment. The above factors may contribute to methodological as well as clinical heterogeneity, and further to statistical heterogeneity.

In terms of glucose metabolism, combined with CHM could reduce HbA1c (MD = −0.36%, 95%CI: −0.51 to −0.21, *p* < 0.01), FBG (MD = −0.33 mmol/L, 95%CI: −0.50 to −0.16, *p* < 0.01) and 2hPG (MD = −0.52 mmol/L, 95%CI: −0.95 to −0.09, *p* = 0.02) in T2DM patients with CAS. The improvement on HbA1c benefited from the decrease in both FBG and 2hPG. HbA1c can reflect the average blood glucose level over the last 2–3 months and is an important criterion for evaluating glycemic control. The United Kingdom Prospective Diabetes Study (UKPDS) suggested that the risk of various complications in diabetic patients is positively related to the level of glycemic control. Each 1% reduction in HbA1c reduced the risk of all diabetes-related endpoints by 21%, the risk of diabetes-related death by 21% and the risk of stroke by 12% (Stratton et al., 2000). Therefore, the reduction of blood glucose under the combination treatment will also benefit patients in preventing cardiovascular and cerebrovascular events. At the same time, we also found that the combination treatment was better than WM alone in reducing HOMA-IR (SMD = −0.88, 95%CI: −1.36 to −0.41, *p* < 0.01) and improving HOMA-β (MD = 0.80, 95%CI: 0.51 to 1.09, *p* < 0.01), and the difference was statistically significant.

In addition, we used the indexes about vascular endothelial cell function and vascular resistance to reflect the carotid artery condition in diabetic patients. Vascular endothelial dysfunction is an important pathological mechanism of T2DM patients with CAS (Gimbrone and García-Cardeña, 2016; Maruhashi and Higashi, 2021). NO and ET-1 are important markers reflecting vascular endothelial function, both produced by vascular endothelial cells. NO has the functions of dilating blood vessels, resisting platelet aggregation, limiting the proliferation of vascular smooth muscle, reducing vascular permeability and anti-inflammatory (Förstermann et al., 2017), while ET-1 could constrict blood vessels and promote vascular smooth muscle migration (Wang et al., 2022a). In the setting of diabetes, endothelial cell dysfunction leads to decreased synthesis and release of NO and excessive secretion of ET-1, resulting in pathological vasoconstriction, reduced diastolic function and smooth muscle cell proliferation (Rajendran et al., 2013), eventually vascular resistance increases. In this meta-analysis, NO, ET-1, PSV and RI were analyzed to explore the effects of CHM on vascular conditions. However, the efficacy remains uncertain due to the small number of included studies and large differences between study points. In the future, more high-quality and large-sample studies focusing on vascular conditions are still needed to explore the efficacy.

Of the 27 included studies, 17 reported adverse effects. Eight of them reported no adverse effects occurred during the study period. The remaining nine studies reported adverse reactions, most of which were gastrointestinal reactions, suggesting that clinicians should pay attention to the protection of gastrointestinal function while using CHM. Other adverse events, such as skin itching, dizziness, headache, abnormal liver function and hypoglycemia, were also reported. These adverse reactions were mild and resolved spontaneously or after symptomatic treatment. No serious adverse events or deaths occurred in these studies. The results showed that combination treatment did not increase the incidence of adverse events compared with WM (RR = 1.12, 95%CI: 0.75 to 1.69, *p* = 0.58), suggesting that combined with CHM was generally safe and tolerable.

From Table 4, it is worth noting that in the treatment of T2DM patients with CAS, some CHMs with the effect of activating blood and resolving stasis are often used, such as Danshen Root (*Danshen*, Salvia miltiorrhiza Bunge), Leech (*Shuizhi*, Hirudo), Red Peony Root (*Chishao*, Paeonia lactiflora Pall.), Szechwan Lovage Rhizome (*Chuanxiong*, Ligusticum striatum DC.), Earthworm (*Dilong*, Pheretima) and Safflower (*Honghua*, Carthamus tinctorius L.), which are consistent with the pathogenesis of blood stasis; however, there may be potential bleeding risks. None of the included studies monitored coagulation indexes during the trial, and none reported whether patients had bleeding symptoms. This suggests that when prescribing, the CHM should be selected reasonably and the dosage should be determined scientifically according to patient’s pathogenesis. Meanwhile, during treatment, close attention should be paid to the presence of bleeding, such as whether the patients have subcutaneous ecchymosis, petechiae, gingival bleeding or other suspicious bleeding symptoms. Blood routine, urine routine, stool routine and coagulation function should also be monitored. Once a possible bleeding condition is considered, the prescription should be stopped immediately and appropriate treatment should be carried out according to the severity of bleeding.

### Possible mechanism of Chinese herbal medicines for T2DM patients with CAS

The core Chinese herbal Medicines for T2DM patients with CAS are Danshen Root (*Danshen*, Salvia miltiorrhiza Bunge), Milkvetch Root (*Huangqi*, Astragalus mongholicus Bunge), Rehmannia Root (*Dihuang*, Rehmannia glutinosa (Gaertn.) DC.) and Leech (*Shuizhi*, Hirudo). Danshen Root (*Danshen*, Salvia miltiorrhiza Bunge), which is most frequently used, contains various chemical components such as tanshinones, salvianolic acids and lactones (Wan et al., 2020). Pharmacological studies have shown that tanshinone IIA has the functions of inhibiting inflammatory factors secretion (Li et al., 2015; Li et al., 2017), reducing adhesion molecules expression (Chang et al., 2014; Yang et al., 2016a), resisting vascular endothelial cells apoptosis (Zhu et al., 2017), inhibiting oxidative stress (Zhang et al., 2017a) and regulating lipid metabolism and immune function (Chen et al., 2019; Ye et al., 2022b), thereby protecting the function and structure of vascular endothelial and anti-atherosclerosis. Salvianolic acid A could reduce lipid deposition in vascular intima by inhibiting oxidative stress and inflammatory response and regulating lipid metabolism (Song et al., 2019). Salvianolic acid B also has the effects of relieving diabetes symptoms, regulating glucose and lipid metabolism, reducing hepatic gluconeogenesis gene expression and improving insulin resistance (Huang et al., 2015; Huang et al., 2016). The components of Milkvetch Root (*Huangqi*, Astragalus mongholicus Bunge) include polysaccharides, saponins, flavonoids, amino acids and various trace elements. Studies have shown that astragaloside IV could induce autophagy in macrophages by regulating the phosphatidylinositol 3-kinase/protein kinase B/mammalian target of rapamycin (PI3K/Akt/mTOR) signaling pathway, thereby inhibiting inflammatory responses and stabilizing atherosclerotic plaques. It can also promote cholesterol efflux from macrophages and reduce foam cell production (Zhang et al., 2017b; Qin et al., 2018; Ge et al., 2021). Astragalus polysaccharides could lower blood glucose, increase insulin sensitivity, improve insulin resistance and inhibit islet β-cell apoptosis (Zheng et al., 2020). The mechanism may be related to its potential to inhibit α-amylase activity, activate the AMP-activated protein kinase (AMPK) pathway, regulate endoplasmic reticulum stress signal and modulate apoptotic gene expression (Zhou, 2015; Wei et al., 2018; Zhang et al., 2018; Chen et al., 2020). Catalpol, one of the iridoid glycosides, is an important active ingredient of Rehmannia Root (*Dihuang*, Rehmannia glutinosa (Gaertn.) DC.) and an index component for controlling quality in Chinese Pharmacopoeia (ChPC, 2020; Wang et al., 2021b; Chen et al., 2021). Catalpol can lower blood glucose, reduce hepatic gluconeogenesis, increase glycogen synthesis and improve insulin resistance. It could also downregulate the expression of monocyte chemotactic protein-1 and vascular cell adhesion molecule-1, resist oxidative stress and inflammatory response and inhibit endothelial proliferation (Liu et al., 2016; Yan et al., 2018; Lin et al., 2019). This suggests that catalpol has a positive effect on diabetes and its associated vascular disease. The Leech (*Shuizhi*, Hirudo) is the dried whole body of the Whitmania pigra Whitman, Hirudo nipponica Whitman or Whitmania acranulata Whitman (ChPC, 2020). The Leech and its extracts can exert anti-atherosclerotic effects by regulating lipid metabolism, reducing oxidative stress, inhibiting inflammatory response and smooth muscle proliferation (Gao et al., 2014; Wu et al., 2017; Xu et al., 2019). All of the above CHMs exhibit the typical multi-target and multi-component regulation effects on T2DM patients with CAS.

### Agreement and disagreement with previous studies

Currently, there is a meta-analysis on this topic published a few years ago (Huang, 2015); however, it has serious methodological flaws. The protocol was not registered, which makes it less transparent and we cannot know if there was a deviation from the pre-study design. Meanwhile, the database search was not comprehensive and some important articles may be missed. The evaluation indicators were also incomplete; for example, lipid metabolism, the important parameter affecting atherosclerosis, was not assessed. Heterogeneity was not described and the evidence certainty could not be known. Seven years have passed since its publication, and whether its conclusions are applicable today needs to be considered. Our meta-analysis used a more rigorous methodology, carried out protocol registration in advance, conducted a comprehensive search of both Chinese and English databases and included newly published literature to ensure timeliness. Our updated systematic review included 27 RCTs, 14 of which were published after 2015. The efficacy was also evaluated from multiple perspectives to provide a comprehensive and reliable reference for subsequent studies. For indicators with heterogeneity, meta-regression or subgroup analysis was used to explore the source of heterogeneity, which was not available in the previous study. The previous study (Huang, 2015) found that combination treatment had better effects on CIMT and HbA1c in T2DM patients with CAS. Our study also found this, which is encouraging; however, due to the heterogeneity between studies, we think that high-quality studies are still needed to confirm this. Meanwhile, similar to the previous study, we found that there are still only a few studies evaluating vascular function. Given the important role of vascular function in T2DM patients with CAS, more studies are needed to explore the efficacy of CHM in this regard. More diverse indicators for evaluating vascular function can be considered, such as flow-mediated vasodilation (FMD), peripheral arterial tonometry (PAT) or pulse wave velocity (PWV).

### Limitation of this research

This study comprehensively collected clinical RCTs and provided the latest evidence for the efficacy and safety of CHM in treating T2DM patients with CAS. The results suggested that CHM may have great potential in treating this disease. However, this study still has some limitations. Firstly, the methodological quality of the included studies was not high. None of the studies registered trial protocols. Most studies had unclear randomization methods and a lack of blinding. Only one study used the placebo, and the remaining studies did not use double-blinding. These may affect the accuracy and reliability of the results. Because we could not get enough information from the authors, many items were assessed as uncertain risk in terms of risk of bias assessment. Secondly, some indicators have high heterogeneity. Although we have performed subgroup analysis or meta-regression, the source of heterogeneity cannot be completely determined. Heterogeneity may be due to various factors, such as the uneven study quality, the disease severity and the composition and dosage of CHM prescriptions. Thus, the results should be interpreted with caution. Thirdly, the most important purpose of treating T2DM patients with CAS is to prevent cardiovascular and cerebrovascular endpoint events. However, none of the studies conducted long-term follow-up observations and none of them reported endpoint events. Therefore, there is still a lack of evidence for CHM on long-term cardiovascular and cerebrovascular endpoints in T2DM patients with CAS. Fourthly, there are differences in the selection of evaluation indicators among included studies, and there is no uniform standard. Some parameters are less reported, such as NO, ET1, PSV and RI, and our exploration of the efficacy on these indicators is limited. Fifthly, some studies did not report adverse effects, which affected the evaluation of safety. Finally, although there is no restriction on publication language, all the articles that meet the criteria are in Chinese, and all of them are single-center and small-sample studies. The promotion and application of CHM are affected to a certain extent due to potential publication bias and possible ethnic and regional limitations.

### Implication for clinical practice and future research

Based on above findings and limitations, the following suggestions are provided for future research and practice. Firstly, improve study protocol rigor and strengthen quality control. Register the protocol in advance to ensure information transparency. Pay attention to the proper implementation of center randomization, allocation concealment and double-blinding to ensure study quality. Secondly, the design and reporting of RCTs should be conducted strictly following the Standard Protocol Items: Recommendations for Interventional Trials (SPIRIT) statement, the Consolidated Standards of Reporting Trials (CONSORT) statement and the CONSORT Extension for Chinese Herbal Medicine Formulas 2017. Study characteristics such as age, course of disease, disease severity, treatment duration and dosage should be clearly recorded for further analysis. Thirdly, long-term follow-up studies should be encouraged to clarify the efficacy of CHM on cardiovascular and cerebrovascular endpoint events in T2DM patients with CAS. Fourthly, discover the core outcome set about T2DM patients with CAS, especially the indicators reflecting the characteristics of traditional Chinese medicine. In clinical research, attention should be paid to the selection of evaluation indicators to make the use of indicators more standardized, representative and convincing. Fifthly, attach importance to observing and monitoring adverse events and establish a strict adverse events handling and reporting process. For T2DM patients with CAS, CHMs with the effect of activating blood and resolving stasis may be involved, which may have potential bleeding risks. Therefore, patients’ self-reported symptoms should be noted, and blood routine, urine routine, stool routine, liver and kidney function and coagulation indexes should also be monitored. Sixthly, calculate the sample size reasonably and conduct multi-center, double-blind and high-quality research to make the results more reliable. Both positive and negative results should be reported truthfully to reduce publication bias. Seventhly, Danshen Root (*Danshen*, Salvia miltiorrhiza Bunge), Milkvetch Root (*Huangqi*, Astragalus mongholicus Bunge), Rehmannia Root (*Dihuang*, Rehmannia glutinosa (Gaertn.) DC.) and Leech (*Shuizhi*, Hirudo) were the most frequently used CHMs in the included studies. These could be further studied as the core CHMs in treating T2DM patients with CAS. Finally, the material basis and mechanism of CHM for T2DM patients with CAS have not been clarified, and it should be revealed from the cellular, molecular and gene levels. Given the close relationship between T2DM and CAS, efforts to identify biomarkers and predictors of disease progression may help to identify patients at high risk of developing these conditions and allow for earlier and more effective intervention*.*

## Conclusion

The current evidence suggests that CHM treatment has great potential in reducing CIMT and carotid plaque Crouse score, regulating glucose and lipid metabolism, improving insulin resistance and enhancing islet β-cell function for T2DM patients with CAS. However, the current evidence is insufficient to clarify the effects of CHM on vascular conditions. Meanwhile, CHM is relatively safe. We have identified commonly used CHMs, which could be considered for further research and clinical application. Given the uneven methodological quality of the included studies, limited sample size and CHM heterogeneity, the results of this study should be interpreted and applied with caution. In the future, RCTs with larger samples and higher quality are still needed to provide more accurate and reliable information on the efficacy and safety of CHM in treating T2DM patients with CAS.

## Data Availability

The original contributions presented in the study are included in the article/Supplementary Material, further inquiries can be directed to the corresponding authors.

## Glossary

2hPG: 2-h postprandial glucose
AMPK: AMP-activated protein kinase
BMI: body mass index
CAS: carotid atherosclerosis
CBM: China Biomedical Medicine database
CG: control group interventions
CHiCTR: Chinese Clinical Trial Registry
CHM: Chinese herbal medicine
CI: confidence interval
CIMT: carotid intima-media thickness
CNKI: China National Knowledge Infrastructure
CONSORT: Consolidated Standards of Reporting Trials
ET-1: endothelin-1
FBG: fasting blood glucose
FMD: flow-mediated dilation
GLP-1: glucagon-like peptide-1
GRADE: Grading of Recommendations Assessment, Development and Evaluation
HbA1c: glycated hemoglobin
HDL-C: high-density lipoprotein cholesterol
HOMA-IR: homeostasis model assessment of insulin resistance
HOMA-β: homeostasis model assessment of beta-cell function
LDL-C: low-density lipoprotein cholesterol
MD: mean difference
NO: nitric oxide
NR: not reported
NSD: no significant difference
PAT: peripheral arterial tonometry
PI3K/Akt/mTOR: phosphatidylinositol 3-kinase/protein kinase B/mammalian target of rapamycin
PRISMA: the Preferred Reporting Items for Systematic Review and Meta-Analysis
PROSPERO: International Prospective Register of Systematic Reviews
PSV: peak systolic velocity
PWV: pulse wave velocity
RCTs: randomized controlled trials
RI: resistance index
RR: relative risk
SGLT-2: sodium-glucose cotransporter-2
SMD: standardized mean difference
SPIRIT: Standard Protocol Items: Recommendations for Interventional Trials
T2DM: type 2 diabetes mellitus
TCM: traditional Chinese medicine
TC: total cholesterol
TG: triglyceride
UKPDS: United Kingdom Prospective Diabetes Study
VIP: China Science and Technology Journal Database
WOS: Web of Science
WM: western medicine.
