# Supplementary material for: The efficacy and safety of Chinese herbal medicine as an add-on therapy for type 2 diabetes mellitus patients with carotid atherosclerosis: An updated meta-analysis of 27 randomized controlled trials
Source: Front Pharmacol. 2023 Mar 23;14:1091718. doi: 10.3389/fphar.2023.1091718 (PMC10076753; doi:10.3389/fphar.2023.1091718)
Supplement: Supplementary file 1 [file DataSheet1.docx]

**Contents Page**

[Supplementary Material S1. PRISMA 2020 checklist 1](#_Toc118636512)

[Supplementary Material S2. Database and Search Strategies 4](#_Toc118636513)

[Supplementary Material S3. Literature excluded after reading the full text and reasons 21](#_Toc118636514)

[Supplementary Material S4. Meta-regression of CIMT, TC, TG, LDL-C, HDL-C, FBG, 2hPG and HbA1c. 32](#_Toc118636515)

[Supplementary Material S5. Sensitivity analysis. 34](#_Toc118636516)

[Supplementary material S6. Subgroup analysis of Crouse score and HOMA-IR. 39](#_Toc118636517)

[Supplementary material S7. Publication Bias 42](#_Toc118636518)

[Supplementary Material S8. Assessment of evidence quality for each outcome 44](#_Toc118636519)

# Supplementary Material S1. PRISMA 2020 checklist

| **Section and Topic** | **Item #** | **Checklist item** | **Location where item is reported** |
| --- | --- | --- | --- |
| **TITLE** | | |  |
| Title | 1 | Identify the report as a systematic review. | P1 |
| **ABSTRACT** | | |  |
| Abstract | 2 | See the PRISMA 2020 for Abstracts checklist. | P2-3 |
| **INTRODUCTION** | | |  |
| Rationale | 3 | Describe the rationale for the review in the context of existing knowledge. | P4-5 |
| Objectives | 4 | Provide an explicit statement of the objective(s) or question(s) the review addresses. | P5 |
| **METHODS** | | |  |
| Eligibility criteria | 5 | Specify the inclusion and exclusion criteria for the review and how studies were grouped for the syntheses. | P6-7 |
| Information sources | 6 | Specify all databases, registers, websites, organisations, reference lists and other sources searched or consulted to identify studies. Specify the date when each source was last searched or consulted. | P5-6 |
| Search strategy | 7 | Present the full search strategies for all databases, registers and websites, including any filters and limits used. | Supplementary Material S2 |
| Selection process | 8 | Specify the methods used to decide whether a study met the inclusion criteria of the review, including how many reviewers screened each record and each report retrieved, whether they worked independently, and if applicable, details of automation tools used in the process. | P7-8 |
| Data collection process | 9 | Specify the methods used to collect data from reports, including how many reviewers collected data from each report, whether they worked independently, any processes for obtaining or confirming data from study investigators, and if applicable, details of automation tools used in the process. | P8 |
| Data items | 10a | List and define all outcomes for which data were sought. Specify whether all results that were compatible with each outcome domain in each study were sought (e.g. for all measures, time points, analyses), and if not, the methods used to decide which results to collect. | P6-7 |
|  | 10b | List and define all other variables for which data were sought (e.g. participant and intervention characteristics, funding sources). Describe any assumptions made about any missing or unclear information. | P8 |
| Study risk of bias assessment | 11 | Specify the methods used to assess risk of bias in the included studies, including details of the tool(s) used, how many reviewers assessed each study and whether they worked independently, and if applicable, details of automation tools used in the process. | P8 |
| Effect measures | 12 | Specify for each outcome the effect measure(s) (e.g. risk ratio, mean difference) used in the synthesis or presentation of results. | P8 |
| Synthesis methods | 13a | Describe the processes used to decide which studies were eligible for each synthesis (e.g. tabulating the study intervention characteristics and comparing against the planned groups for each synthesis (item #5)). | P8 |
|  | 13b | Describe any methods required to prepare the data for presentation or synthesis, such as handling of missing summary statistics, or data conversions. | P8 |
|  | 13c | Describe any methods used to tabulate or visually display results of individual studies and syntheses. | P8 |
|  | 13d | Describe any methods used to synthesize results and provide a rationale for the choice(s). If meta-analysis was performed, describe the model(s), method(s) to identify the presence and extent of statistical heterogeneity, and software package(s) used. | P8 |
|  | 13e | Describe any methods used to explore possible causes of heterogeneity among study results (e.g. subgroup analysis, meta-regression). | P8-9 |
|  | 13f | Describe any sensitivity analyses conducted to assess robustness of the synthesized results. | P8 |
| Reporting bias assessment | 14 | Describe any methods used to assess risk of bias due to missing results in a synthesis (arising from reporting biases). | P8 |
| Certainty assessment | 15 | Describe any methods used to assess certainty (or confidence) in the body of evidence for an outcome. | P8 |
| **RESULTS** | | |  |
| Study selection | 16a | Describe the results of the search and selection process, from the number of records identified in the search to the number of studies included in the review, ideally using a flow diagram. | P9, figure 1 |
|  | 16b | Cite studies that might appear to meet the inclusion criteria, but which were excluded, and explain why they were excluded. | Supplementary Material S3 |
| Study characteristics | 17 | Cite each included study and present its characteristics. | P9-10, table 1, table 2 |
| Risk of bias in studies | 18 | Present assessments of risk of bias for each included study. | P10, figure 2 |
| Results of individual studies | 19 | For all outcomes, present, for each study: (a) summary statistics for each group (where appropriate) and (b) an effect estimate and its precision (e.g. confidence/credible interval), ideally using structured tables or plots. | P11-17 |
| Results of syntheses | 20a | For each synthesis, briefly summarise the characteristics and risk of bias among contributing studies. | P11-17 |
|  | 20b | Present results of all statistical syntheses conducted. If meta-analysis was done, present for each the summary estimate and its precision (e.g. confidence/credible interval) and measures of statistical heterogeneity. If comparing groups, describe the direction of the effect. | P11-17 |
|  | 20c | Present results of all investigations of possible causes of heterogeneity among study results. | P11-17 |
|  | 20d | Present results of all sensitivity analyses conducted to assess the robustness of the synthesized results. | P11-17 |
| Reporting biases | 21 | Present assessments of risk of bias due to missing results (arising from reporting biases) for each synthesis assessed. | P17 |
| Certainty of evidence | 22 | Present assessments of certainty (or confidence) in the body of evidence for each outcome assessed. | P17, Supplementary material S8 |
| **DISCUSSION** | | |  |
| Discussion | 23a | Provide a general interpretation of the results in the context of other evidence. | P18-20 |
|  | 23b | Discuss any limitations of the evidence included in the review. | P22 |
|  | 23c | Discuss any limitations of the review processes used. | P22 |
|  | 23d | Discuss implications of the results for practice, policy, and future research. | P23 |
| **OTHER INFORMATION** | | |  |
| Registration and protocol | 24a | Provide registration information for the review, including register name and registration number, or state that the review was not registered. | P5,CRD42022346274 |
|  | 24b | Indicate where the review protocol can be accessed, or state that a protocol was not prepared. | - |
|  | 24c | Describe and explain any amendments to information provided at registration or in the protocol. | - |
| Support | 25 | Describe sources of financial or non-financial support for the review, and the role of the funders or sponsors in the review. | P30 |
| Competing interests | 26 | Declare any competing interests of review authors. | P30 |
| Availability of data, code and other materials | 27 | Report which of the following are publicly available and where they can be found: template data collection forms; data extracted from included studies; data used for all analyses; analytic code; any other materials used in the review. | P30 |

*From:* Page MJ, McKenzie JE, Bossuyt PM, Boutron I, Hoffmann TC, Mulrow CD, et al. The PRISMA 2020 statement: an updated guideline for reporting systematic reviews. BMJ 2021;372:n71. doi: 10.1136/bmj.n71 For more information, visit: http://www.prisma-statement.org/

# Supplementary Material S2. Database and Search Strategies

PubMed

The retrieval of the PubMed database was conducted on July 16, 2022, and 1 record was retrieved.

| Search | Query | Results | Time |
| --- | --- | --- | --- |
| #10 | Search: ((("Diabetes Mellitus, Type 2"[Mesh]) OR (((((((((((((((((((((((((((((((Diabetes Mellitus, Noninsulin-Dependent[Title/Abstract]) OR (Diabetes Mellitus, Ketosis-Resistant[Title/Abstract])) OR (Diabetes Mellitus, Ketosis Resistant[Title/Abstract])) OR (Ketosis-Resistant Diabetes Mellitus[Title/Abstract])) OR (Diabetes Mellitus, Non Insulin Dependent[Title/Abstract])) OR (Diabetes Mellitus, Non-Insulin-Dependent[Title/Abstract])) OR (Non-Insulin-Dependent Diabetes Mellitus[Title/Abstract])) OR (Diabetes Mellitus, Stable[Title/Abstract])) OR (Stable Diabetes Mellitus[Title/Abstract])) OR (Diabetes Mellitus, Type II[Title/Abstract])) OR (NIDDM[Title/Abstract])) OR (Diabetes Mellitus, Noninsulin Dependent[Title/Abstract])) OR (Diabetes Mellitus, Maturity-Onset[Title/Abstract])) OR (Diabetes Mellitus, Maturity Onset[Title/Abstract])) OR (Maturity-Onset Diabetes Mellitus[Title/Abstract])) OR (Maturity Onset Diabetes Mellitus[Title/Abstract])) OR (MODY[Title/Abstract])) OR (Diabetes Mellitus, Slow-Onset[Title/Abstract])) OR (Diabetes Mellitus, Slow Onset[Title/Abstract])) OR (Slow-Onset Diabetes Mellitus[Title/Abstract])) OR (Type 2 Diabetes Mellitus[Title/Abstract])) OR (Noninsulin-Dependent Diabetes Mellitus[Title/Abstract])) OR (Noninsulin Dependent Diabetes Mellitus[Title/Abstract])) OR (Maturity-Onset Diabetes[Title/Abstract])) OR (Diabetes, Maturity-Onset[Title/Abstract])) OR (Maturity Onset Diabetes[Title/Abstract])) OR (Type 2 Diabetes[Title/Abstract])) OR (Diabetes, Type 2[Title/Abstract])) OR (Diabetes Mellitus, Adult-Onset[Title/Abstract])) OR (Adult-Onset Diabetes Mellitus[Title/Abstract])) OR (Diabetes Mellitus, Adult Onset[Title/Abstract]))) AND (("Medicine, Chinese Traditional"[Mesh]) OR (((((((((((((((((((((((((((((((((((((((((Traditional Chinese Medicine[Title/Abstract]) OR (Chung I Hsueh[Title/Abstract])) OR (Hsueh, Chung I[Title/Abstract])) OR (Traditional Medicine, Chinese[Title/Abstract])) OR (Zhong Yi Xue[Title/Abstract])) OR (Chinese Traditional Medicine[Title/Abstract])) OR (Chinese Medicine, Traditional[Title/Abstract])) OR (Chinese Drugs, Plant[Title/Abstract])) OR (Chinese Herbal Drugs[Title/Abstract])) OR (Herbal Drugs, Chinese[Title/Abstract])) OR (Plant Extracts, Chinese[Title/Abstract])) OR (Chinese Plant Extracts[Title/Abstract])) OR (Extracts, Chinese Plant[Title/Abstract])) OR (Oriental Medicine, Traditional[Title/Abstract])) OR (Medicine, Traditional Oriental[Title/Abstract])) OR (Traditional Oriental Medicine[Title/Abstract])) OR (Traditional Oriental Medicines[Title/Abstract])) OR (Traditional Medicine, Oriental[Title/Abstract])) OR (Traditional East Asian Medicine[Title/Abstract])) OR (Medicine, Traditional, East Asia[Title/Abstract])) OR (Traditional Medicine, East Asia[Title/Abstract])) OR (Traditional Far Eastern Medicine[Title/Abstract])) OR (East Asian Traditional Medicine[Title/Abstract])) OR (Oriental Traditional Medicine[Title/Abstract])) OR (Medicine, Oriental Traditional[Title/Abstract])) OR (East Asian Medicine[Title/Abstract])) OR (East Asian Medicines[Title/Abstract])) OR (Medicine, East Asian[Title/Abstract])) OR (Oriental Medicine[Title/Abstract])) OR (Medicine, Far East[Title/Abstract])) OR (East Medicine, Far[Title/Abstract])) OR (East Medicines, Far[Title/Abstract])) OR (Far East Medicine[Title/Abstract])) OR (Far East Medicines[Title/Abstract])) OR (Medicines, Far East[Title/Abstract])) OR (Medicine, East Asia[Title/Abstract])) OR (Asia Medicines, East[Title/Abstract])) OR (East Asia Medicine[Title/Abstract])) OR (East Asia Medicines[Title/Abstract])) OR (Medicines, East Asia[Title/Abstract])) OR (Medicine, Oriental[Title/Abstract])))) AND ((("Carotid Artery Diseases"[Mesh]) OR "Carotid Intima-Media Thickness"[Mesh]) OR ((((((((((((((((((((((((((((((((((Artery Disease, Carotid[Title/Abstract]) OR (Artery Diseases, Carotid[Title/Abstract])) OR (Carotid Artery Disease[Title/Abstract])) OR (Carotid Artery Disorders[Title/Abstract])) OR (Artery Disorder, Carotid[Title/Abstract])) OR (Artery Disorders, Carotid[Title/Abstract])) OR (Carotid Artery Disorder[Title/Abstract])) OR (Disorders, Carotid Artery[Title/Abstract])) OR (Arterial Diseases, Carotid[Title/Abstract])) OR (Arterial Disease, Carotid[Title/Abstract])) OR (Carotid Arterial Disease[Title/Abstract])) OR (Carotid Arterial Diseases[Title/Abstract])) OR (Carotid Atherosclerosis[Title/Abstract])) OR (Carotid Atheroscleroses[Title/Abstract])) OR (Carotid Atherosclerotic Disease[Title/Abstract])) OR (Atherosclerotic Disease, Carotid[Title/Abstract])) OR (Atherosclerotic Diseases, Carotid[Title/Abstract])) OR (Carotid Atherosclerotic Diseases[Title/Abstract])) OR (Internal Carotid Artery Diseases[Title/Abstract])) OR (Arterial Diseases, Internal Carotid[Title/Abstract])) OR (Internal Carotid Artery Disease[Title/Abstract])) OR (Arterial Diseases, Common Carotid[Title/Abstract])) OR (Common Carotid Artery Diseases[Title/Abstract])) OR (Common Carotid Artery Disease[Title/Abstract])) OR (External Carotid Artery Diseases[Title/Abstract])) OR (Arterial Diseases, External Carotid[Title/Abstract])) OR (Carotid Intima Media Thickness[Title/Abstract])) OR (Intima-Media Thickness, Carotid[Title/Abstract])) OR (Carotid Thickening[Title/Abstract])) OR (Carotid Atherosclerotic Plaque[Title/Abstract])) OR (Carotid Plaque[Title/Abstract])) OR (Carotid Artery Plaque[Title/Abstract])) OR (Carotid Arterial Plaque[Title/Abstract])) OR (Carotid Stenosis[Title/Abstract]))) Sort by: Most Recent | 1 | 11:56:23 |
| #9 | Search: (("Carotid Artery Diseases"[Mesh]) OR "Carotid Intima-Media Thickness"[Mesh]) OR ((((((((((((((((((((((((((((((((((Artery Disease, Carotid[Title/Abstract]) OR (Artery Diseases, Carotid[Title/Abstract])) OR (Carotid Artery Disease[Title/Abstract])) OR (Carotid Artery Disorders[Title/Abstract])) OR (Artery Disorder, Carotid[Title/Abstract])) OR (Artery Disorders, Carotid[Title/Abstract])) OR (Carotid Artery Disorder[Title/Abstract])) OR (Disorders, Carotid Artery[Title/Abstract])) OR (Arterial Diseases, Carotid[Title/Abstract])) OR (Arterial Disease, Carotid[Title/Abstract])) OR (Carotid Arterial Disease[Title/Abstract])) OR (Carotid Arterial Diseases[Title/Abstract])) OR (Carotid Atherosclerosis[Title/Abstract])) OR (Carotid Atheroscleroses[Title/Abstract])) OR (Carotid Atherosclerotic Disease[Title/Abstract])) OR (Atherosclerotic Disease, Carotid[Title/Abstract])) OR (Atherosclerotic Diseases, Carotid[Title/Abstract])) OR (Carotid Atherosclerotic Diseases[Title/Abstract])) OR (Internal Carotid Artery Diseases[Title/Abstract])) OR (Arterial Diseases, Internal Carotid[Title/Abstract])) OR (Internal Carotid Artery Disease[Title/Abstract])) OR (Arterial Diseases, Common Carotid[Title/Abstract])) OR (Common Carotid Artery Diseases[Title/Abstract])) OR (Common Carotid Artery Disease[Title/Abstract])) OR (External Carotid Artery Diseases[Title/Abstract])) OR (Arterial Diseases, External Carotid[Title/Abstract])) OR (Carotid Intima Media Thickness[Title/Abstract])) OR (Intima-Media Thickness, Carotid[Title/Abstract])) OR (Carotid Thickening[Title/Abstract])) OR (Carotid Atherosclerotic Plaque[Title/Abstract])) OR (Carotid Plaque[Title/Abstract])) OR (Carotid Artery Plaque[Title/Abstract])) OR (Carotid Arterial Plaque[Title/Abstract])) OR (Carotid Stenosis[Title/Abstract])) Sort by: Most Recent | 81,989 | 11:31:25 |
| #8 | Search: (((((((((((((((((((((((((((((((((Artery Disease, Carotid[Title/Abstract]) OR (Artery Diseases, Carotid[Title/Abstract])) OR (Carotid Artery Disease[Title/Abstract])) OR (Carotid Artery Disorders[Title/Abstract])) OR (Artery Disorder, Carotid[Title/Abstract])) OR (Artery Disorders, Carotid[Title/Abstract])) OR (Carotid Artery Disorder[Title/Abstract])) OR (Disorders, Carotid Artery[Title/Abstract])) OR (Arterial Diseases, Carotid[Title/Abstract])) OR (Arterial Disease, Carotid[Title/Abstract])) OR (Carotid Arterial Disease[Title/Abstract])) OR (Carotid Arterial Diseases[Title/Abstract])) OR (Carotid Atherosclerosis[Title/Abstract])) OR (Carotid Atheroscleroses[Title/Abstract])) OR (Carotid Atherosclerotic Disease[Title/Abstract])) OR (Atherosclerotic Disease, Carotid[Title/Abstract])) OR (Atherosclerotic Diseases, Carotid[Title/Abstract])) OR (Carotid Atherosclerotic Diseases[Title/Abstract])) OR (Internal Carotid Artery Diseases[Title/Abstract])) OR (Arterial Diseases, Internal Carotid[Title/Abstract])) OR (Internal Carotid Artery Disease[Title/Abstract])) OR (Arterial Diseases, Common Carotid[Title/Abstract])) OR (Common Carotid Artery Diseases[Title/Abstract])) OR (Common Carotid Artery Disease[Title/Abstract])) OR (External Carotid Artery Diseases[Title/Abstract])) OR (Arterial Diseases, External Carotid[Title/Abstract])) OR (Carotid Intima Media Thickness[Title/Abstract])) OR (Intima-Media Thickness, Carotid[Title/Abstract])) OR (Carotid Thickening[Title/Abstract])) OR (Carotid Atherosclerotic Plaque[Title/Abstract])) OR (Carotid Plaque[Title/Abstract])) OR (Carotid Artery Plaque[Title/Abstract])) OR (Carotid Arterial Plaque[Title/Abstract])) OR (Carotid Stenosis[Title/Abstract]) Sort by: Most Recent | 56,886 | 11:28:10 |
| #7 | Search: ("Carotid Artery Diseases"[Mesh]) OR "Carotid Intima-Media Thickness"[Mesh] Sort by: Most Recent | 55,597 | 11:20:13 |
| #6 | Search: ("Medicine, Chinese Traditional"[Mesh]) OR (((((((((((((((((((((((((((((((((((((((((Traditional Chinese Medicine[Title/Abstract]) OR (Chung I Hsueh[Title/Abstract])) OR (Hsueh, Chung I[Title/Abstract])) OR (Traditional Medicine, Chinese[Title/Abstract])) OR (Zhong Yi Xue[Title/Abstract])) OR (Chinese Traditional Medicine[Title/Abstract])) OR (Chinese Medicine, Traditional[Title/Abstract])) OR (Chinese Drugs, Plant[Title/Abstract])) OR (Chinese Herbal Drugs[Title/Abstract])) OR (Herbal Drugs, Chinese[Title/Abstract])) OR (Plant Extracts, Chinese[Title/Abstract])) OR (Chinese Plant Extracts[Title/Abstract])) OR (Extracts, Chinese Plant[Title/Abstract])) OR (Oriental Medicine, Traditional[Title/Abstract])) OR (Medicine, Traditional Oriental[Title/Abstract])) OR (Traditional Oriental Medicine[Title/Abstract])) OR (Traditional Oriental Medicines[Title/Abstract])) OR (Traditional Medicine, Oriental[Title/Abstract])) OR (Traditional East Asian Medicine[Title/Abstract])) OR (Medicine, Traditional, East Asia[Title/Abstract])) OR (Traditional Medicine, East Asia[Title/Abstract])) OR (Traditional Far Eastern Medicine[Title/Abstract])) OR (East Asian Traditional Medicine[Title/Abstract])) OR (Oriental Traditional Medicine[Title/Abstract])) OR (Medicine, Oriental Traditional[Title/Abstract])) OR (East Asian Medicine[Title/Abstract])) OR (East Asian Medicines[Title/Abstract])) OR (Medicine, East Asian[Title/Abstract])) OR (Oriental Medicine[Title/Abstract])) OR (Medicine, Far East[Title/Abstract])) OR (East Medicine, Far[Title/Abstract])) OR (East Medicines, Far[Title/Abstract])) OR (Far East Medicine[Title/Abstract])) OR (Far East Medicines[Title/Abstract])) OR (Medicines, Far East[Title/Abstract])) OR (Medicine, East Asia[Title/Abstract])) OR (Asia Medicines, East[Title/Abstract])) OR (East Asia Medicine[Title/Abstract])) OR (East Asia Medicines[Title/Abstract])) OR (Medicines, East Asia[Title/Abstract])) OR (Medicine, Oriental[Title/Abstract])) Sort by: Most Recent | 60,668 | 11:13:59 |
| #5 | Search: ((((((((((((((((((((((((((((((((((((((((Traditional Chinese Medicine[Title/Abstract]) OR (Chung I Hsueh[Title/Abstract])) OR (Hsueh, Chung I[Title/Abstract])) OR (Traditional Medicine, Chinese[Title/Abstract])) OR (Zhong Yi Xue[Title/Abstract])) OR (Chinese Traditional Medicine[Title/Abstract])) OR (Chinese Medicine, Traditional[Title/Abstract])) OR (Chinese Drugs, Plant[Title/Abstract])) OR (Chinese Herbal Drugs[Title/Abstract])) OR (Herbal Drugs, Chinese[Title/Abstract])) OR (Plant Extracts, Chinese[Title/Abstract])) OR (Chinese Plant Extracts[Title/Abstract])) OR (Extracts, Chinese Plant[Title/Abstract])) OR (Oriental Medicine, Traditional[Title/Abstract])) OR (Medicine, Traditional Oriental[Title/Abstract])) OR (Traditional Oriental Medicine[Title/Abstract])) OR (Traditional Oriental Medicines[Title/Abstract])) OR (Traditional Medicine, Oriental[Title/Abstract])) OR (Traditional East Asian Medicine[Title/Abstract])) OR (Medicine, Traditional, East Asia[Title/Abstract])) OR (Traditional Medicine, East Asia[Title/Abstract])) OR (Traditional Far Eastern Medicine[Title/Abstract])) OR (East Asian Traditional Medicine[Title/Abstract])) OR (Oriental Traditional Medicine[Title/Abstract])) OR (Medicine, Oriental Traditional[Title/Abstract])) OR (East Asian Medicine[Title/Abstract])) OR (East Asian Medicines[Title/Abstract])) OR (Medicine, East Asian[Title/Abstract])) OR (Oriental Medicine[Title/Abstract])) OR (Medicine, Far East[Title/Abstract])) OR (East Medicine, Far[Title/Abstract])) OR (East Medicines, Far[Title/Abstract])) OR (Far East Medicine[Title/Abstract])) OR (Far East Medicines[Title/Abstract])) OR (Medicines, Far East[Title/Abstract])) OR (Medicine, East Asia[Title/Abstract])) OR (Asia Medicines, East[Title/Abstract])) OR (East Asia Medicine[Title/Abstract])) OR (East Asia Medicines[Title/Abstract])) OR (Medicines, East Asia[Title/Abstract])) OR (Medicine, Oriental[Title/Abstract]) Sort by: Most Recent | 47,819 | 11:11:15 |
| #4 | Search: "Medicine, Chinese Traditional"[Mesh] Sort by: Most Recent | 22,526 | 10:51:33 |
| #3 | Search: ("Diabetes Mellitus, Type 2"[Mesh]) OR (((((((((((((((((((((((((((((((Diabetes Mellitus, Noninsulin-Dependent[Title/Abstract]) OR (Diabetes Mellitus, Ketosis-Resistant[Title/Abstract])) OR (Diabetes Mellitus, Ketosis Resistant[Title/Abstract])) OR (Ketosis-Resistant Diabetes Mellitus[Title/Abstract])) OR (Diabetes Mellitus, Non Insulin Dependent[Title/Abstract])) OR (Diabetes Mellitus, Non-Insulin-Dependent[Title/Abstract])) OR (Non-Insulin-Dependent Diabetes Mellitus[Title/Abstract])) OR (Diabetes Mellitus, Stable[Title/Abstract])) OR (Stable Diabetes Mellitus[Title/Abstract])) OR (Diabetes Mellitus, Type II[Title/Abstract])) OR (NIDDM[Title/Abstract])) OR (Diabetes Mellitus, Noninsulin Dependent[Title/Abstract])) OR (Diabetes Mellitus, Maturity-Onset[Title/Abstract])) OR (Diabetes Mellitus, Maturity Onset[Title/Abstract])) OR (Maturity-Onset Diabetes Mellitus[Title/Abstract])) OR (Maturity Onset Diabetes Mellitus[Title/Abstract])) OR (MODY[Title/Abstract])) OR (Diabetes Mellitus, Slow-Onset[Title/Abstract])) OR (Diabetes Mellitus, Slow Onset[Title/Abstract])) OR (Slow-Onset Diabetes Mellitus[Title/Abstract])) OR (Type 2 Diabetes Mellitus[Title/Abstract])) OR (Noninsulin-Dependent Diabetes Mellitus[Title/Abstract])) OR (Noninsulin Dependent Diabetes Mellitus[Title/Abstract])) OR (Maturity-Onset Diabetes[Title/Abstract])) OR (Diabetes, Maturity-Onset[Title/Abstract])) OR (Maturity Onset Diabetes[Title/Abstract])) OR (Type 2 Diabetes[Title/Abstract])) OR (Diabetes, Type 2[Title/Abstract])) OR (Diabetes Mellitus, Adult-Onset[Title/Abstract])) OR (Adult-Onset Diabetes Mellitus[Title/Abstract])) OR (Diabetes Mellitus, Adult Onset[Title/Abstract])) Sort by: Most Recent | 216,947 | 10:48:10 |
| #2 | Search: ((((((((((((((((((((((((((((((Diabetes Mellitus, Noninsulin-Dependent[Title/Abstract]) OR (Diabetes Mellitus, Ketosis-Resistant[Title/Abstract])) OR (Diabetes Mellitus, Ketosis Resistant[Title/Abstract])) OR (Ketosis-Resistant Diabetes Mellitus[Title/Abstract])) OR (Diabetes Mellitus, Non Insulin Dependent[Title/Abstract])) OR (Diabetes Mellitus, Non-Insulin-Dependent[Title/Abstract])) OR (Non-Insulin-Dependent Diabetes Mellitus[Title/Abstract])) OR (Diabetes Mellitus, Stable[Title/Abstract])) OR (Stable Diabetes Mellitus[Title/Abstract])) OR (Diabetes Mellitus, Type II[Title/Abstract])) OR (NIDDM[Title/Abstract])) OR (Diabetes Mellitus, Noninsulin Dependent[Title/Abstract])) OR (Diabetes Mellitus, Maturity-Onset[Title/Abstract])) OR (Diabetes Mellitus, Maturity Onset[Title/Abstract])) OR (Maturity-Onset Diabetes Mellitus[Title/Abstract])) OR (Maturity Onset Diabetes Mellitus[Title/Abstract])) OR (MODY[Title/Abstract])) OR (Diabetes Mellitus, Slow-Onset[Title/Abstract])) OR (Diabetes Mellitus, Slow Onset[Title/Abstract])) OR (Slow-Onset Diabetes Mellitus[Title/Abstract])) OR (Type 2 Diabetes Mellitus[Title/Abstract])) OR (Noninsulin-Dependent Diabetes Mellitus[Title/Abstract])) OR (Noninsulin Dependent Diabetes Mellitus[Title/Abstract])) OR (Maturity-Onset Diabetes[Title/Abstract])) OR (Diabetes, Maturity-Onset[Title/Abstract])) OR (Maturity Onset Diabetes[Title/Abstract])) OR (Type 2 Diabetes[Title/Abstract])) OR (Diabetes, Type 2[Title/Abstract])) OR (Diabetes Mellitus, Adult-Onset[Title/Abstract])) OR (Adult-Onset Diabetes Mellitus[Title/Abstract])) OR (Diabetes Mellitus, Adult Onset[Title/Abstract]) Sort by: Most Recent | 166,638 | 10:42:50 |
| #1 | Search: "Diabetes Mellitus, Type 2"[Mesh] Sort by: Most Recent | 159,313 | 10:33:50 |

Embase

The retrieval of the Embase database was conducted on July 16, 2022, and a total of 8 records were retrieved.

| History |  | Results |
| --- | --- | --- |
| #11 | #3 AND #6 AND #10 | 8 |
| #10 | #7 OR #8 OR #9 | 36,279 |
| #9 | 'carotid artery diseases':ab,ti OR 'artery disease, carotid':ab,ti OR 'artery diseases, carotid':ab,ti OR 'carotid artery disease':ab,ti OR 'carotid artery disorders':ab,ti OR 'artery disorder, carotid':ab,ti OR 'artery disorders, carotid':ab,ti OR 'carotid artery disorder':ab,ti OR 'disorders, carotid artery':ab,ti OR 'arterial diseases, carotid':ab,ti OR 'arterial disease, carotid':ab,ti OR 'carotid arterial disease':ab,ti OR 'carotid arterial diseases':ab,ti OR 'carotid atheroscleroses':ab,ti OR 'carotid atherosclerotic disease':ab,ti OR 'atherosclerotic disease, carotid':ab,ti OR 'atherosclerotic diseases, carotid':ab,ti OR 'carotid atherosclerotic diseases':ab,ti OR 'internal carotid artery diseases':ab,ti OR 'arterial diseases, internal carotid':ab,ti OR 'internal carotid artery disease':ab,ti OR 'arterial diseases, common carotid':ab,ti OR 'common carotid artery diseases':ab,ti OR 'common carotid artery disease':ab,ti OR 'external carotid artery diseases':ab,ti OR 'arterial diseases, external carotid':ab,ti OR 'carotid intima media thickness':ab,ti OR 'intima-media thickness, carotid':ab,ti OR 'carotid thickening':ab,ti OR 'carotid atherosclerotic plaque':ab,ti OR 'carotid plaque':ab,ti OR 'carotid artery plaque':ab,ti OR 'carotid arterial plaque':ab,ti OR 'carotid stenosis':ab,ti | 30,116 |
| #8 | 'carotid intima-media thickness'/exp | 2,141 |
| #7 | 'carotid atherosclerosis'/exp | 10,503 |
| #6 | #4 OR #5 | 79,133 |
| #5 | 'medicine, chinese traditional':ab,ti OR 'traditional chinese medicine':ab,ti OR 'chung i hsueh':ab,ti OR 'hsueh, chung i':ab,ti OR 'traditional medicine, chinese':ab,ti OR 'zhong yi xue':ab,ti OR 'chinese traditional medicine':ab,ti OR 'chinese medicine, traditional':ab,ti OR 'chinese drugs, plant':ab,ti OR 'chinese herbal drugs':ab,ti OR 'herbal drugs, chinese':ab,ti OR 'plant extracts, chinese':ab,ti OR 'chinese plant extracts':ab,ti OR 'extracts, chinese plant':ab,ti OR 'oriental medicine, traditional':ab,ti OR 'medicine, traditional oriental':ab,ti OR 'traditional oriental medicine':ab,ti OR 'traditional oriental medicines':ab,ti OR 'traditional medicine, oriental':ab,ti OR 'traditional east asian medicine':ab,ti OR 'medicine, traditional, east asia':ab,ti OR 'traditional medicine, east asia':ab,ti OR 'traditional far eastern medicine':ab,ti OR 'east asian traditional medicine':ab,ti OR 'oriental traditional medicine':ab,ti OR 'medicine, oriental traditional':ab,ti OR 'east asian medicine':ab,ti OR 'east asian medicines':ab,ti OR 'medicine, east asian':ab,ti OR 'oriental medicine':ab,ti OR 'medicine, far east':ab,ti OR 'east medicine, far':ab,ti OR 'east medicines, far':ab,ti OR 'far east medicine':ab,ti OR 'far east medicines':ab,ti OR 'medicines, far east':ab,ti OR 'medicine, east asia':ab,ti OR 'asia medicines, east':ab,ti OR 'east asia medicine':ab,ti OR 'east asia medicines':ab,ti OR 'medicines, east asia':ab,ti OR 'medicine, oriental':ab,ti | 37,435 |
| #4 | 'chinese medicine'/exp | 64,311 |
| #3 | #1 OR #2 | 350,623 |
| #2 | 'diabetes mellitus, type 2':ab,ti OR 'diabetes mellitus, noninsulin-dependent':ab,ti OR 'diabetes mellitus, ketosis-resistant':ab,ti OR 'diabetes mellitus, ketosis resistant':ab,ti OR 'ketosis-resistant diabetes mellitus':ab,ti OR 'diabetes mellitus, non insulin dependent':ab,ti OR 'diabetes mellitus, non-insulin-dependent':ab,ti OR 'non-insulin-dependent diabetes mellitus':ab,ti OR 'diabetes mellitus, stable':ab,ti OR 'stable diabetes mellitus':ab,ti OR 'diabetes mellitus, type ii':ab,ti OR niddm:ab,ti OR 'diabetes mellitus, noninsulin dependent':ab,ti OR 'diabetes mellitus, maturity-onset':ab,ti OR 'diabetes mellitus, maturity onset':ab,ti OR 'maturity-onset diabetes mellitus':ab,ti OR 'maturity onset diabetes mellitus':ab,ti OR mody:ab,ti OR 'diabetes mellitus, slow-onset':ab,ti OR 'diabetes mellitus, slow onset':ab,ti OR 'slow-onset diabetes mellitus':ab,ti OR 'type 2 diabetes mellitus':ab,ti OR 'noninsulin-dependent diabetes mellitus':ab,ti OR 'noninsulin dependent diabetes mellitus':ab,ti OR 'maturity-onset diabetes':ab,ti OR 'diabetes, maturity-onset':ab,ti OR 'maturity onset diabetes':ab,ti OR 'type 2 diabetes':ab,ti OR 'diabetes, type 2':ab,ti OR 'diabetes mellitus, adult-onset':ab,ti OR 'adult-onset diabetes mellitus':ab,ti OR 'diabetes mellitus, adult onset':ab,ti | 247,686 |
| #1 | 'non insulin dependent diabetes mellitus'/exp | 306,398 |


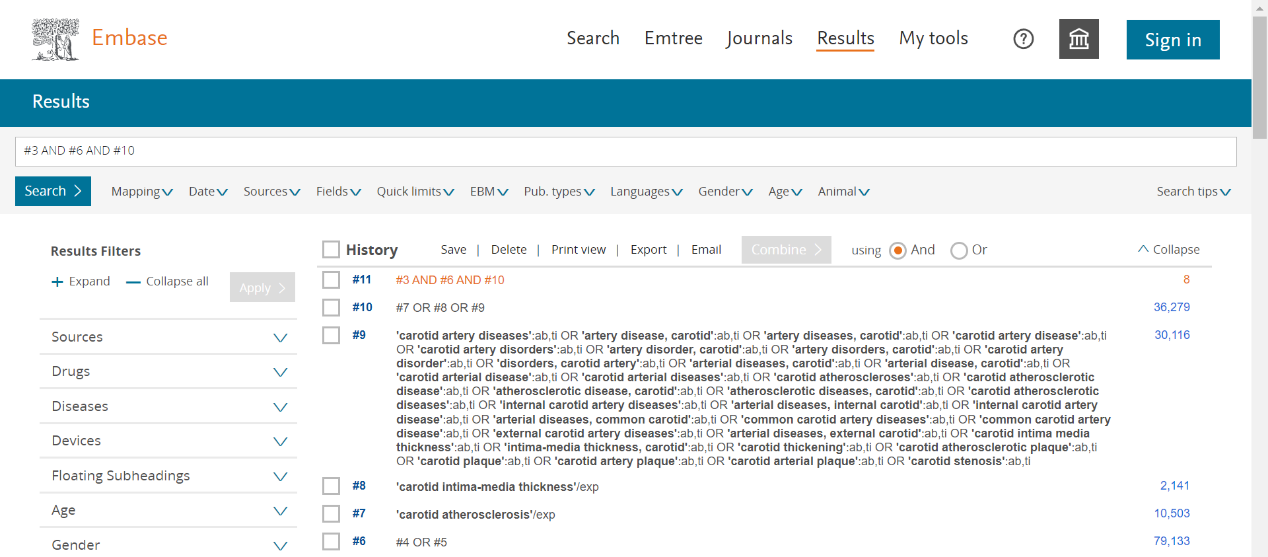

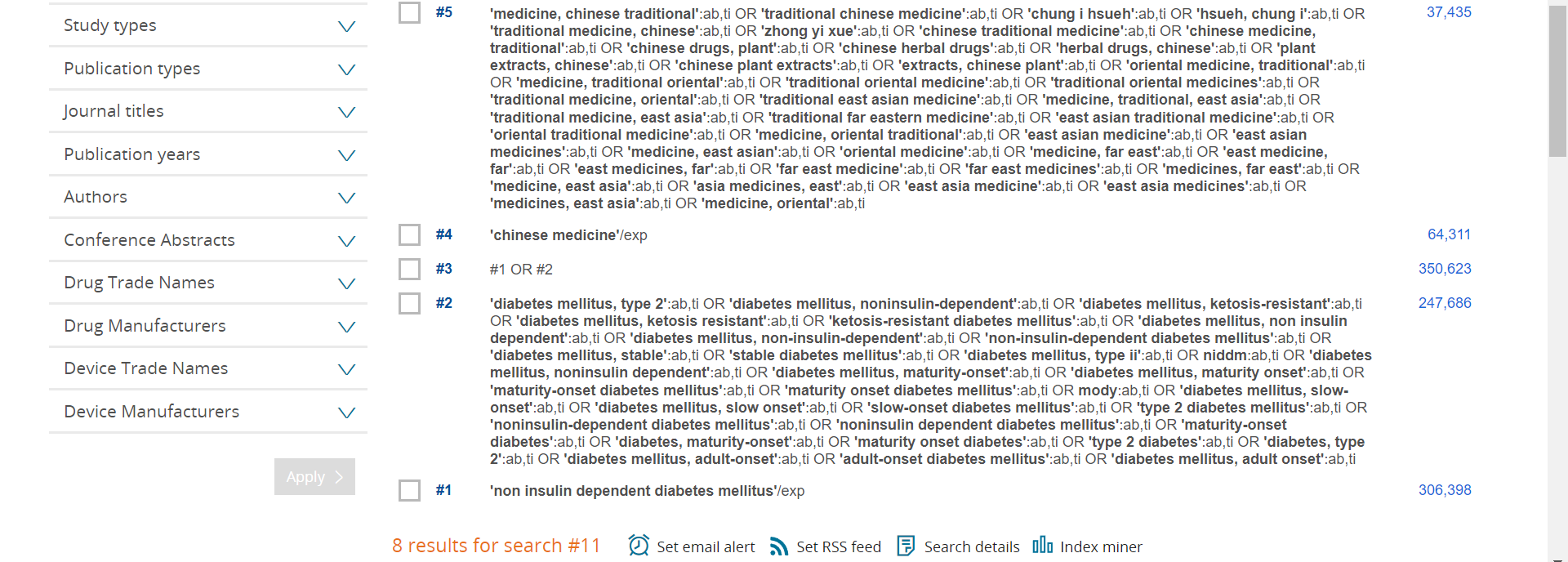


Cochrane

The retrieval of the Cochrane Library was conducted on July 16, 2022, and a total of 10 records were retrieved.


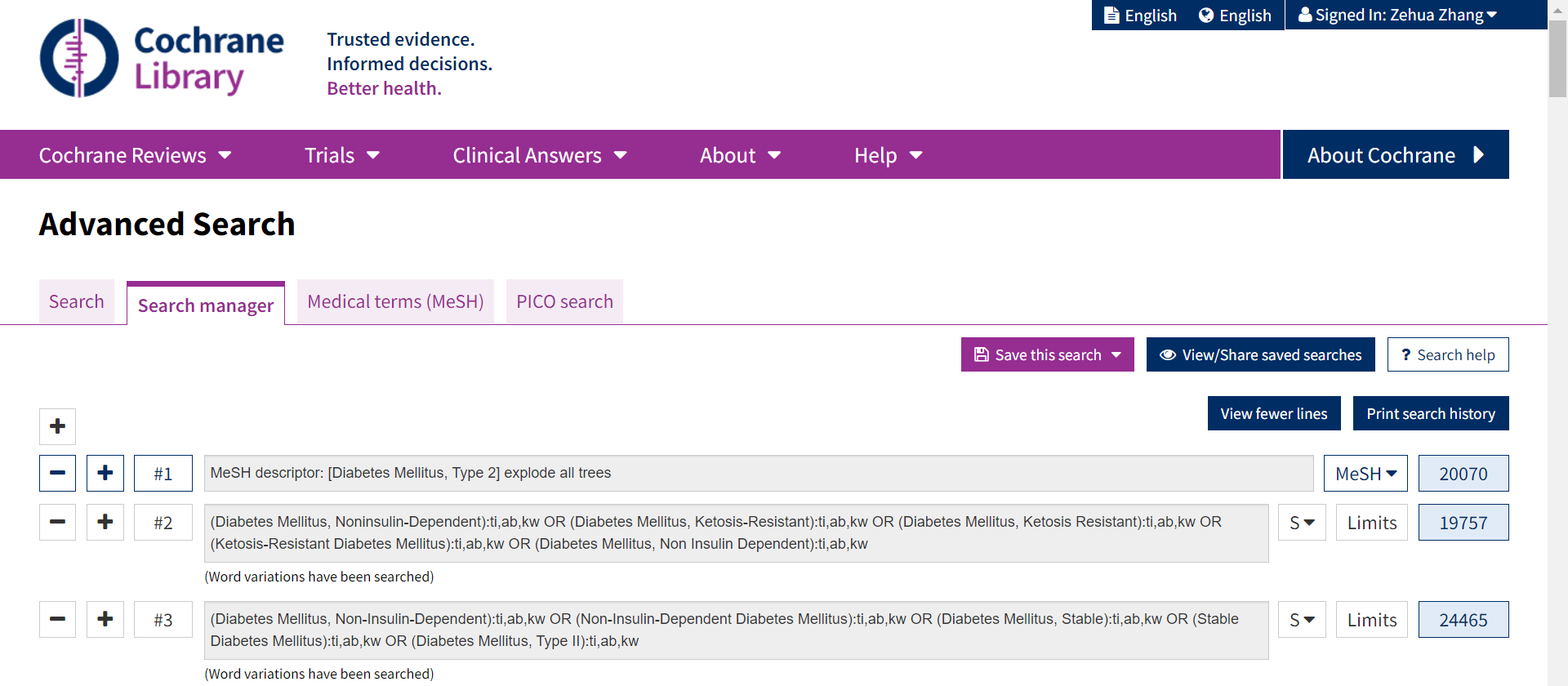


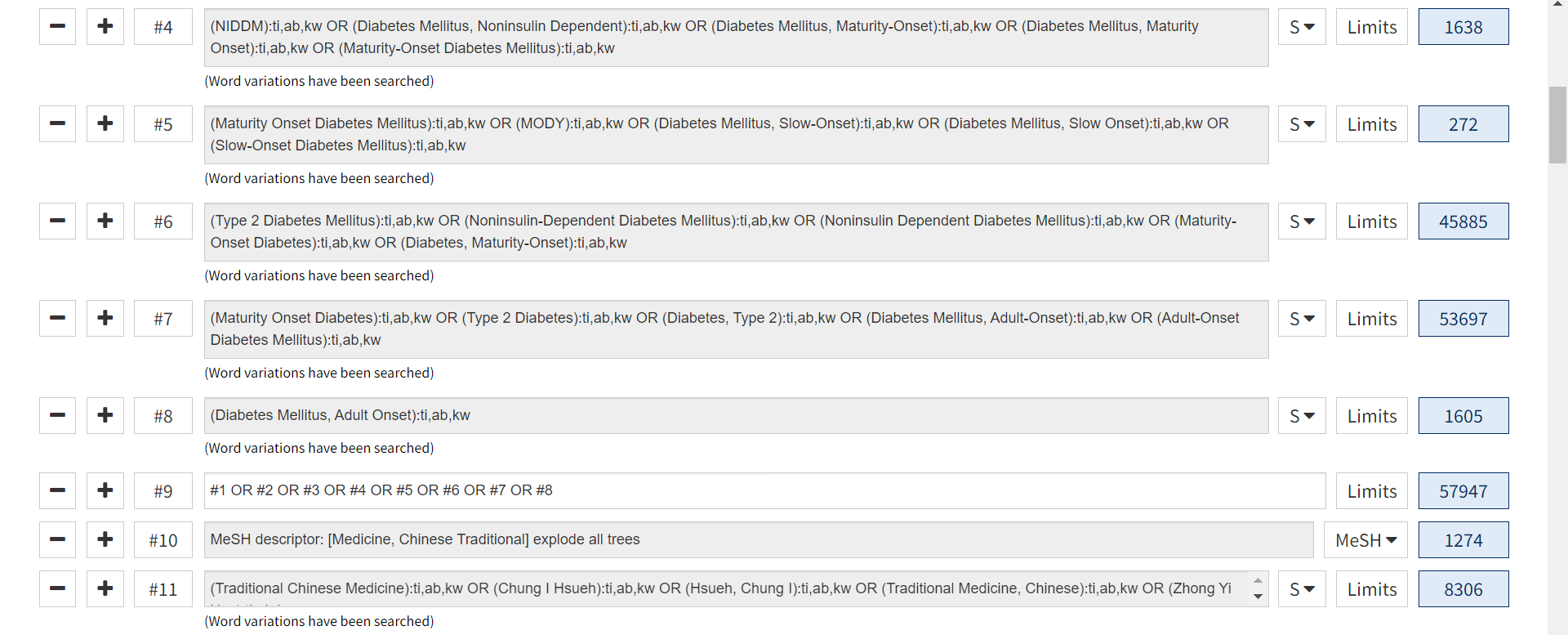


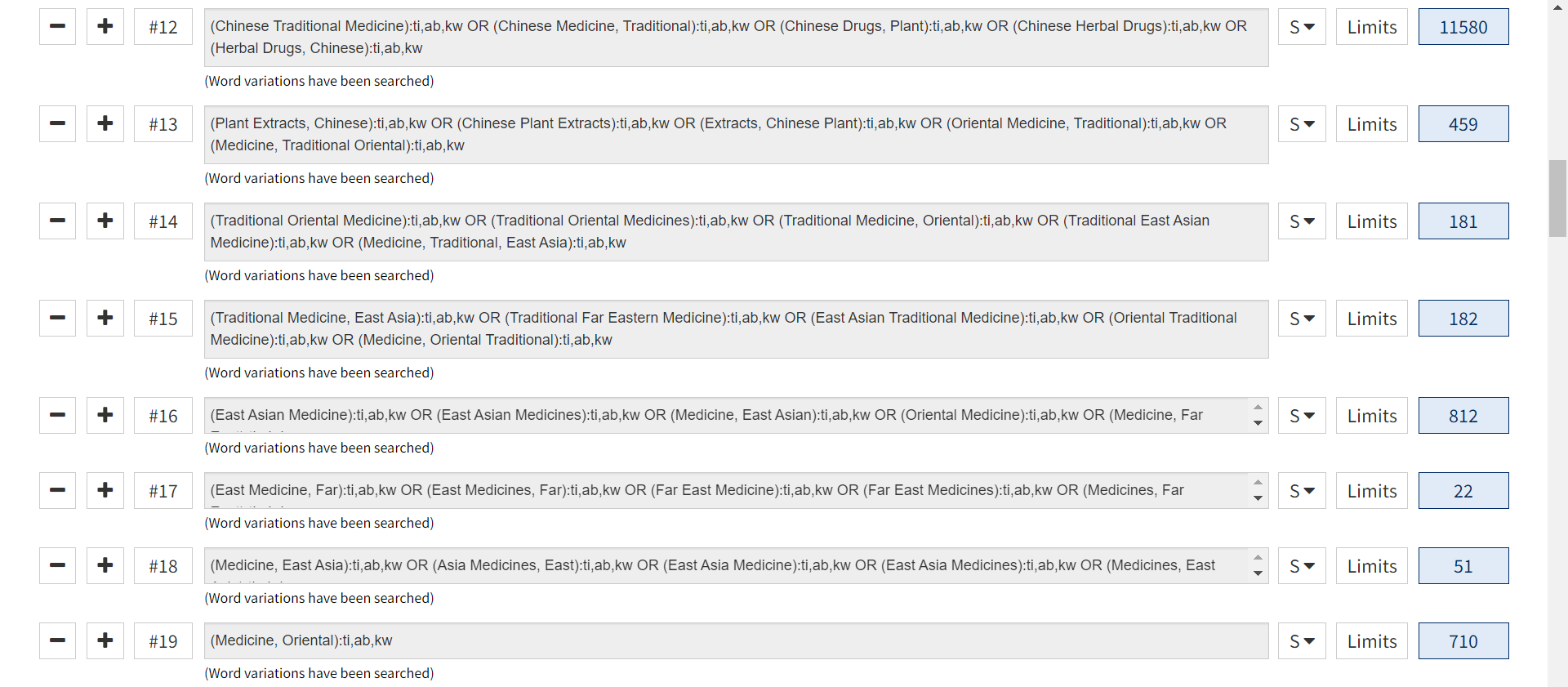


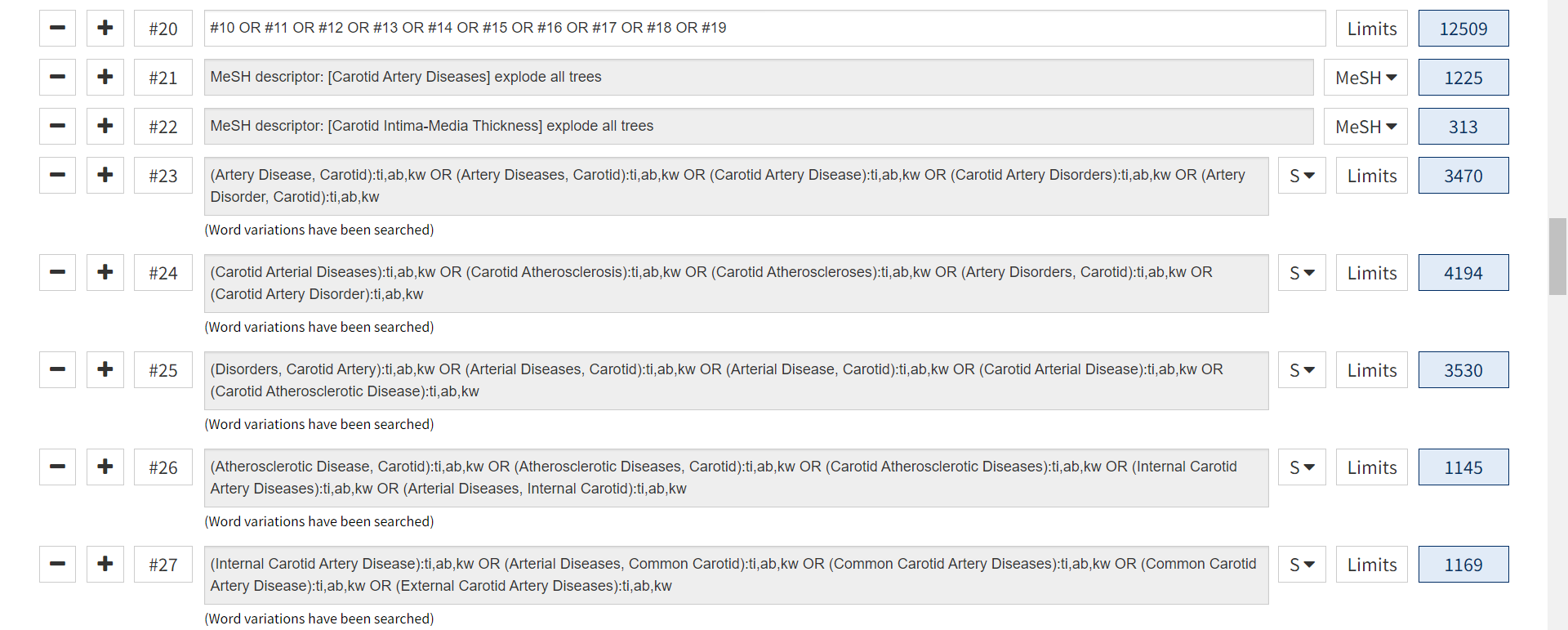


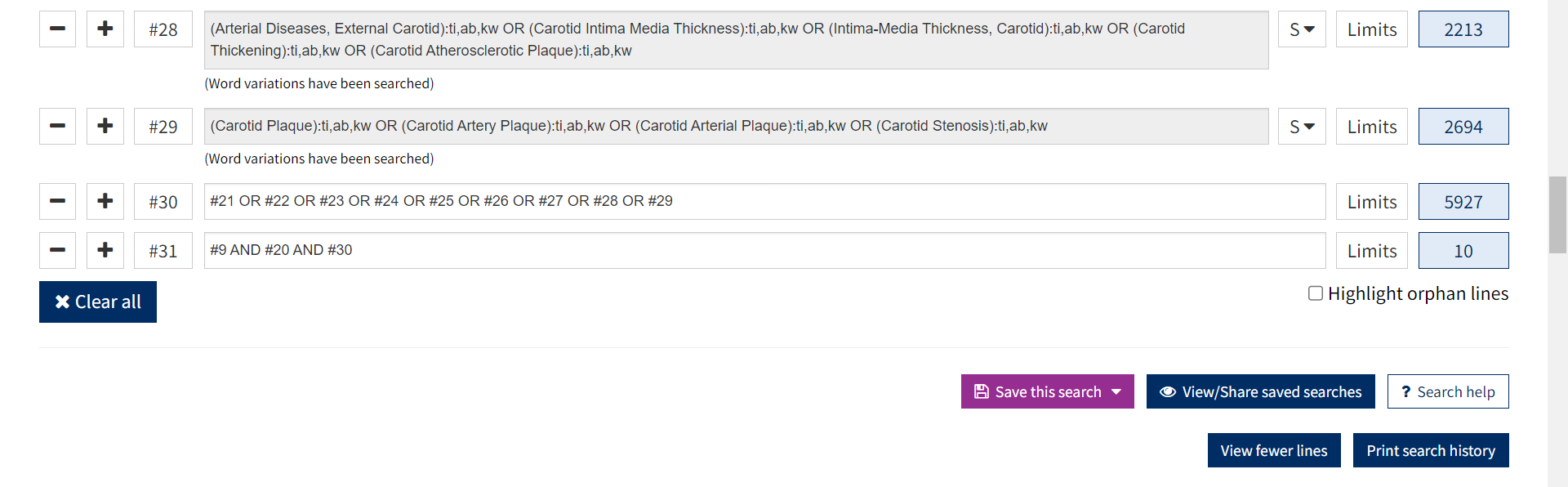


WOS

The retrieval of the WOS database was conducted on July 16, 2022, and 1 record was retrieved.

| History |  | Results |
| --- | --- | --- |
| #4 | #3 AND #2 AND #1 | 1 |
| #3 | TS=(Carotid Artery Diseases OR Carotid Intima-Media Thickness OR Artery Disease, Carotid OR Artery Diseases, Carotid OR Carotid Artery Disease OR Carotid Artery Disorders OR Artery Disorder, Carotid OR Artery Disorders, Carotid OR Carotid Artery Disorder OR Disorders, Carotid Artery OR Arterial Diseases, Carotid OR Arterial Disease, Carotid OR Carotid Arterial Disease OR Carotid Arterial Diseases OR Carotid Atherosclerosis OR Carotid Atheroscleroses OR Carotid Atherosclerotic Disease OR Atherosclerotic Disease, Carotid OR Atherosclerotic Diseases, Carotid OR Carotid Atherosclerotic Diseases OR Internal Carotid Artery Diseases OR Arterial Diseases, Internal Carotid OR Internal Carotid Artery Disease OR Arterial Diseases, Common Carotid OR Common Carotid Artery Diseases OR Common Carotid Artery Disease OR External Carotid Artery Diseases OR Arterial Diseases, External Carotid OR Carotid Intima Media Thickness OR Intima-Media Thickness, Carotid OR Carotid Thickening OR Carotid Atherosclerotic Plaque OR Carotid Plaque OR Carotid Artery Plaque OR Carotid Arterial Plaque OR Carotid Stenosis) | 34,124 |
| #2 | TS=(Medicine, Chinese Traditional OR Traditional Chinese Medicine OR Chung I Hsueh OR Hsueh, Chung I OR Traditional Medicine, Chinese OR Zhong Yi Xue OR Chinese Traditional Medicine OR Chinese Medicine, Traditional OR Chinese Drugs, Plant OR Chinese Herbal Drugs OR Herbal Drugs, Chinese OR Plant Extracts, Chinese OR Chinese Plant Extracts OR Extracts, Chinese Plant OR Oriental Medicine, Traditional OR Medicine, Traditional Oriental OR Traditional Oriental Medicine OR Traditional Oriental Medicines OR Traditional Medicine, Oriental OR Traditional East Asian Medicine OR Medicine, Traditional, East Asia OR Traditional Medicine, East Asia OR Traditional Far Eastern Medicine OR East Asian Traditional Medicine OR Oriental Traditional Medicine OR Medicine, Oriental Traditional OR East Asian Medicine OR East Asian Medicines OR Medicine, East Asian OR Oriental Medicine OR Medicine, Far East OR East Medicine, Far OR East Medicines, Far OR Far East Medicine OR Far East Medicines OR Medicines, Far East OR Medicine, East Asia OR Asia Medicines, East OR East Asia Medicine OR East Asia Medicines OR Medicines, East Asia OR Medicine, Oriental) | 33,897 |
| #1 | TS=(Diabetes Mellitus, Type 2 OR Diabetes Mellitus, Noninsulin-Dependent OR Diabetes Mellitus, Ketosis-Resistant OR Diabetes Mellitus, Ketosis Resistant OR Ketosis-Resistant Diabetes Mellitus OR Diabetes Mellitus, Non Insulin Dependent OR Diabetes Mellitus, Non-Insulin-Dependent OR Non-Insulin-Dependent Diabetes Mellitus OR Diabetes Mellitus, Stable OR Stable Diabetes Mellitus OR Diabetes Mellitus, Type II OR NIDDM OR Diabetes Mellitus, Noninsulin Dependent OR Diabetes Mellitus, Maturity-Onset OR Diabetes Mellitus, Maturity Onset OR Maturity-Onset Diabetes Mellitus OR Maturity Onset Diabetes Mellitus OR MODY OR Diabetes Mellitus, Slow-Onset OR Diabetes Mellitus, Slow Onset OR Slow-Onset Diabetes Mellitus OR Type 2 Diabetes Mellitus OR Noninsulin-Dependent Diabetes Mellitus OR Noninsulin Dependent Diabetes Mellitus OR Maturity-Onset Diabetes OR Diabetes, Maturity-Onset OR Maturity Onset Diabetes OR Type 2 Diabetes OR Diabetes, Type 2 OR Diabetes Mellitus, Adult-Onset OR Adult-Onset Diabetes Mellitus OR Diabetes Mellitus, Adult Onset) | 157,290 |


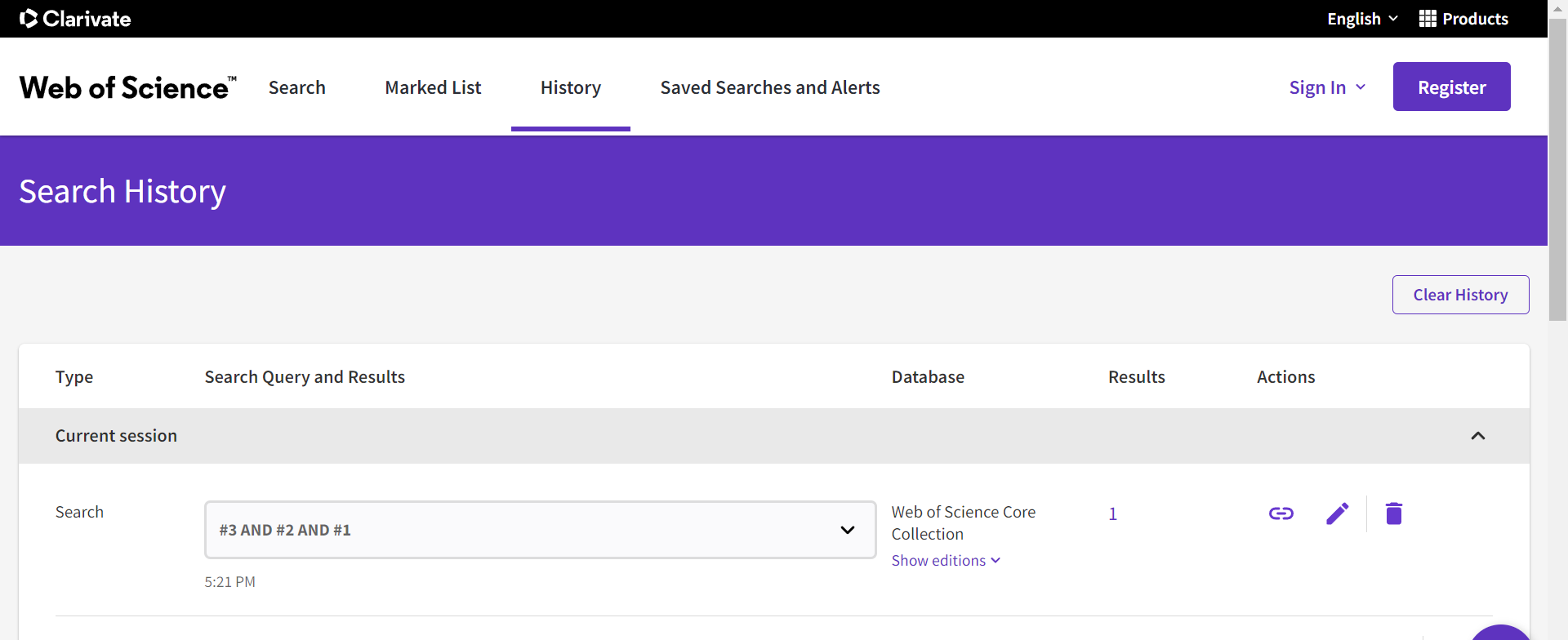


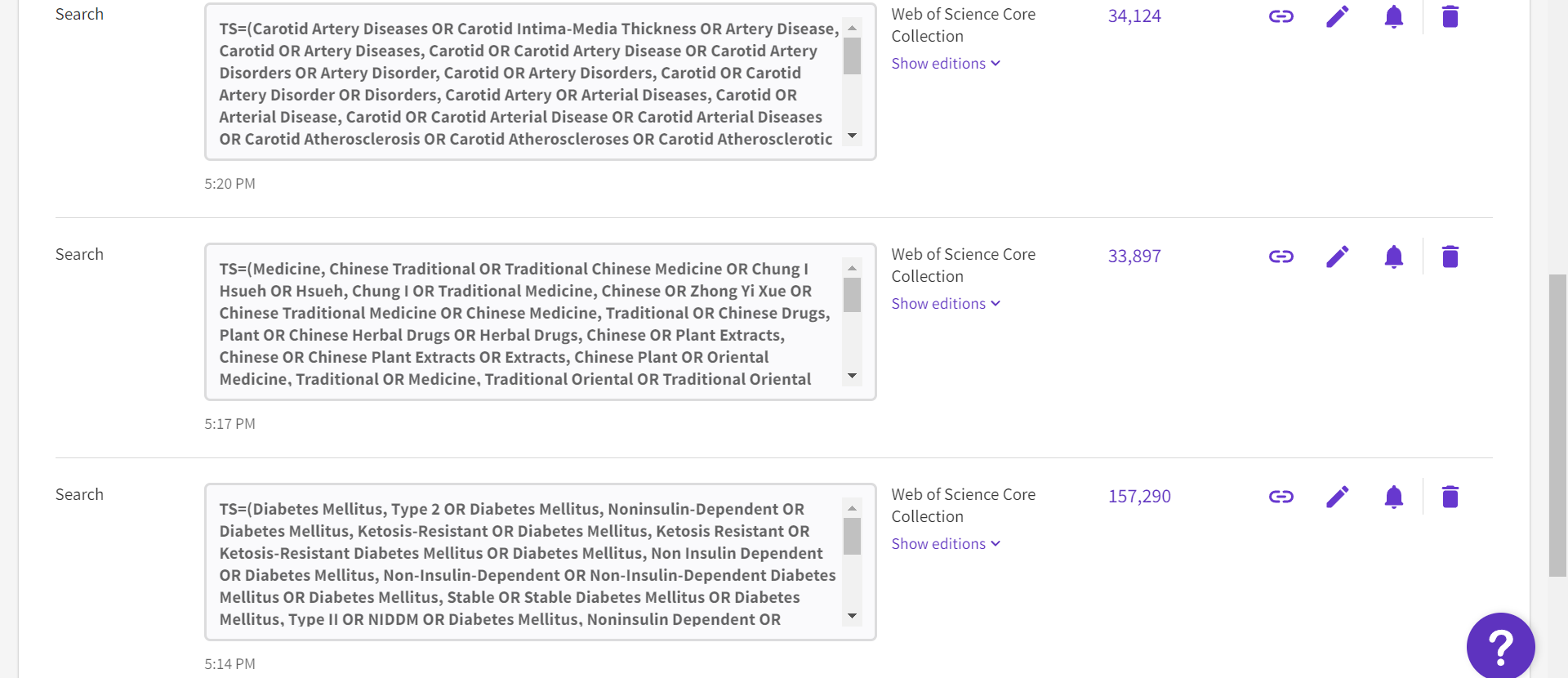


CNKI

The retrieval of the CNKI database was conducted on July 16, 2022, and a total of 376 records were retrieved.

(SU='2型糖尿病'+'T2DM'+'二型糖尿病'+'Ⅱ型糖尿病'+'糖尿病'+'DM'+'消渴'+'消瘅' OR TKA='2型糖尿病'+'T2DM'+'二型糖尿病'+'Ⅱ型糖尿病'+'糖尿病'+'DM'+'消渴'+'消瘅') AND (SU='颈动脉粥样硬化斑块'+'颈动脉粥样硬化'+'颈动脉粥样斑块'+'颈动脉硬化'+'颈动脉斑块'+'颈动脉增厚'+'颈动脉内中膜厚度'+'颈动脉内中膜增厚'+'颈动脉内膜中层厚度'+'颈动脉狭窄' OR TKA='颈动脉粥样硬化斑块'+'颈动脉粥样硬化'+'颈动脉粥样斑块'+'颈动脉硬化'+'颈动脉斑块'+'颈动脉增厚'+'颈动脉内中膜厚度'+'颈动脉内中膜增厚'+'颈动脉内膜中层厚度'+'颈动脉狭窄') AND (SU='中西医'+'中医药'+'中医'+'中药'+'中草药'+'草药'+'中成药'+'方剂' OR TKA='中西医'+'中医药'+'中医'+'中药'+'中草药'+'草药'+'中成药'+'方剂')


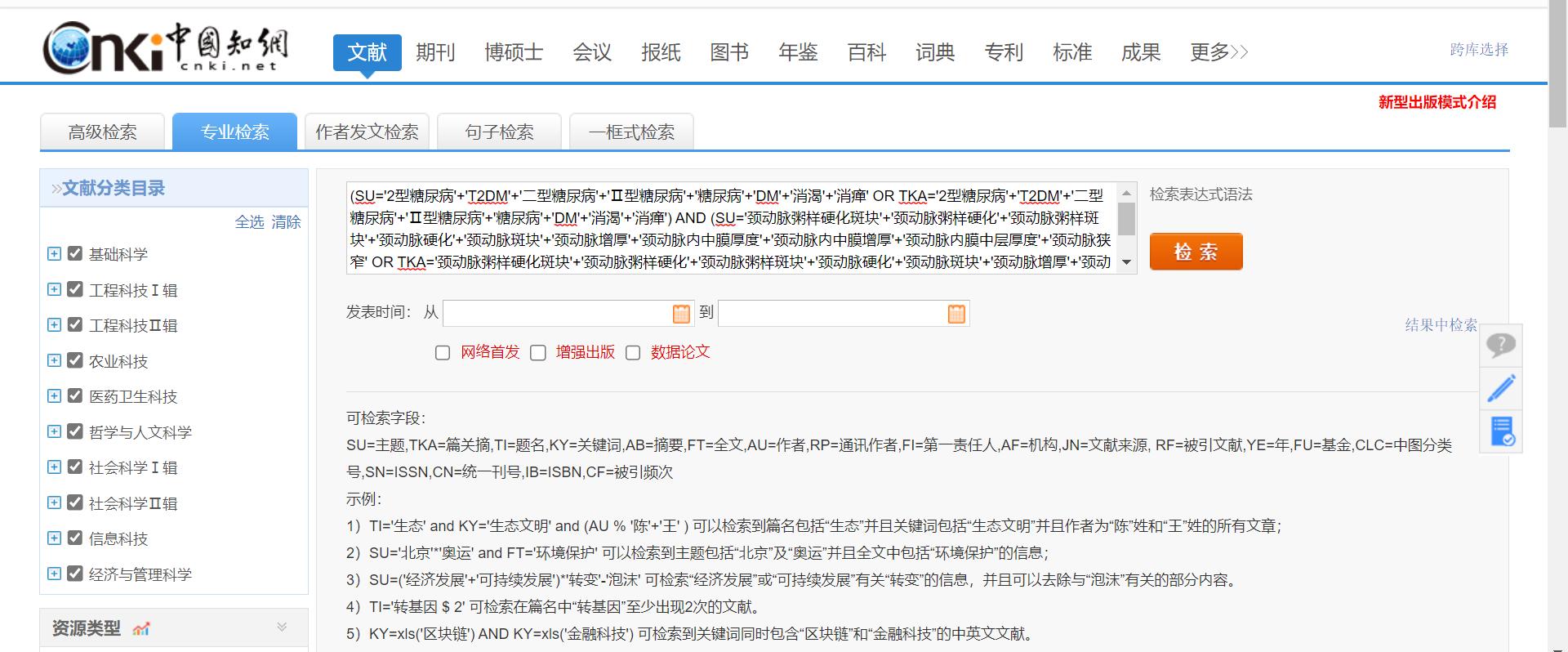


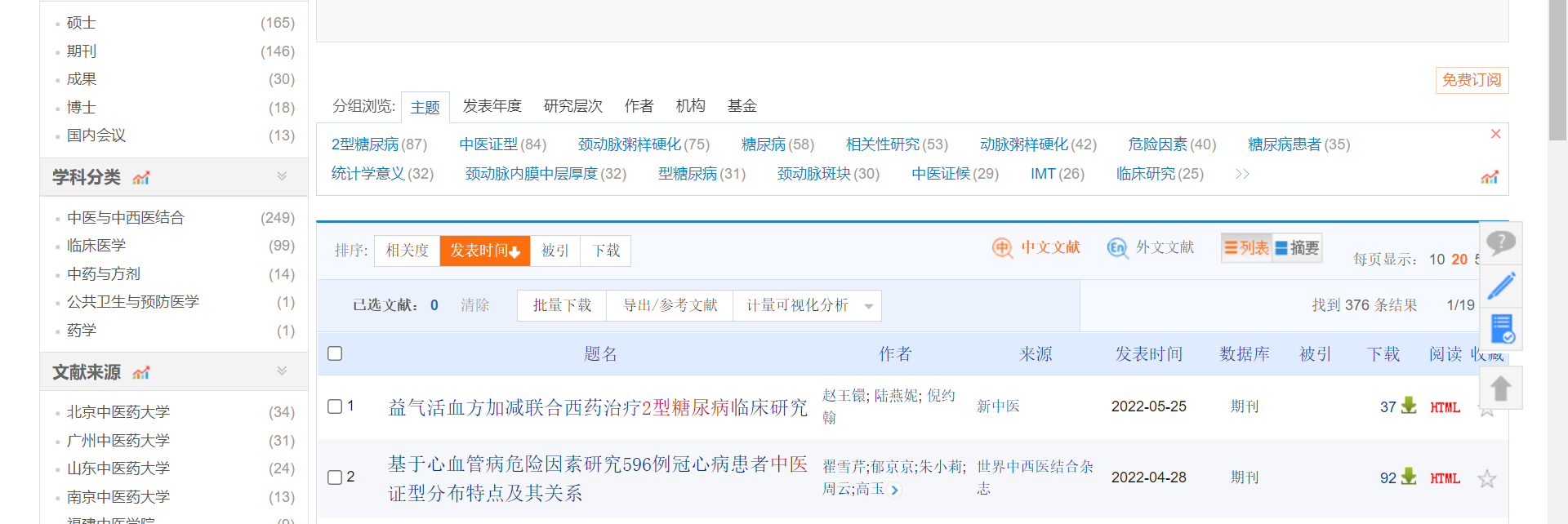


VIP

The retrieval of the VIP database was conducted on July 16, 2022, and a total of 159 records were retrieved.

((M=(2型糖尿病+T2DM+二型糖尿病+Ⅱ型糖尿病+糖尿病+DM+消渴+消瘅)) OR (R=(2型糖尿病+T2DM+二型糖尿病+Ⅱ型糖尿病+糖尿病+DM+消渴+消瘅))) AND ((M=(颈动脉粥样硬化斑块+颈动脉粥样硬化+颈动脉粥样斑块+颈动脉硬化+颈动脉斑块+颈动脉增厚+颈动脉内中膜厚度+颈动脉内中膜增厚+颈动脉内膜中层厚度+颈动脉狭窄)) OR (R=(颈动脉粥样硬化斑块+颈动脉粥样硬化+颈动脉粥样斑块+颈动脉硬化+颈动脉斑块+颈动脉增厚+颈动脉内中膜厚度+颈动脉内中膜增厚+颈动脉内膜中层厚度+颈动脉狭窄))) AND ((M=(中西医+中医药+中医+中药+中草药+草药+中成药+方剂)) OR (R=(中西医+中医药+中医+中药+中草药+草药+中成药+方剂)))


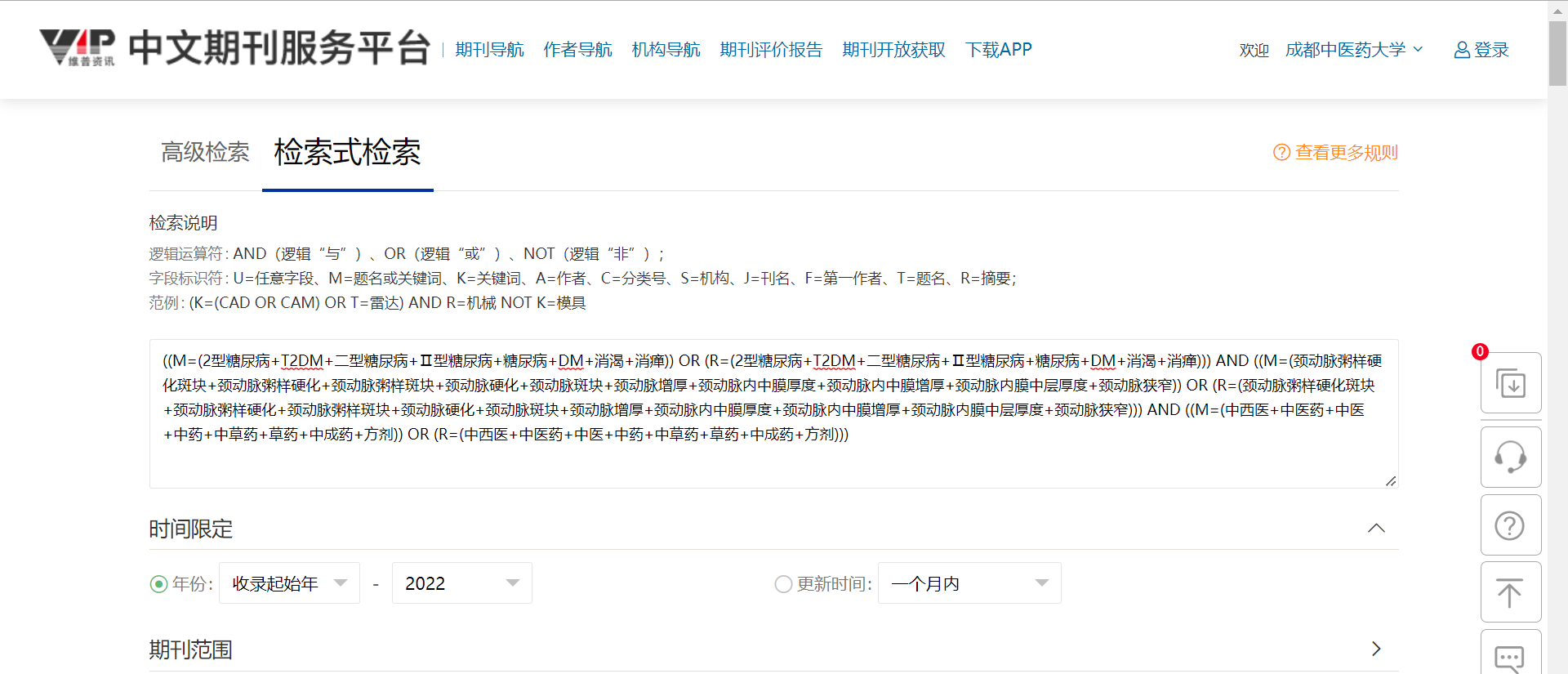


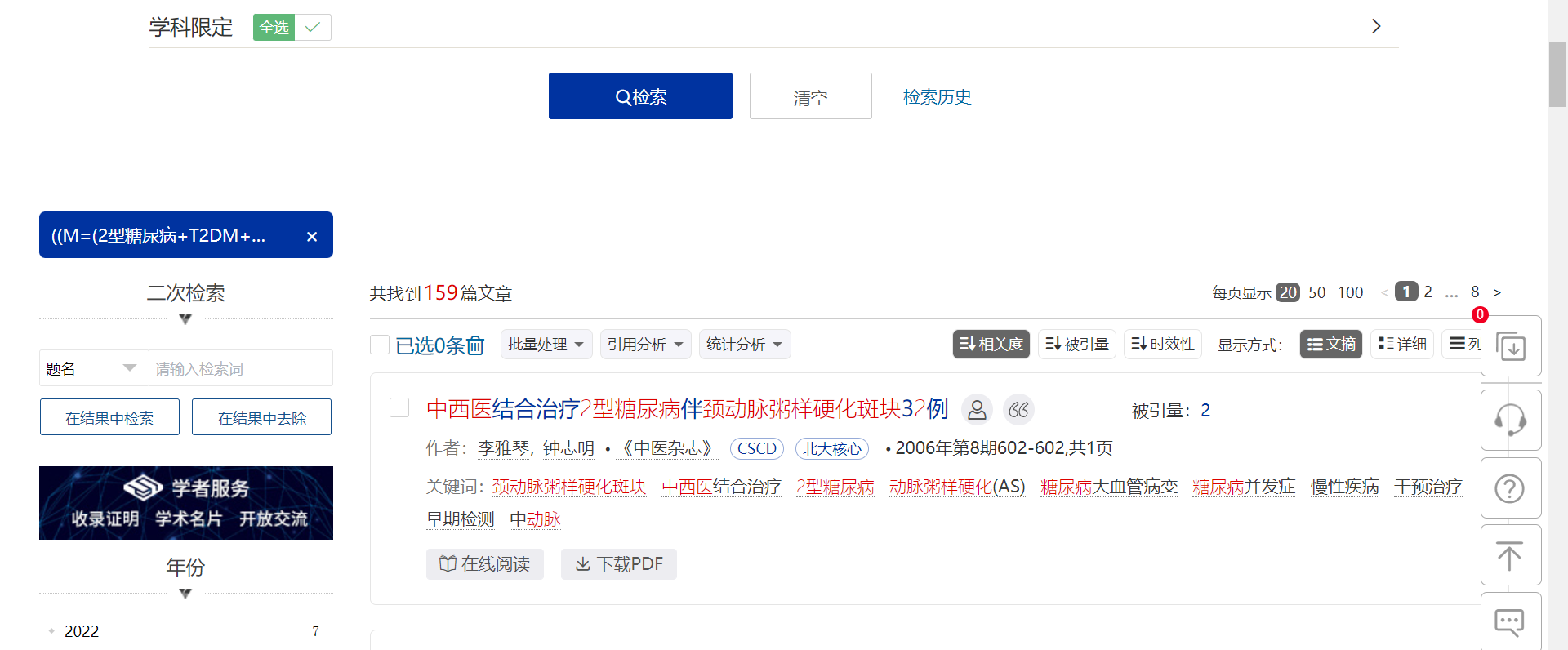


Wan Fang

The retrieval of the Wan Fang database was conducted on July 16, 2022, and a total of 359 records were retrieved.

(主题:(2型糖尿病 or T2DM or 二型糖尿病 or Ⅱ型糖尿病 or 糖尿病 or DM or 消渴 or 消瘅) or 题名或关键词:(2型糖尿病 or T2DM or 二型糖尿病 or Ⅱ型糖尿病 or 糖尿病 or DM or 消渴 or 消瘅)) and (主题:(颈动脉粥样硬化斑块 or 颈动脉粥样硬化 or 颈动脉粥样斑块 or 颈动脉硬化 or 颈动脉斑块 or 颈动脉增厚 or 颈动脉内中膜厚度 or 颈动脉内中膜增厚 or 颈动脉内膜中层厚度 or 颈动脉狭窄) or 题名或关键词:(颈动脉粥样硬化斑块 or 颈动脉粥样硬化 or 颈动脉粥样斑块 or 颈动脉硬化 or 颈动脉斑块 or 颈动脉增厚 or 颈动脉内中膜厚度 or 颈动脉内中膜增厚 or 颈动脉内膜中层厚度 or 颈动脉狭窄)) and (主题:(中西医 or 中医药 or 中医 or 中药 or 中草药 or 草药 or 中成药 or 方剂) or 题名或关键词:(中西医 or 中医药 or 中医 or 中药 or 中草药 or 草药 or 中成药 or 方剂))


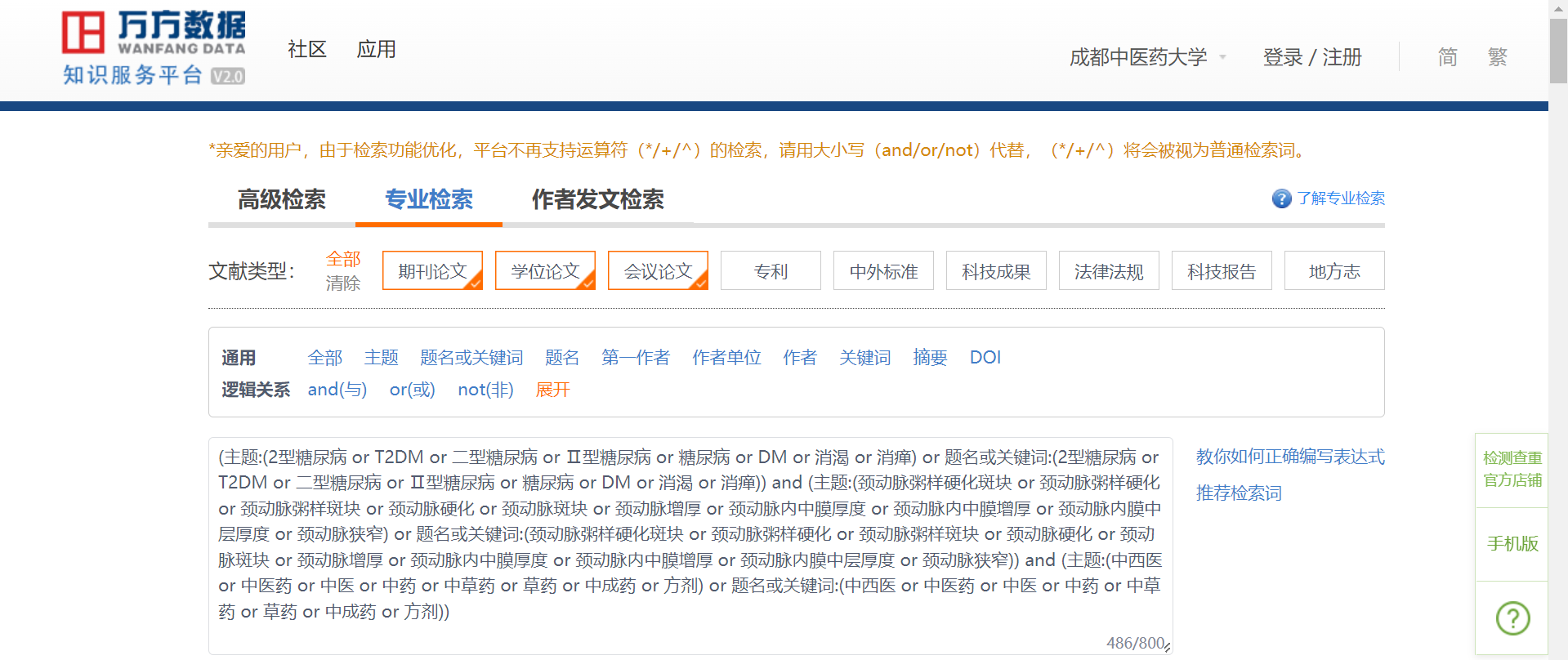


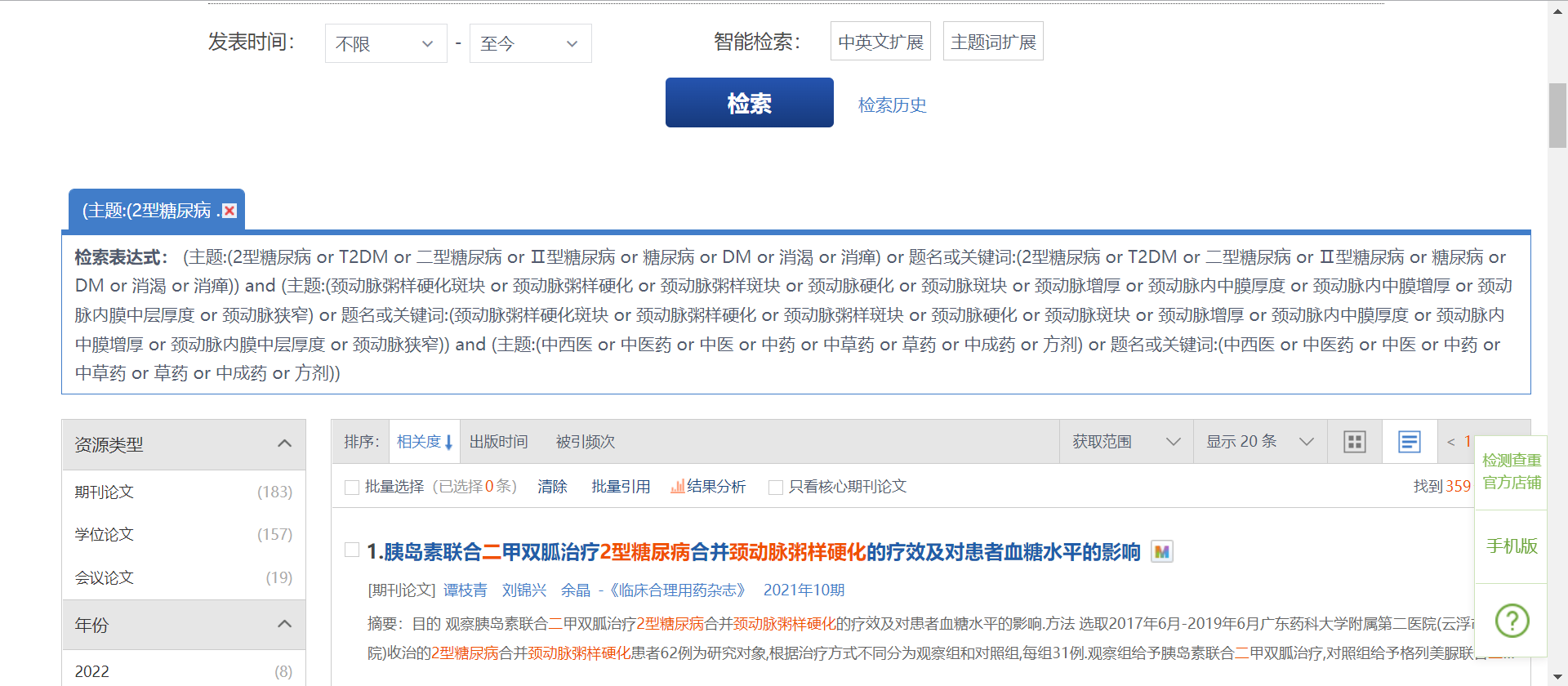


CBM

The retrieval of the CBM database was conducted on July 16, 2022, and a total of 182 records were retrieved.

("中西医"[常用字段:智能] OR "中医药"[常用字段:智能] OR "中医"[常用字段:智能] OR "中药"[常用字段:智能] OR "中草药"[常用字段:智能] OR "草药"[常用字段:智能] OR "中成药"[常用字段:智能] OR "方剂"[常用字段:智能]) AND ("颈动脉粥样硬化斑块"[常用字段:智能] OR "颈动脉粥样硬化"[常用字段:智能] OR "颈动脉粥样斑块"[常用字段:智能] OR "颈动脉硬化"[常用字段:智能] OR "颈动脉斑块"[常用字段:智能] OR "颈动脉增厚"[常用字段:智能] OR "颈动脉内中膜厚度"[常用字段:智能] OR "颈动脉内中膜增厚"[常用字段:智能] OR "颈动脉内膜中层厚度"[常用字段:智能] OR "颈动脉狭窄"[常用字段:智能]) AND ("2型糖尿病"[常用字段:智能] OR "T2DM"[常用字段:智能] OR "二型糖尿病"[常用字段:智能] OR "Ⅱ型糖尿病"[常用字段:智能] OR "糖尿病"[常用字段:智能] OR "DM"[常用字段:智能] OR "消渴"[常用字段:智能] OR "消瘅"[常用字段:智能])


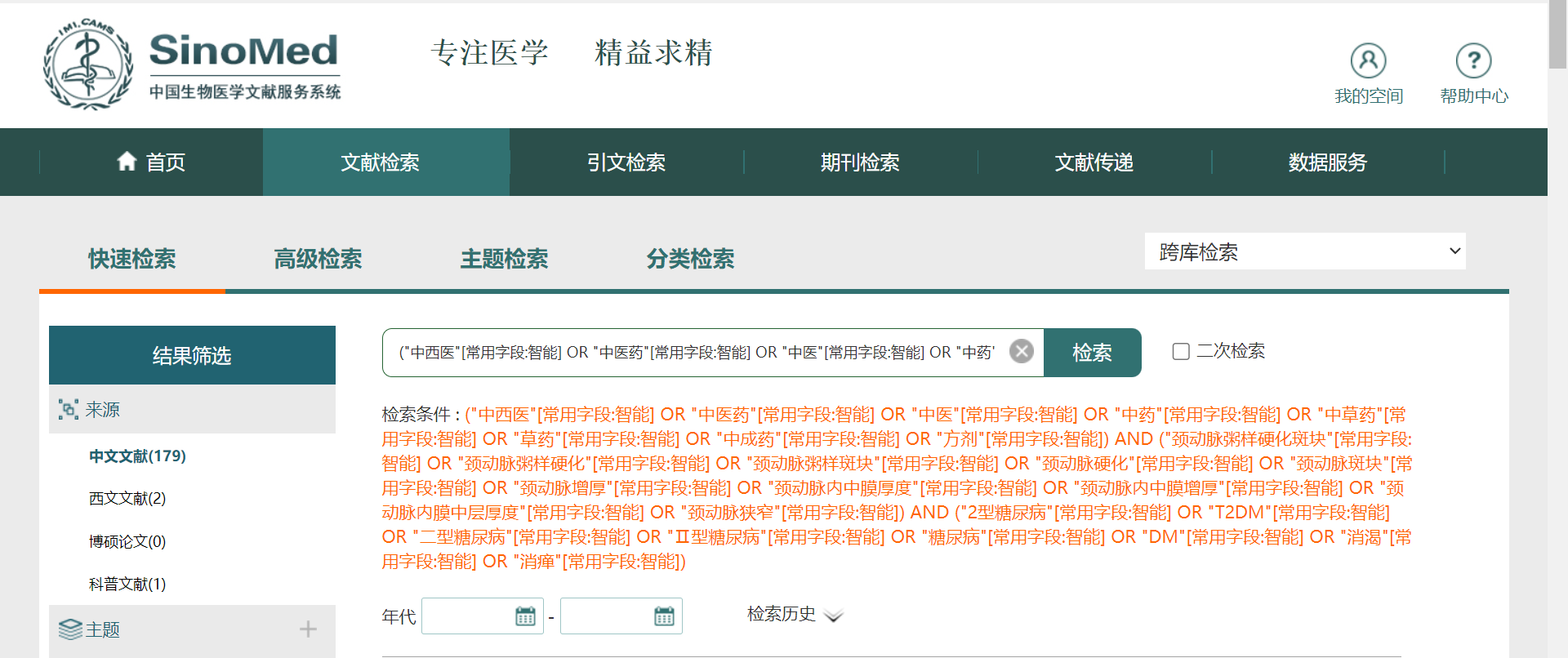


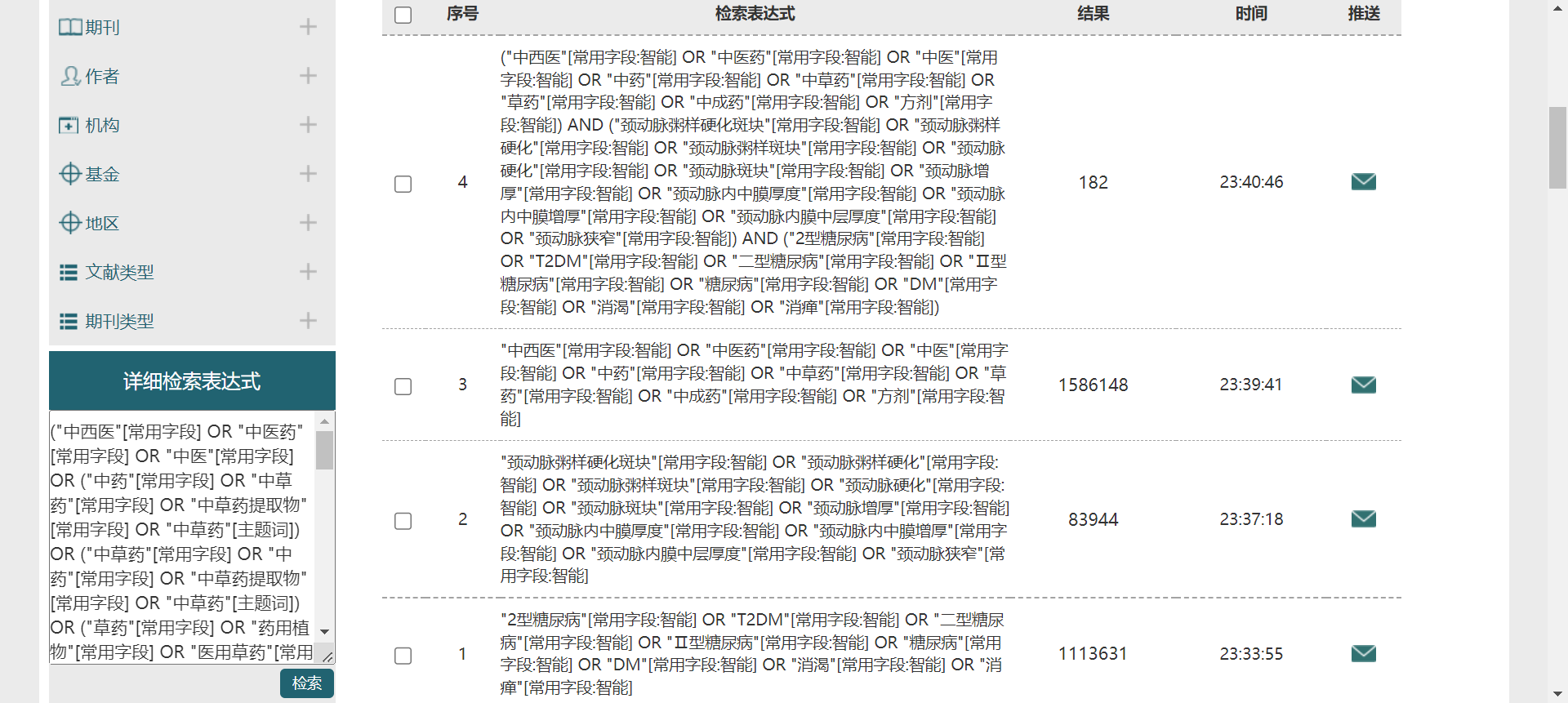


ClinicalTrials.gov

The retrieval of the ClinicalTrials.gov database was conducted on July 16, 2022, and no records were retrieved.


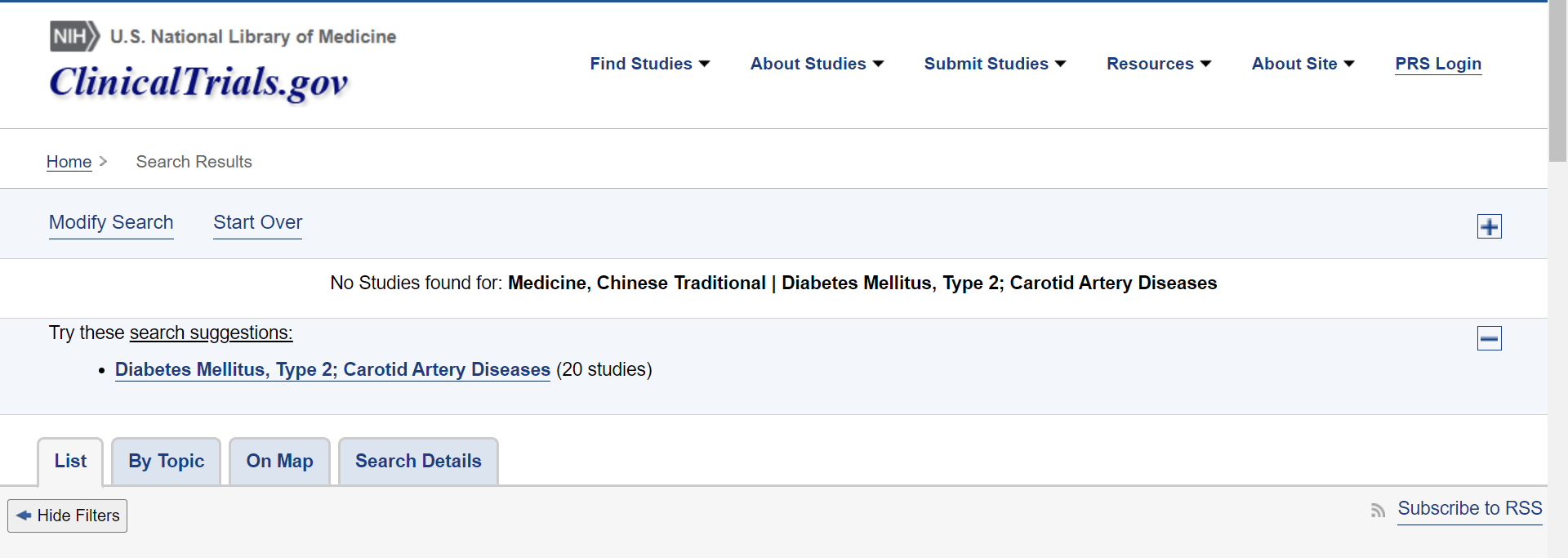


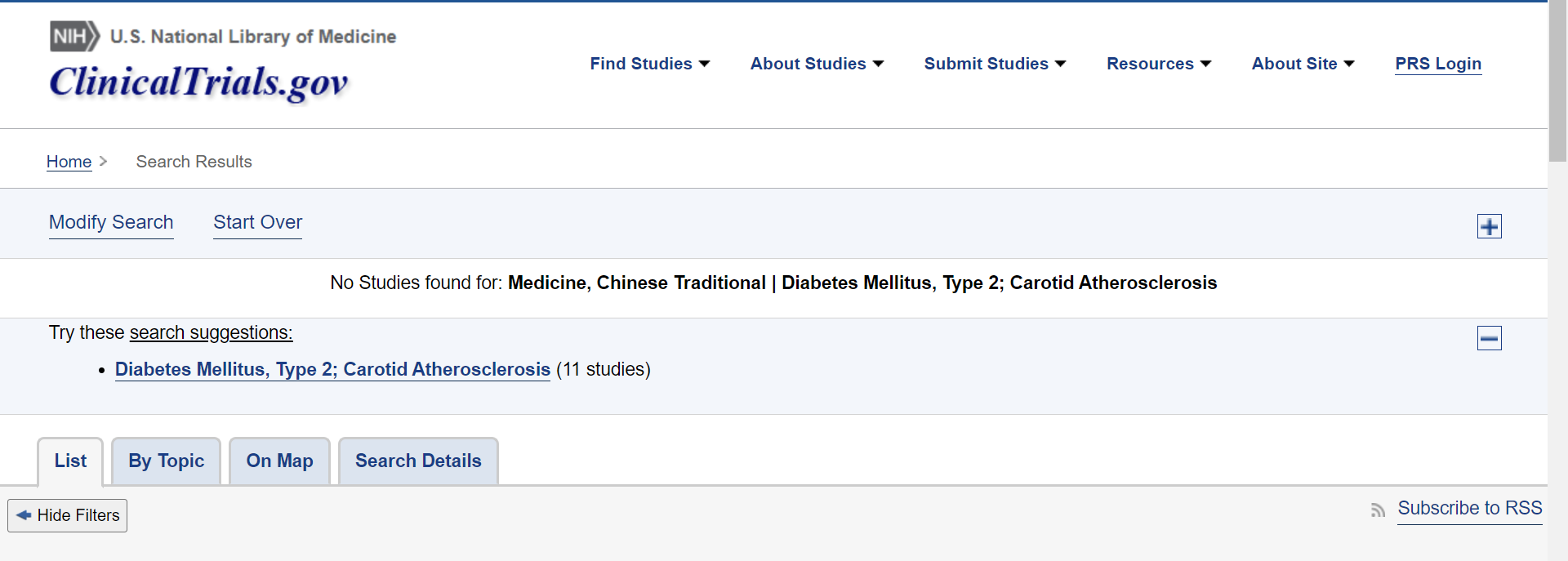


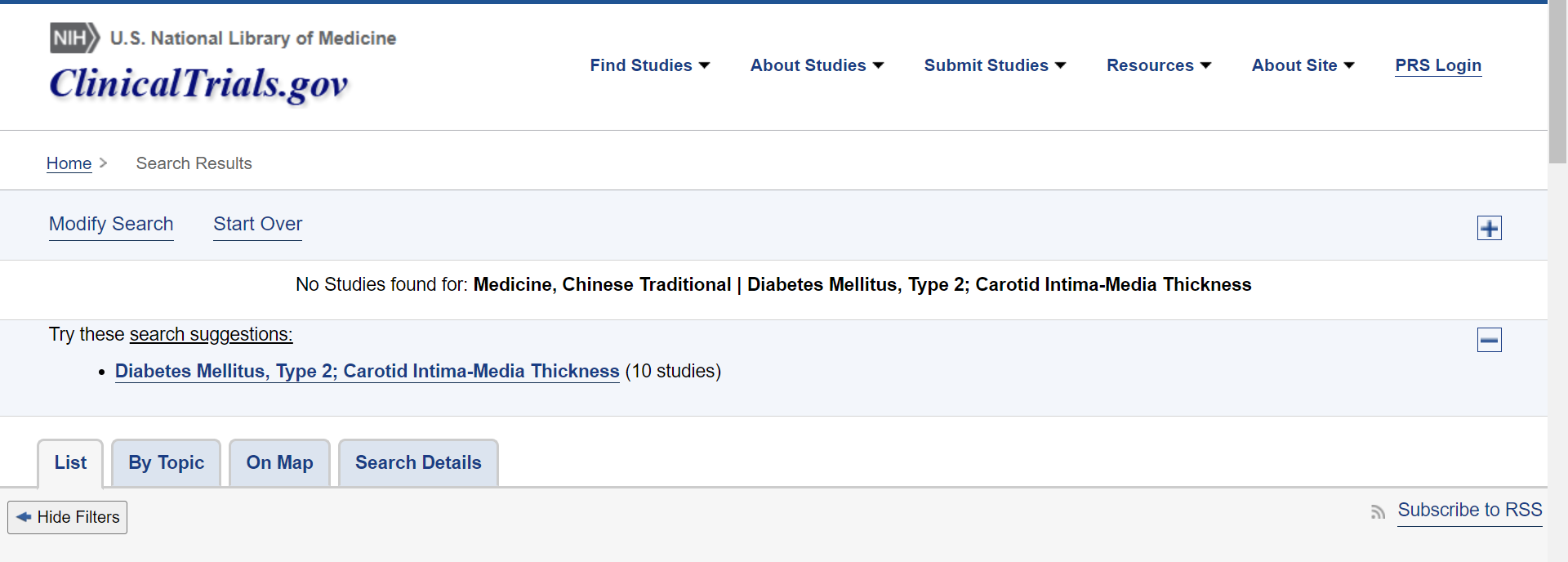


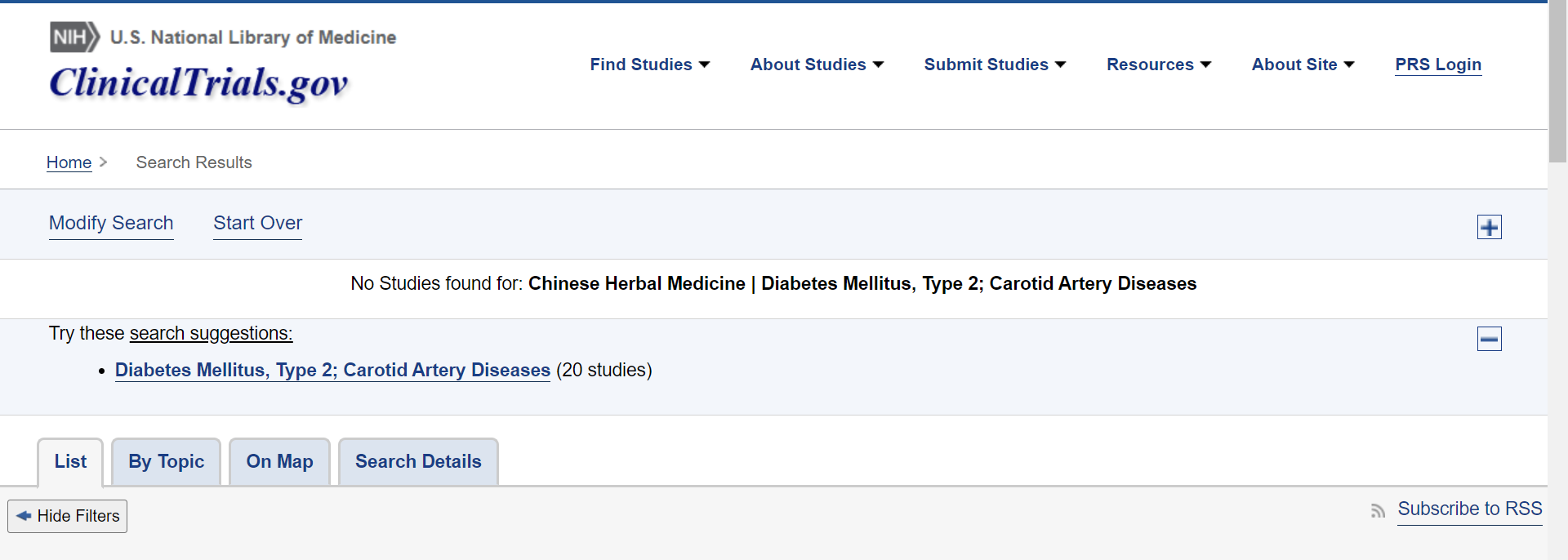


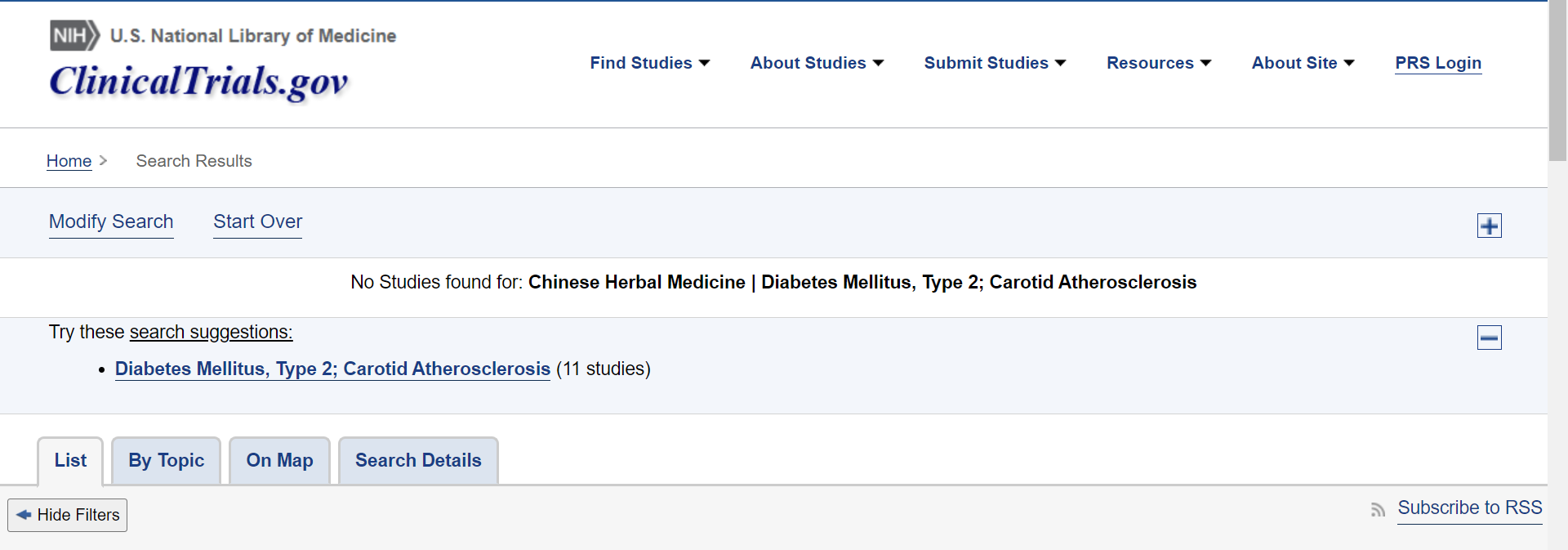


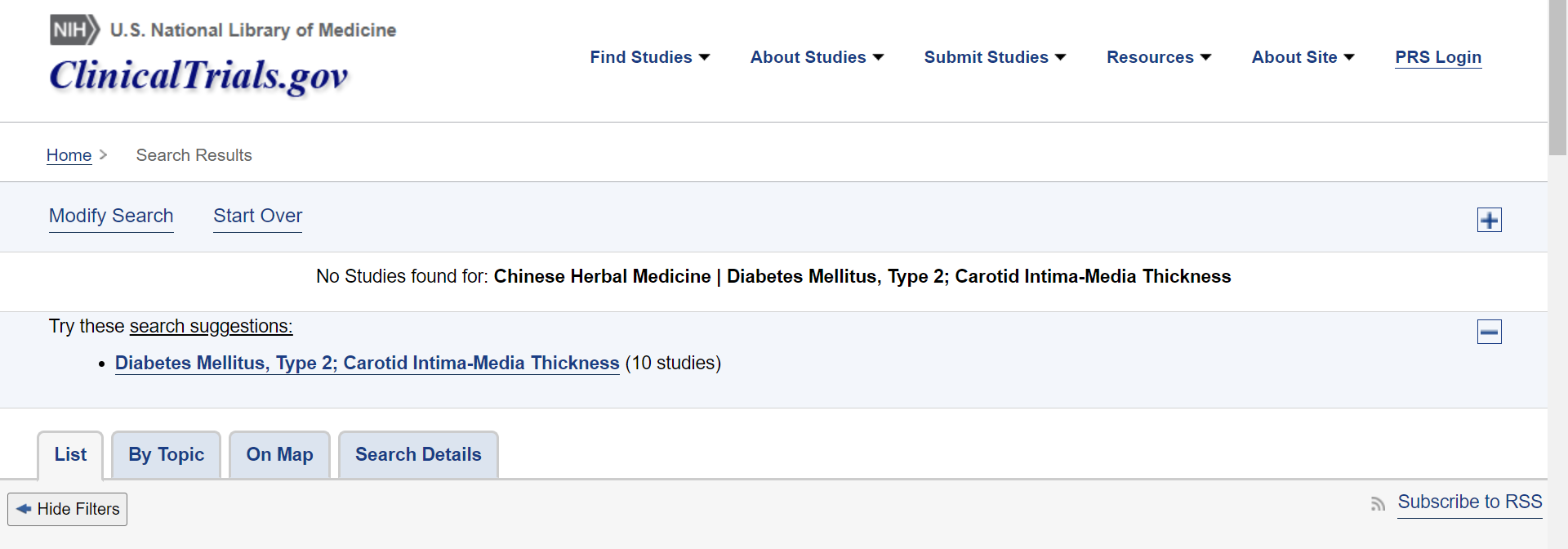


Chinese Clinical Trial Registry

The retrieval of the Chinese Clinical Trial Registry was conducted on July 16, 2022, and no records were retrieved.


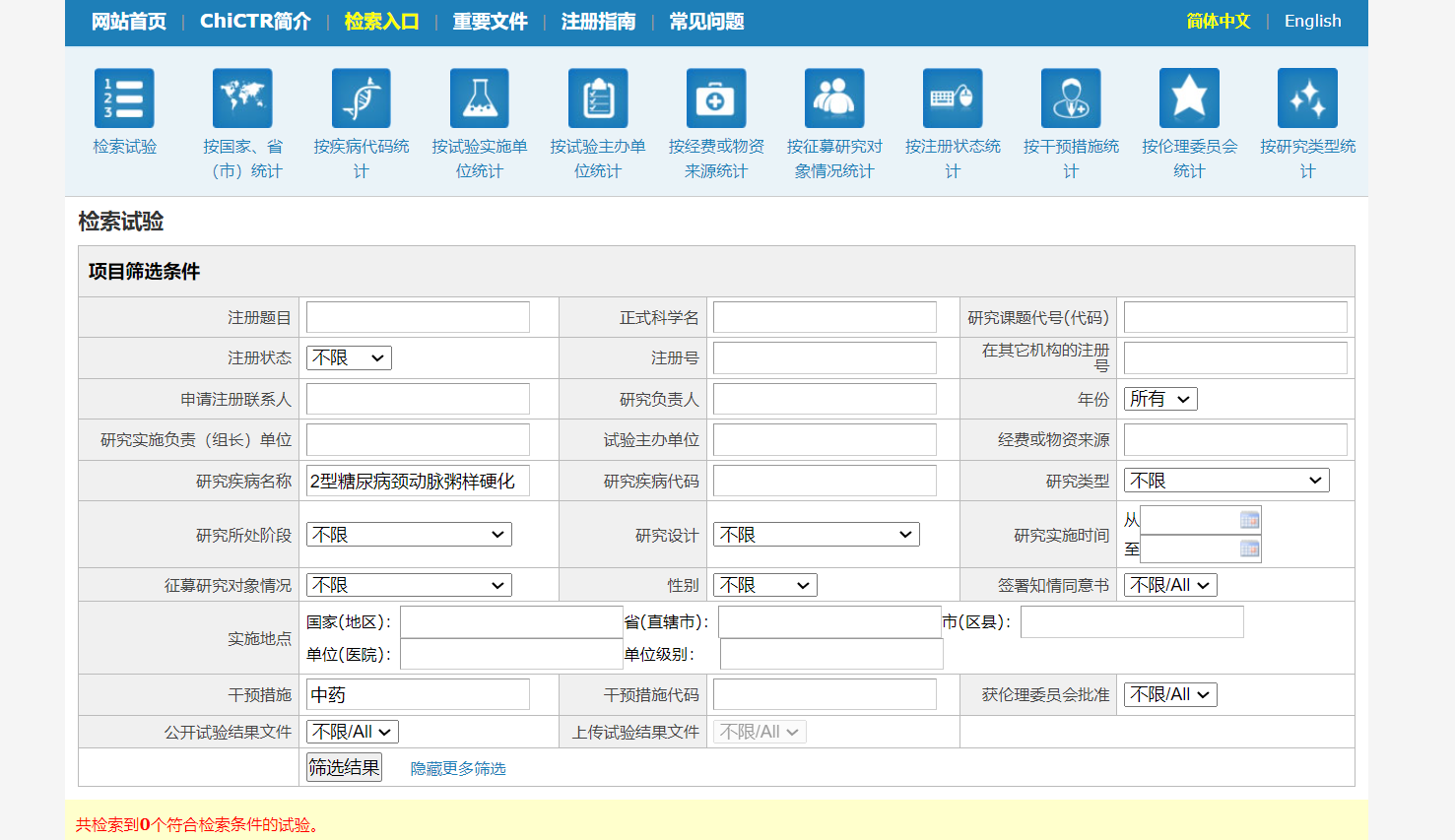


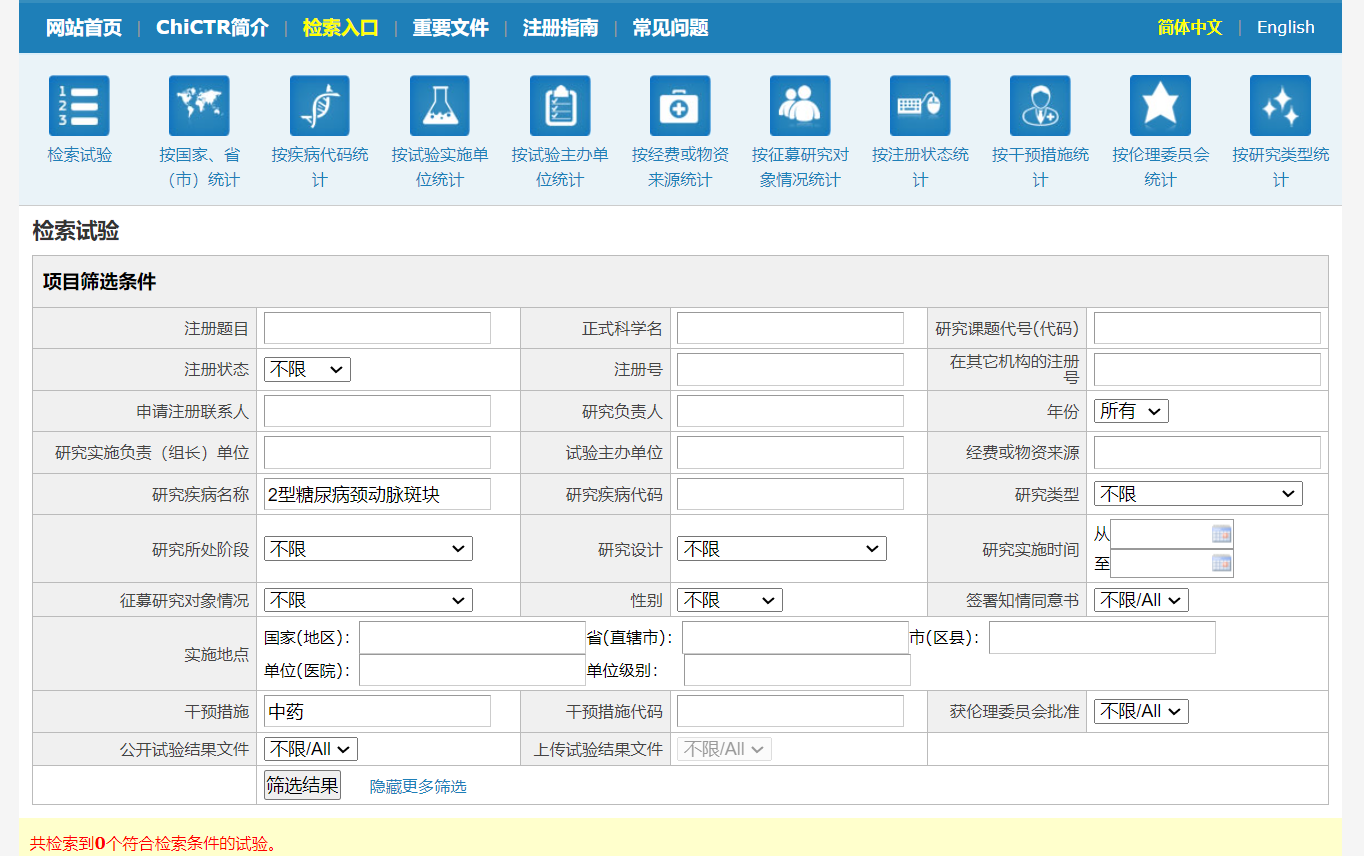


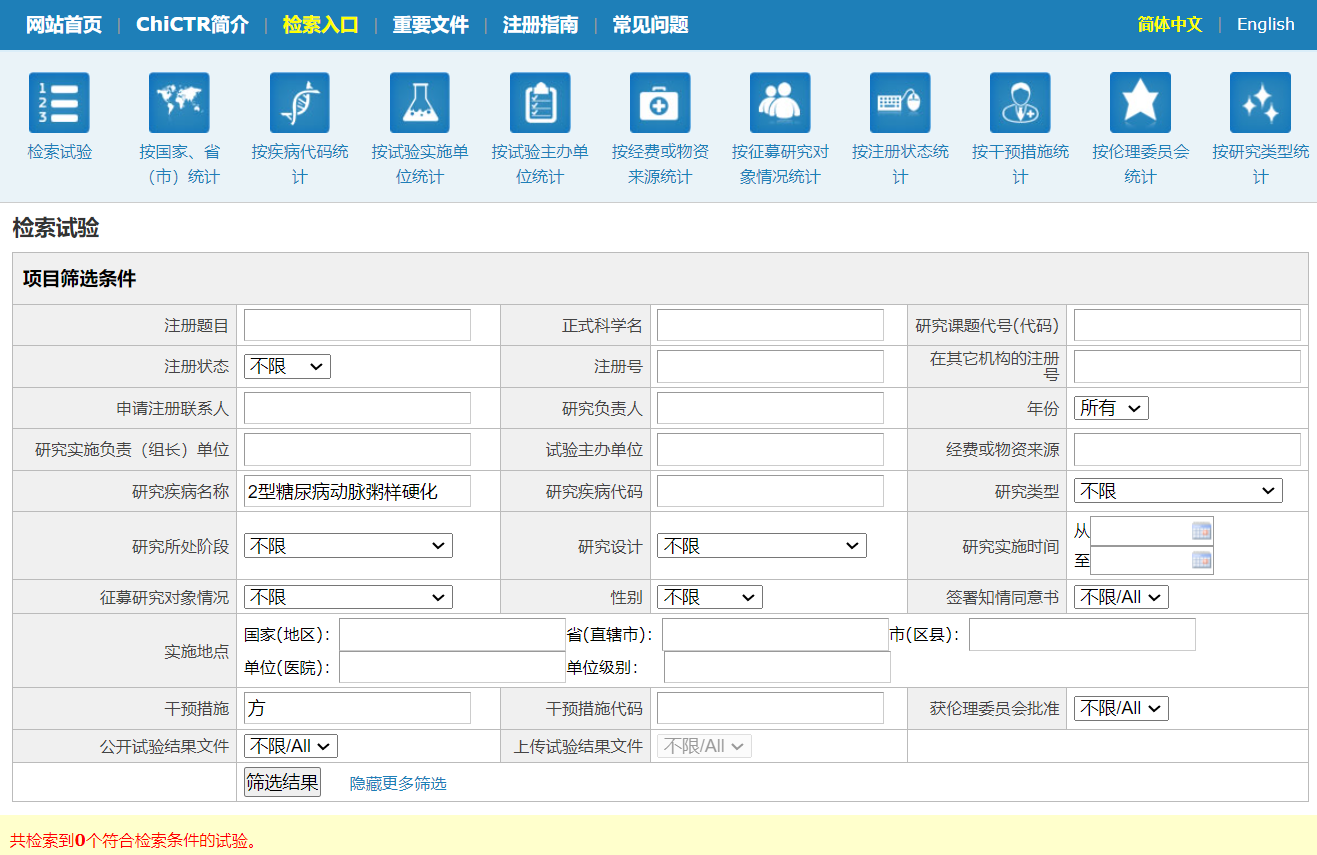


# Supplementary Material S3. Literature excluded after reading the full text and reasons

**1) The study design was not a randomized controlled trial:**

[1] Han, J.H., Li, Z., and Yang, H.Y. (2010). The Effect of Ruanjian Xiaoji Huoxue Huatan Compound on Inflammatory Activity of Type 2 Diabetes Mellitus Complicated with Carotid Atherosclerotic Plaque. *J Sichuan Tradit Chin Med.* 28(2), 75-76. (The subjects in this study were not randomly assigned to each group.)

[2] Kong, W.W., Wu, X.S., and Yu, J.Y. (2011). Liuwei Dihuang Pill Combined with Ginkgo Leaf Intervention Influencing Type Ⅱ Diabetes Early Aorta Lesion and Inflammation Factor. *J Zhejiang Chin Med Univ.* 35(6), 846-848. doi: 10.16466/j.issn1005-5509.2011.06.028. (The subjects in this study were not randomly assigned to each group.)

[3] He, C.L. (2013). Clinical Research of Zengye Chengqi Decoction on Carotid Artery Intima-Media Thickness and Lipid of Type 2 Diabetes Artery Atherosclerosis. *Hebei J Tradit Chin Med.* 35(3), 370-372. (The study was a single-arm trial with no control group.)

[4] Yin, X.P. (2013). 72 Cases of Type 2 Diabetic Macrovascular Disease Treated by Replenishing Qi and Activating Blood. *J Front Med.* (2), 299-300. (The study was a single-arm trial with no control group.)

[5] Li, L., Jiang, W.H., Li, L.H., Zhai, M.X., Li, H., and Fang, Y.H. (2014). Clinical Observation on the Treatment of Type 2 Diabetes Mellitus Complicated with Carotid Atherosclerosis by Combination of Traditional Chinese and Western Medicine. *Chin J Clin Ration Drug Use.* 7(16), 60-61. doi: 10.15887/j.cnki.13-1389/r.2014.16.077. (The study was a single-arm trial with no control group.)

[6] Cheng, X.Q. (2016). *Effects of Traditional Chinese Medicine Intervention on the Dynamic Changes of Carotid Intima-Media Thickness in 22 Patients with Type 2 Diabetes Mellitus*. [Master's thesis]. Beijing: Beijing University of Chinese Medicine. (This was a retrospective study.)

[7] Xu, J.M., Wang, M., and Yu, S.J. (2018). Experimental and Clinical Evidence of Mudan Granule (Tangmoning) in Treatment of Diabetes Mellitus and Various Complications. *Chin Arch Tradit Chin Med.* 36(2), 384-387. doi: 10.13193/j.issn.1673-7717.2018.02.034. (This was a review.)

[8] Su, B.Y., Zhang, L.S., Tian, C.F., Lu, C.P., and Qu, Z. (2016). Effect of Xuezhikang Capsule on Carotid Intima Thickness in Type 2 Diabetic Patients. Hebei Med J. 38(16), 2453-2455. (This literature was grouped according to the wishes of the investigators and was not a randomized controlled trial.)

**2) The study subjects did not have type 2 diabetes mellitus with carotid atherosclerosis:**

[1] Li, Y.Q., and Zhong, Z.M. (2006). 32 Cases of Type 2 Diabetes Mellitus with Carotid Atherosclerotic Plaque Treated by Integrated Traditional Chinese and Western Medicine. *J Tradit Chin Med.* (8), 602. doi: 10.13288/j.11-2166/r.2006.08.028. (The subjects had only type 2 diabetes mellitus and no carotid atherosclerosis, only atherosclerotic indicators are involved.)

[2] Yang, G.Z. (2007). The Study of the Effect of Tongxinluo on the Atherosclerosis in Patients with Type 2 Diabetes Mellitus by Color Doppler Flow Imaging. [Master's thesis]. Qingdao: Qingdao University. (The site of atherosclerosis in the subjects was unclear.)

[3] Kong, W.W. (2008). *Effects of Traditional Chinese Medicine on Macrovascular Disease and Inflammation Factors in Patients with Type 2 Diabetes Mellitus in Early Stage and Perform Economic Health Evaluation*. [Master's thesis]. Nanjing: Nanjing University of Chinese Medicine. (The subjects had only type 2 diabetes mellitus and no carotid atherosclerosis.)

[4] Shao, X.L. (2008). *Effects of Traditional Chinese Medicine on Macrovascular Disease and the Status of Oxidative Stress in Patients with Type 2 Diabetes Mellitus in Early Stage*. [Master's thesis]. Nanjing: Nanjing University of Chinese Medicine. (The subjects had only type 2 diabetes mellitus and no carotid atherosclerosis.)

[5] Zhang, H.J., and Yang, Y. (2010). Therapeutic Effect of Tongxinluo Capsule Combined with Prostaglandin E1 on Diabetic Atherosclerosis. *Chin J Diffic Compl Cas.* 9(4), 273-275. (The subjects had only type 2 diabetes mellitus and no carotid atherosclerosis.)

[6] Li, K.J., Lei, Y.H., and Liu, X.H. (2013). Clinical Observation of 33 Cases of Type 2 Diabetes Mellitus Complicated with Atherosclerotic Lesions Treated by Integrated Traditional Chinese and Western Medicine. *Guid J Tradit Chin Med Pharm.* 19(10), 53-54. doi: 10.13862/j.cnki.cn43-1446/r.2013.10.049. (The subjects had only type 2 diabetes mellitus and no carotid atherosclerosis.)

[7] Liu, C.P., and Wang, H.S. (2015). 60 Cases of Type 2 Diabetes Mellitus with Atherosclerosis Treated by Strengthening Spleen and Replenishing Kidney. *Fujian J Tradit Chin Med.* 46(4), 22-23. doi: 10.13260/j.cnki.jfjtcm.010939. (The subjects had only type 2 diabetes mellitus and no carotid atherosclerosis.)

[8] Pan, Y.Y., Zhao, P., and Zhang, X.Q. (2015). Ultrasound Evaluation of Jiangtang Sanhuang Tablet in the Treatment of Carotid Atherosclerosis in Diabetic Patients with Stasis-Heat Syndrome. *New Chin Med.* 47(8), 95-97. doi: 10.13457/j.cnki.jncm.2015.08.044. (The subjects had only type 2 diabetes mellitus and no carotid atherosclerosis.)

[9] Ran, C.M. (2015). The Therapeutic Effect of Integrated Traditional Chinese and Western Medicine on Diabetic Atherosclerosis. *Cardiovasc Dis Electron J Integr Tradit Chin West Med.* 3(9), 110-111. doi: 10.16282/j.cnki.cn11-9336/r.2015.09.066. (The site of atherosclerosis in the subjects was unclear.)

[10] Yan, Z.S., Jiang, X.Q., Cui, H.F., Li, A.F., and Wang, C.X. (2015). The Effect of Dangua Decoction on Plaque Stability in Type 2 Diabetes with Atherosclerosis Patients. *Med Equip.* 28(10), 128-129. (The site of atherosclerosis in the subjects was unclear.)

[11] Cheng, S.H., Wang, J., Xu, G.H., Sun, G.X., Tao, X.J., Yang, X.C., et al. (2016). Effect of Naoxintong Capsule on Vascular Remodeling in Type 2 Diabetic Patients with Subclinical Vascular Disease. *Chin J Integr Tradit West Med.* 36(12), 1439-1444. (The site of atherosclerosis in the subjects may not be in the carotid artery.)

[12] Zhao, Y., Yu, J., Liu, J., and An, X. (2016). The Role of Liuwei Dihuang Pills and Ginkgo Leaf Tablets in Treating Diabetic Complications. *Evid Based Complement Alternat Med.* 2016, 7931314. doi: 10.1155/2016/7931314. (The subjects had only type 2 diabetes mellitus and no carotid atherosclerosis.)

[13] Xia, M.L., Liu, H.Z., Xu, Q.H., Zhang, L., Hu, J.J., and Hu, X.Y. (2016). Clinical Effect of Jianpi Xiaozhi Prescription on Serum Levels of Matrix Metallopeptidase-9 and Interleukin-1β in Patients with Type 2 Diabetes Mellitus Complicated by Macroangiopathy. *J Anhui Univ Chin Med.* 35(6), 37-41. (The site of atherosclerosis in the subjects may not be in the carotid artery.)

[14] Xu, Y.Z., and Wei, X. (2017). Effect of Nourishing Yin and Activating Blood Chinese Medicine Combined with Intensive Insulin on Secondary Macrovascular Disease of Type 2 Diabetes Mellitus and Its Influence on Inflammatory Cytokines. *Mod J Integr Tradit Chin West Med.* 26(11), 1198-1200. (The subjects were patients with diabetic macrovascular disease, not necessarily carotid atherosclerosis.)

[15] Dong, K.L. (2018). Effect of Danzhi Jiangtang Capsule on Vascular Remodeling of Type 2 Diabetic Patients with Subclinical Vascular Disease. *Acta Chin Med.* 33(9), 1644-1647. doi: 10.16368/j.issn.1674-8999.2018.09.389. (The site of atherosclerosis in the subjects may not be in the carotid artery.)

[16] He, T., Yi, G.W., and Xu, L.W. (2019). Effects of Liraglutide Combined with Shenqi Jiangtang Granule on Blood Glucose and Vascular Remodeling in Patients with Type 2 Diabetes Mellitus with Subclinical Macroangiopathy with Poor Insulin Control. *Mod J Integr Tradit Chin West Med.* 28(16), 1736-1740. (The site of atherosclerosis in the subjects may not be in the carotid artery.)

[17] Li, L.L. (2019). *To Evaluate the Effect of Shenqi Compound Sequential Therapy on Preventing Macrovascular Injury in Type 2 Diabetes From the Perspective of Hemorheology and CIMT*. [Master's thesis]. Chengdu: Chengdu University of Traditional Chinese Medicine. (The subjects had only type 2 diabetes mellitus and no carotid atherosclerosis.)

[18] Liu, Y.D., Ni, W.Y., and Cai, X.T. (2019). Efficacy of the Ziyin Huoxue Medicine on Type 2 Diabetes with Secondary Macroangiopathy and Glucose,Coagulation Index. *Clin J Chin Med.* 11(31), 76-78. (The subjects were patients with diabetic macrovascular disease, not necessarily carotid atherosclerosis.)

[19] Lu, T.G. (2019). Clinical Observation of Yishen Huoxue Huatan Decoction in the Treatment of Type 2 Diabetes Mellitus Complicated with Carotid Atherosclerosis. *Guangming J Chin Med.* 34(9), 1307-1309. (The site of atherosclerosis in the subjects may not be in the carotid artery.)

[20] Jia, C.X., Lyu, S.Q., and Wang, Z.Q. (2020). The Effect of Furong Tongmai Capsule on Blood Glucose, Inflammatory Indexes and Carotid Plaque in Patients with Diabetic Macrovascular Disease. *Mod J Integr Tradit Chin West Med.* 29(5), 527-530. (The subjects were patients with diabetic macrovascular disease, not necessarily carotid atherosclerosis.)

[21] Ma, X., and Zhou, S.H. (2021). Clinical Effect of Traditional Chinese Medicine Tongxinluo Capsules for Type 2 Diabetes Mellitus with Atherosclerosis. *Int Med Health Guid News.* 27(16), 2551-2553. (The site of atherosclerosis in the subjects was unclear.)

[22] Yuan, B.H. (2021). Clinical Efficacy and Safety of Compound Danshen Dripping Pills Combined with Atorvastatin Calcium in the Treatment of Type 2 Diabetes Mellitus Complicated with Carotid Atherosclerosis. *Doct.* 6(4), 43-45. (The subjects had only type 2 diabetes mellitus and no carotid atherosclerosis.)

[23] Liu, H.J., Chen, F., Fan, G.J., and Yang, F.M. (2022). Clinical Observation of Yiqi Tongluo Qingre Cream in Treatment of Elderly Patients with Type 2 Diabetes and Atherosclerosis. *Geriatr Health Care.* 28(1), 148-153. (The site of atherosclerosis in the subjects was unclear.)

**3) The treatment in the experimental group was Chinese herbal medicine alone, rather than Chinese herbal medicine combined with Western medicine:**

[1] Cai, J., Wang, Z.M., Gong, M.W., and Li, B.H. (2009). Yikangling Mixture for Carotid Intima-Media Thickening in Patients with Type 2 Diabetes Mellitus. *Hubei J Tradit Chin Med.* 31(1), 31-32.

[2] Fan, X.D. (2010). Clinical Observation of Xiaoban Tongmai Decoction in Treating 40 Cases of Diabetic Carotid Atherosclerotic Plaque. *China Foreign Med J.* (3).

[3] Xu, X.W., and Yu, W.N. (2010). The Effect of Xuezhikang on Serum Visfatin in Patients with Type 2 Diabetes and Carotid Artery Plaque. *Acta Chin Med.* 25(1), 125-126+130. doi: 10.16368/j.issn.1674-8999.2010.01.046.

[4] Wang, D.L. (2012). *Yiqi Huoxue Clinical Observation of Treatment of Diabetic Carotid Atherosclerotic Plaque*. [Master's thesis]. Shanghai: Shanghai University of Traditional Chinese Medicine.

[5] Zhang, M.X., Wang, H.Q., and Hu, Z.Y. (2014). The Effect of Tonifying Kidney and Replenishing Qi and Activating Blood on Carotid Atherosclerosis and Endothelial Function in Elderly Type 2 Diabetes Mellitus. *Acta Chin Med Pharmacol.* 42(5), 138-140. doi: 10.19664/j.cnki.1002-2392.2014.05.046.

[6] Wang, Z. (2017). *The Efficacy Evaluation of JianPi Xiaoke on Type 2 Diabetes Mellitus Patients with Subclinical Atherosclerosis and the Effects of the Expression of P38 MAPK in Rats of Diabetic Atherosclerosis*. [Master's thesis]. Jinan: Shandong University of Traditional Chinese Medicine.

[7] Lu, Z., Zhang, F., and Yan, Y. (2022). Clinical Observation of Lianzhu Xiaoke Granule in the Treatment of Type 2 Diabetes Mellitus Complicated with Carotid Atherosclerotic Plaque. Yunnan J Tradit Chin Med Mater Med. 43(2), 96-99. doi: 10.16254/j.cnki.53-1120/r.2022.02.026.

[8] Xu, X.J., Liu, D., Zhang, W.N., and Tang, X.Z. (2015). Clinical Observation of Self-Made Strengthening Spleen and Removing Blood Stasis Prescription in the Treatment of 60 Patients of Type 2 Diabetes Mellitus Combined with Carotid Atherosclerosis. *China Med Herald.* 12(25), 107-111.

**4) The control measure did not meet the standard:**

[1] Han, J.H., Zhang, D., Xin, K., and Yang, H.Y. (2020). Effect of Xiaoji Recipe on Stability of Carotid Artery Vulnerable Plaque in Elderly Patients with Type 2 Diabetes Mellitus and Type H Hypertension. *China J Chin Mater Med.* 45(17), 4246-4253. doi: 10.19540/j.cnki.cjcmm.20200302.506. (The dosage of western medicine was different between experimental group and control group.)

[2] Tan, Z.Q., Liu, J.X., and Yu, J. (2021). Efficacy of Insulin Combined with Metformin in the Treatment of Type 2 Diabetes Mellitus Complicated with Carotid Atherosclerosis and Its Effect on Patients' Blood Glucose Levels. *Chin J Clin Ration Drug Use.* 14(10), 28-30. doi: 10.15887/j.cnki.13-1389/r.2021.10.010. (CHM was not used in the intervention.)

[3] Zhao, T.Z., Wang, G.Z., Zheng, J., Zhao, Z.Y., Zhang, W., Yan, Y.L., et al. (2011). The Effect of Naoxintong Combined with Aspirin on Carotid Atherosclerotic Plaque in Diabetic Patients. *Shaanxi Med J.* 40(3), 349-351. (The control group was treated with Chinese patent medicine.)

[4] Jiang, T., Fang, C.H., Guo, C.L., Luo, Y., and Cui, L.Q. (2014). Clinical Observation of Dan-Zhi-Jiang-Tang Capsule Union Lifestyle on Treatment of Type 2 Diabetes Patients with Carotid Plaques. *J Chengdu Univ Tradit Chin Med.* 37(1), 56-59. doi: 10.13593/j.cnki.51-1501/r.2014.01.056. (The experimental group received an intensive lifestyle intervention, while the control group did not.)

[5] Lyu, X.Y. (2012). *Buyang Huanwu Decoction for the Treatment Research of Carotid Atherosclerosis in T2DM*. [Master's thesis]. Jinan: Shandong University of Traditional Chinese Medicine. (The control group was treated with Chinese patent medicine.)

**5) Duplicate literature:**

[1] Du, A.Y., Wang, Z.M., Zuo, W.B., and Zhou, C.L. (2014). Study on the Effect of Regulating Liver and Purging Fire on Carotid Arteriosclerosis in Diabetic Patients. *Inn Mong J Tradit Chin Med.* 33(19), 97-98. doi: 10.16040/j.cnki.cn15-1101.2014.19.024.

[2] Zhai, H.W. (2011). 29 Cases of Diabetic Carotid Arteriosclerosis Plaque Treated by Integrated Traditional Chinese and Western Medicine. *J Emerg Tradit Chin Med.* 20(8), 1304.

[3] Fan, D.L., Xie, Y.L., Wang, L.Y., and Chen, X.C. (2013). Clinical Observation of Zishui Tongmai Decoction in Treatment 30 Cases of Type 2 Diabetes Patients with Carotid Atherosclerosis. *Liaoning J Tradit Chin Med.* 40(8), 1617-1619. doi: 10.13192/j.ljtcm.2013.08.119.fandl.050.

[4] Zuo, W.B., Du, A.Y., Zhou, C.L., and Wang, Z.M. (2013). Effect of Regulating Liver and Purging Fire on Insulin Resistance in Patients with Diabetic Carotid Atherosclerosis. Mod J Integr Tradit Chin West Med. 22(28), 3103-3104

[5] Sun, Q.Y., Du, J., Li, L.H., Jiang, W.H., Xu, L.H., Liu, L.M., et al. (2015). Effect of Tongxinluo Combined with Rosuvastatin on Carotid Plaque Stability in Type 2 Diabetes Mellitus with Carotid Atherosclerosis Patients Chin J Clin Ration Drug Use. 8(34), 99-100. doi: 10.15887/j.cnki.13-1389/r.2015.34.063.

**6) Lack of sufficient data results:**

[1] Chi, C.T. (2013). A Randomized, Double-Blind, Parallel Controlled Clinical Trial of Danzhijiangtang Capsule in the Treatment of Type 2 Diabetes Vascular Lesions with Qi-Yin Deficiency and Blood Stasis Syndrome. [*https://trialsearch.who.int/Trial2.aspx?TrialID=*](https://trialsearch.who.int/Trial2.aspx?TrialID=) *ChiCTR-TRC-13003533.*

**7) Lack of required indicators:**

[1] Wang, Y. (2013). Clinical Observation of Tongxinluo on High Sensitivity C-Reactive Protein and Carotid Atherosclerosis in Type 2 Diabetic Patients. *Chin J Trauma Disab Med.* 21(9), 252-253.

[2] Sun, Q.Y., Du, J., Li, L.H., Jiang, W.H., Xu, L.H., Liu, L.M., et al. (2015). The Effect of Tongxinluo Combined with Rosuvastatin on Serum Cytokines in Patients with Type 2 Diabetes Mellitus Complicated with Carotid Atherosclerosis. *Chin J Clin Ration Drug Use.* 8(31), 35-36. doi: 10.15887/j.cnki.13-1389/r.2015.31.022.

[3] Chen, W.S., Liu, J.S., Zhang, F., and Sang, G.S. (2013). Effect of Yiqi Huoxue Decoction on Carotid Arteriosclerosis Plaque in Diabetic Patients. *J Bengbu Med Coll.* 38(11), 1446-1448. doi: 10.13898/j.cnki.issn.1000-2200.2013.11.009.

[4] Guo, H., and Wang, X.L. (2018). Effects of Puerarin on Oxidative Stress and Hemorheology of Patients with Diabetes Mellitus Combined with Carotid Atherosclerosis. *World Chin Med.* 13(10), 2526-2529.

**8) There were obvious errors:**

[1] Liu, X.L. (2011). The Effect of Naoxintong on Carotid Atherosclerotic Plaque in Diabetic Patients. *Shaanxi J Tradit Chin Med.* 32(8), 973-975. (Inconsistent description of sample size by context)

[2] Pan, Q.Q. (2011). Comparison of Statin and Its Combination with Shexiang Baoxin Pill in the Treatment of Carotid Plaque in Diabetic Patients. *Chin Community Doct.* 13(34), 164-165. (Incorrect choice of statistical method for comparison between multiple groups)

[3] Pan, Y.Y., Zhao, H., Wang, L., and Xing, H. (2016). Effect of Xuezhikang on Carotid Arteries Plaque in Diabetic Patients with Carotid Ultrasound. *Chin J Clin Pharm.* 25(2), 96-98. doi: 10.19577/j.cnki.issn10074406.2016.02.007. (The article was published before the completion of the trial)

[4] Wang, D.J., and Xu, S.F. (2012). Clinical Observation of Jingmaikang Granula on Diabetes Carotid Artherosclerosis Plaques. *Chin J Inf Tradit Chin Med.* 19(7), 10-12. (The article was published before the completion of the trial)

[5] Wang, X.D., Ma, R., Zhao, P., and Bu, Y.M. (2011). Ultrasound Observation of Treating Diabetes Mellitus with Carotid Plaque by Regulating Qi and Resolving Phlegm. *New Chin Med.* 43(12), 40-41. doi: 10.13457/j.cnki.jncm.2011.12.022. (Incorrect choice of random grouping method. Simple random is a sampling method, not a grouping method)

[6] Yin, H. (2019). Clinical Study of Tongxinluo Combined with Atorvastatin in the Treatment of Carotid Atherosclerosis in Diabetic Patients. *Renow Doct.* (6), 246. (Inconsistent description of sample size by context)

[7] Zhan, S.N., Huang, W., and Pan, T.N. (2021). The Effect of Integrated Traditional Chinese and Western Medicine on Type 2 Diabetes Mellitus Complicated with Atherosclerosis. *Shenzhen J Integr Tradit Chin West Med.* 31(7), 35-37. doi: 10.16458/j.cnki.1007-0893.2021.07.016. (Inconsistent description of the data. The range of CIMT in the inclusion criteria is 0.9-1.3 mm, but the mean value of CIMT in the results is around 2 mm)

[8] Zhang, M.G., and Wu, G.L. (2014). The Effect of Yiqi Yangyin Huoxue Tongluo Decoction on Type 2 Diabetes Mellitus Complicated with Carotid Atherosclerotic Plaque. *Clin J Tradit Chin Med.* 26(7), 684-685. doi: 10.16448/j.cjtcm.2014.07.027. (The article was published before the completion of the trial)

[9] Zhao, Y.J., Wang, Y., and Wei, Z. (2018). Effect of Xiaoke Huayu Granules on Carotid Atherosclerosis in Type 2 Diabetes Mellitus. *China Health Care Nutr.* 28(28), 309. doi: 10.3969/j.issn.1004-7484.2018.28.465. (Inconsistent description of sample size by context)

[10] Zhong, J., Ou, Q.L., and Li, Q. (2020). Clinical Observation on 57 Cases of Diabetic Nephropathy Complicated with Carotid Atherosclerosis Treated by Integrated Traditional Chinese and Western Medicine. *Chin J Ethnomed Ethnopharm.* 29(6), 84-87. (Inconsistent description of treatment duration)

**Final Included References:**

[1] Bu, Y.M., Zhao, P., and Ke, W.J. (2018). Clinical Study of Taohong Siwu Tang for Diabetes of Phlegm and Blood Stasis Type with Carotid Atherosclerosis. *New Chin Med.* 50(7), 103-105. doi: 10.13457/j.cnki.jncm.2018.07.029.

[2] Chen, X.Y., Zheng, G.Y., Lin, K., Fu, K.L., and Chen, X., Y. (2017). Effect of Yishen Huoxue Huatan Recipe on Patients with Type 2 Diabetes Mellitus and Carotid Atherosclerosis. *Chin J Integr Tradit West Med.* 37(11), 1301-1304.

[3] Cong, Y.X. (2016). *Clinical Study on Treating Type 2 Diabetic Carotid Atherosclerosis Phlegm Stasis Resistance Pulse Spleen Deficient through Jianpitongmai Decoction*. [Master's thesis]. Jinan: Shandong University of Traditional Chinese Medicine.

[4] Deng, M., Zhang, W.N., and Yang, Y.C. (2012). Clinical Observation of the Self-Developed Qinghua Xiaoyu Decoction Applied to Treatment the Patients with Diabetic Merging Carotid Artery Plaque. *Chin J Diffic Compl Cas.* 11(2), 105-107.

[5] Fan, L.H., Zhang, Z.G., Yuan, L.M., Cao, Z.N., Wang, X.C., Li, N., et al. (2020). Clinical Study of Tongmai Yuban Pill in Treating 35 Patients with Type 2 Diabetes Mellitus Complicated with Carotid Atherosclerosis. *Henan Tradit Chin Med.* 40(10), 1536-1539. doi: 10.16367/j.issn.1003-5028.2020.10.0390.

[6] Fang, C.H., Zhao, J.D., Wang, J.P., Niu, Y.F., Shu, Y.Q., Yuan, A.H., et al. (2013). Effect of Danzhi Jiangtang Capsule Combined with Atorvastatin Calcium on Carotid Intima-Media Thickness in Diabetic Patients without Hypertension. *Moderniz Tradit Chin Med Mater Med-World Sci Technol.* 15(5), 884-890.

[7] Fu, L.L. (2014). *Clinical Study of the Clinical Intervention of Luomaitong to Diabetic Patients with Carotid Atherosclerosis*. [Master's thesis]. Nanjing: Nanjing University of Chinese Medicine.

[8] Guo, Y.X. (2020). *Clinical Observation of Panax Notoginseng Powder in the Treatment of Blood Stasis Syndrome in Type 2 Diabetes Mellitus Complicated with Carotid Atherosclerosis*. [Master's thesis]. Beijing: Beijing University of Chinese Medicine.

[9] Jiao, S.J., Feng, X., and Zhang, N. (2021). Effect of Yuye Decoction on Type 2 Diabetes Mellitus Complicated with Carotid Atherosclerosis. *World Chin Med.* 16(4), 638-642.

[10] Li, L., Jiang, W.H., Li, L.H., Zhai, M.X., Li, H., and Fang, Y.H. (2014). Clinical Study of Tongxinluo Combined with Atorvastatin in the Treatment of Carotid Atherosclerosis in Diabetic Patients. *Chin J Coal Ind Med.* 17(6), 904-906.

[11] Li, Q.Q. (2013). *The Clinical Research of the Changes of Endothelial Function of Blood Vessels in Subclinical AS of T2DM and the Therapeutic Effect of Traditional Chinese Medicine on Carotid IMT*. [Master's thesis]. Jinan: Shandong University of Traditional Chinese Medicine.

[12] Lu, Q.Y., Pan, H., Tang, W.L., Hu, Y.N., Chen, Y.P., and Guo, D.Z. (2016). The Therapeutic Effect of Jiawei Simiao Yongan Decoction on 30 Patients with Type 2 Diabetic Carotid Atherosclerotic Plaque. *Zhejiang J Tradit Chin Med.* 51(7), 500-501. doi: 10.13633/j.cnki.zjtcm.2016.07.018.

[13] Luo, Y. (2015). *To Study the Effect of Yiqi Yangyin Huoxue Prescription on Improving Atherosclerosis in T2DM Macroangiopathy and the Expression of P22phox mRNA and P47phox mRNA in Thoracic Aorta of Rats*. [Master's thesis]. Hefei: Anhui University of Chinese Medicine.

[14] Lyu, S.Q., Zhang, S.F., Su, X.H., Wang, M., Wang, X.Y., and Yu, W.X. (2016). Clinical Observation of Tongxinluo Combined with Atorvastatin in the Treatment of Type 2 Diabetic Carotid Atherosclerosis. *Int Med Health Guid News.* 22(11), 1601-1603.

[15] Mao, X. (2020). Observation on the Curative Effect of Yudan Shengui Capsule Combined with Statins in Treating Diabetes Mellitus with Carotid Atherosclerosis. *Guangming J Chin Med.* 35(5), 739-741.

[16] Shao, X. (2012). *The Research of the Correlation Between Type 2 Diabetes Mellitus Patients with Subclinical AS and Hs-CRP and the Therapeutic Effect of Traditional Chinese Medicine on IMT*. [Master's thesis]. Jinan: Shandong University of Traditional Chinese Medicine.

[17] Shi, L., and Lu, D.F. (2016). Effect of Danzhi Jiangtang Capsule on VEGF and VCAM-1 in Patients with Type 2 Diabetes Carotid Artery Lesion. *Clin J Tradit Chin Med.* 28(6), 807-810. doi: 10.16448/j.cjtcm.2016.0288.

[18] Sun, Q.Y., Du, J., Li, L.H., Jiang, W.H., Xu, L.H., Liu, L.M., et al. (2015a). Clinical Observation of Tongxinluo Combined with Rosuvastatin in the Treatment of Type 2 Diabetes Mellitus Complicated with Carotid Atherosclerosis. *Chin J Clin Ration Drug Use.* 8(28), 56-57. doi: 10.15887/j.cnki.13-1389/r.2015.28.036.

[19] Sun, Q.Y., Du, J., Li, L.H., Jiang, W.H., Xu, L.H., Liu, L.M., et al. (2015b). Effect of Tongxinluo Combined with Rosuvastatin on Carotid Plaque Stability in Type 2 Diabetes Mellitus with Carotid Atherosclerosis Patients *Chin J Clin Ration Drug Use.* 8(34), 99-100. doi: 10.15887/j.cnki.13-1389/r.2015.34.063.

[20] Sun, X.Z., and Fan, G.J. (2011). The Therapeutic Effect of Phlegm-Eliminating and Collateral-Dredging Therapy on Carotid Atherosclerotic Plaque of Patients with Diabetes Mellitus. *New Chin Med.* 43(2), 34-35. doi: 10.13457/j.cnki.jncm.2011.02.033.

[21] Wang, L.Y. (2013). *Zi Water Drink of Arteries and Veins Treatment of Type 2 Diabetes Carotid Atherosclerosis Disease Clinical Research*. [Master's thesis]. Shanghai: Shanghai University of Traditional Chinese Medicine.

[22] Wang, Z.Q., Lyu, S.Q., Wang, H.L., Qiao, K.M., Wang, L.X., Su, X.H., et al. (2021). A Study on the Effectiveness of the Wenyang Huaqi Therapy on Diabetic Carotid Atherosclerosis Plaque. *Clin J Chin Med.* 13(11), 67-70.

[23] Xu, Q.H. (2016). *Clinical Observation of Jianpi Xiaozhi Formula Intervenes Carotid Artery Intima Media Thickness and MMP-9 Levels in the Type 2 Diabdetes Mellitus Complicated with Atherosclersis Patients*. [Master's thesis]. Hefei: Anhui University of Chinese Medicine.

[24] Yang, M. (2017). *Clinical Observation of Jiawei Zicui Tongmai Decoction for the Treatment of Type 2 Diabetes with Carotid Atherosclerosis*. [Master's thesis]. Changsha: Hunan University of Chinese Medicine.

[25] Yang, Q.F. (2013). *The Study on the Influence of the Method of Tonifying Kidney Supplementing Qi and Activating Blood Circulation on Elderly Type 2 Diabetic Carotid Atherosclerosis and Endothelial Function*. [Dissertation]. Jinan: Shandong University of Traditional Chinese Medicine.

[26] Yang, X.Y., Rui, F., and Zhang, X.Y. (2016). Effect of Hawthorn Granule Combined with Simvastatin on CIMT in Diabetic Patients. *Zhejiang Clin Med J.* 18(8), 1449-1450.

[27] Zhou, D.Q., Li, X.M., Yang, J., and Guo, S.P. (2011). Clinical Observation of Xiaoke Huayu Capsule in the Treatment of Type 2 Diabetic Carotid Artery Disease. *China Prac Med.* 6(11), 163-165. doi: 10.14163/j.cnki.11-5547/r.2011.11.018.

[28] Zuo, W.B., Du, A.Y., Zhou, C.L., and Wang, Z.M. (2013a). Clinical Study of Regulating Liver and Purging Fire Method in Treating Type 2 Diabetes Mellitus with Carotid Atherosclerosis. *New Chin Med.* 45(8), 125-127. doi: 10.13457/j.cnki.jncm.2013.08.057.

[29] Zuo, W.B., Du, A.Y., Zhou, C.L., and Wang, Z.M. (2013b). Effect of Regulating Liver and Purging Fire on Insulin Resistance in Patients with Diabetic Carotid Atherosclerosis. *Mod J Integr Tradit Chin West Med.* 22(28), 3103-3104.

# Supplementary Material S4. Meta-regression of CIMT, TC, TG, LDL-C, HDL-C, FBG, 2hPG and HbA1c.

4.1 Summary of meta-regression results.

| **Variable** | **Number of studies** | **Coefficient** | **95% CI** | **P-value** | **Tau^2^** | **AdjR^2^** |
| --- | --- | --- | --- | --- | --- | --- |
| CIMT | 25 |  |  |  | 0.010 |  |
| Average age | 25 | 0.0032 | [-0.0019, 0.0082] | 0.206 | 0.010 | 3.35% |
| Treatment duration | 25 | 0.0030 | [-0.0093, 0.0153] | 0.618 | 0.011 | -3.84% |
| Dosage form | 25 | 0.0962 | [0.0117, 0.1806] | 0.027 | 0.009 | 20.32% |
| TC | 21 |  |  |  | 0.190 |  |
| Average age | 21 | 0.0012 | [-0.0234, 0.0257] | 0.922 | 0.196 | -5.70% |
| Treatment duration | 21 | 0.0314 | [-0.0351, 0.0962] | 0.342 | 0.186 | -0.26% |
| Dosage form | 21 | 0.1532 | [-0.2775, 0.5838] | 0.466 | 0.190 | -2.42% |
| TG | 21 |  |  |  | 0.050 |  |
| Average age | 21 | 0.0164 | [0.0052, 0.0276] | 0.006 | 0.028 | 41.53% |
| Treatment duration | 21 | 0.0197 | [-0.0165, 0.0558] | 0.269 | 0.047 | 2.52% |
| Dosage form | 21 | 0.2113 | [-0.0022, 0.4248] | 0.052 | 0.039 | 20.13% |
| LDL-C | 21 |  |  |  | 0.050 |  |
| Average age | 21 | 0.0008 | [-0.0139, 0.0156] | 0.910 | 0.051 | -5.59% |
| Treatment duration | 21 | 0.0258 | [-0.0052, 0.0568] | 0.098 | 0.043 | 9.76% |
| Dosage form | 21 | 0.0085 | [-0.2259, 0.2428] | 0.940 | 0.051 | -5.95% |
| HDL-C | 17 |  |  |  | 0.030 |  |
| Average age | 17 | -0.0104 | [-0.0217, 0.0008] | 0.067 | 0.026 | 17.50% |
| Treatment duration | 17 | -0.0175 | [-0.0464, 0.0115] | 0.217 | 0.030 | 4.43% |
| Dosage form | 17 | -0.1064 | [-0.3028, 0.0901] | 0.266 | 0.031 | 1.89% |
| FBG | 22 |  |  |  | 0.110 |  |
| Average age | 22 | 0.0048 | [-0.0162, 0.0259] | 0.638 | 0.122 | -6.15% |
| Treatment duration | 22 | 0.0003 | [-0.0536, 0.0541] | 0.992 | 0.123 | -7.23% |
| Dosage form | 22 | -0.0890 | [-0.4642, 0.2862] | 0.626 | 0.121 | -5.76% |
| 2hPG | 15 |  |  |  | 0.590 |  |
| Average age | 15 | 0.0321 | [-0.0329, 0.0971] | 0.306 | 0.596 | 0.54% |
| Treatment duration | 15 | 0.0391 | [-0.1026, 0.1808] | 0.561 | 0.623 | -4.08% |
| Dosage form | 15 | -0.4916 | [-1.4252, 0.4420] | 0.276 | 0.564 | 5.87% |
| HbA1c | 20 |  |  |  | 0.090 |  |
| Average age | 20 | 0.0077 | [-0.0149, 0.0303] | 0.484 | 0.124 | -3.73% |
| Treatment duration | 20 | 0.0060 | [-0.0540, 0.0661] | 0.835 | 0.132 | -10.52% |
| Dosage form | 20 | -0.1666 | [-0.5706, 0.2375] | 0.398 | 0.127 | -5.73% |

4.2 Meta-regression analysis of CIMT.


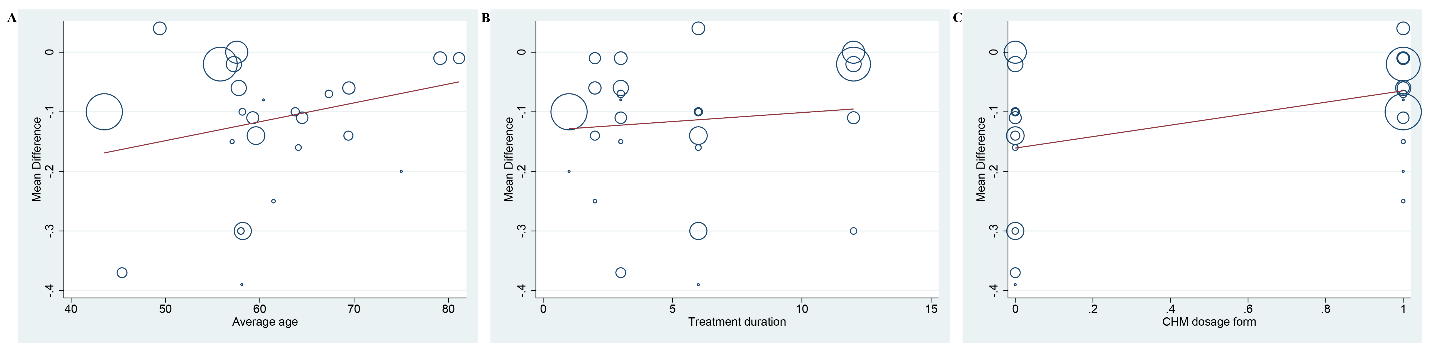


Meta-regression analysis of CIMT on (a) Average age, (b) Treatment duration and (c) Dosage form.

4.3 Subgroup analysis of different dosage forms for CIMT.


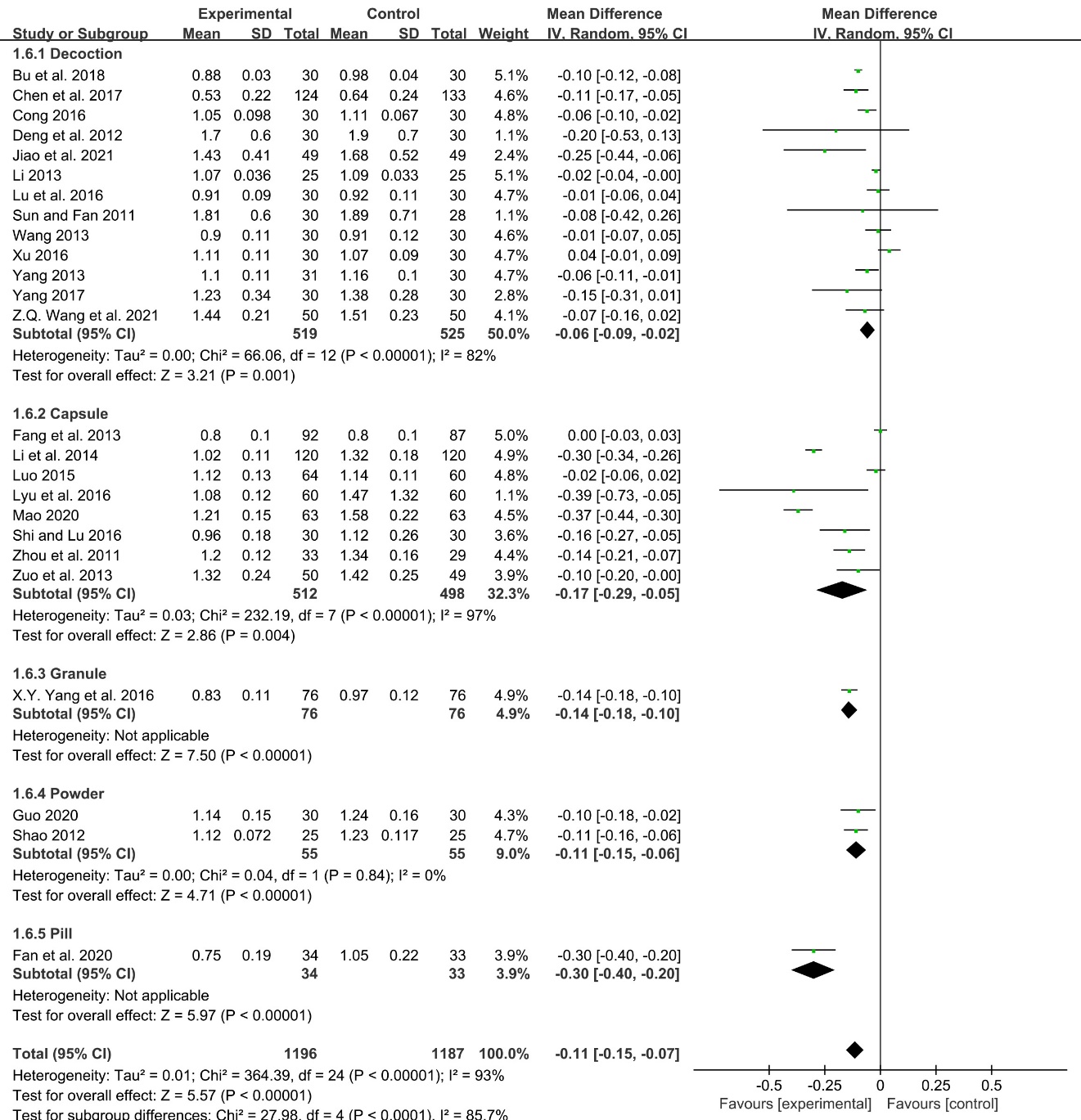


4.4 Meta-regression analysis of TC.


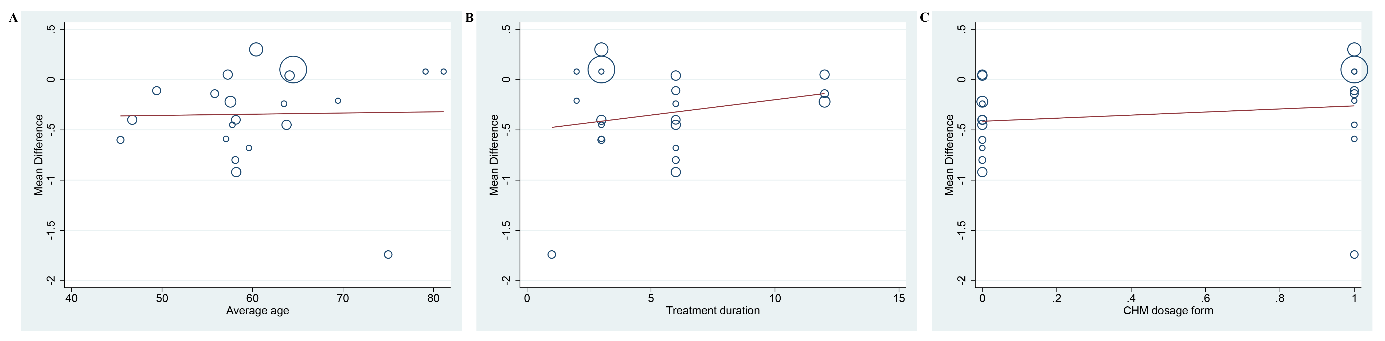
Meta-regression analysis of TC on (a) Average age, (b) Treatment duration and (c) Dosage form.

4.5 Meta-regression analysis of TG.


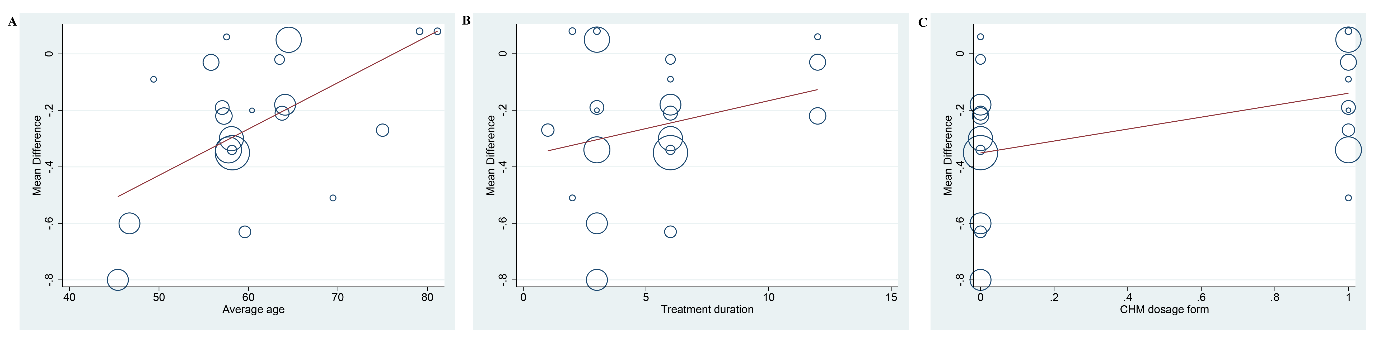


Meta-regression analysis of TG on (a) Average age, (b) Treatment duration and (c) Dosage form.

4.6 Meta-regression analysis of LDL-C.
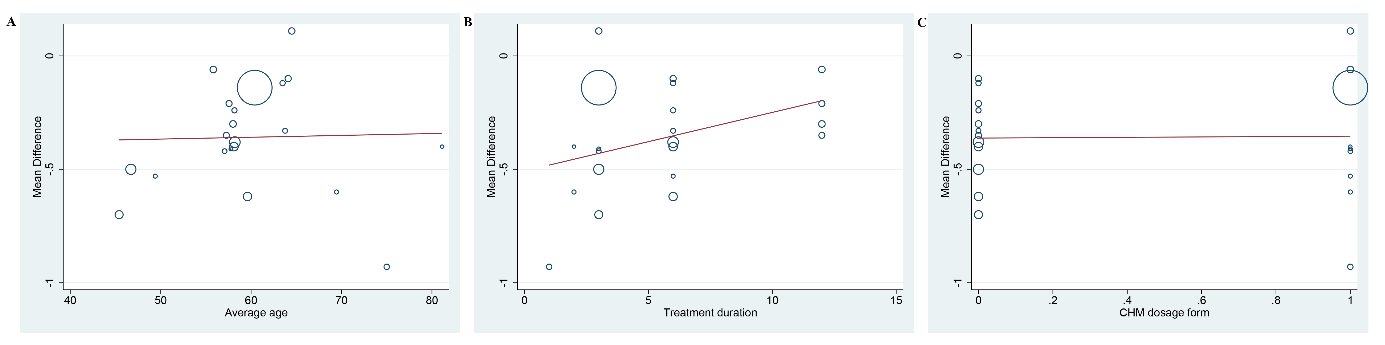
 Meta-regression analysis of LDL-C on (a) Average age, (b) Treatment duration and (c) Dosage form.

4.7 Meta-regression analysis of HDL-C.


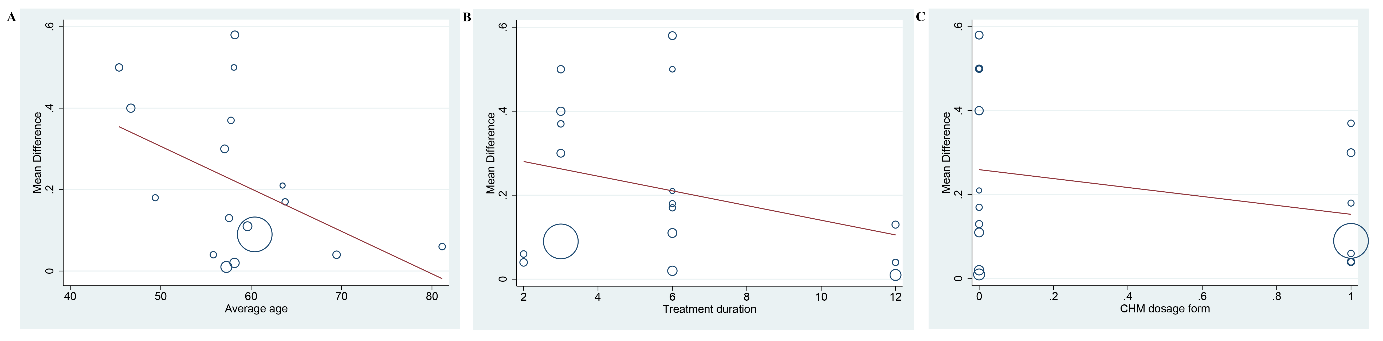


Meta-regression analysis of HDL-C on (a) Average age, (b) Treatment duration and (c) Dosage form.

4.8 Meta-regression analysis of FBG.


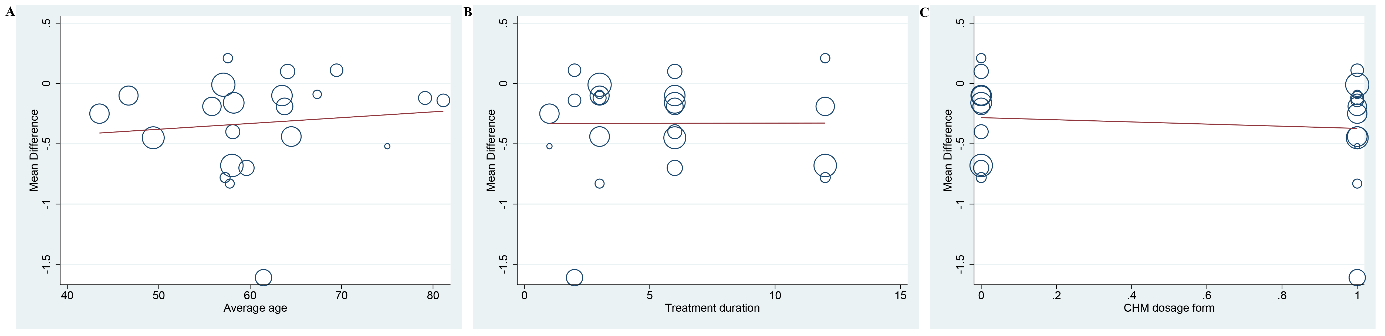


Meta-regression analysis of FBG on (a) Average age, (b) Treatment duration and (c) Dosage form.

4.9 Meta-regression analysis of 2hPG.


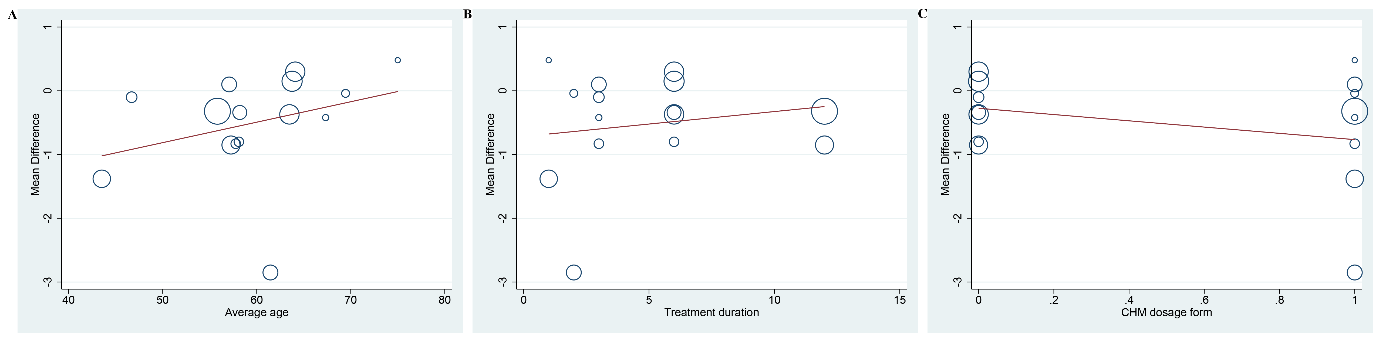


Meta-regression analysis of 2hPG on (a) Average age, (b) Treatment duration and (c) Dosage form.

4.10 Meta-regression analysis of HbA1c.


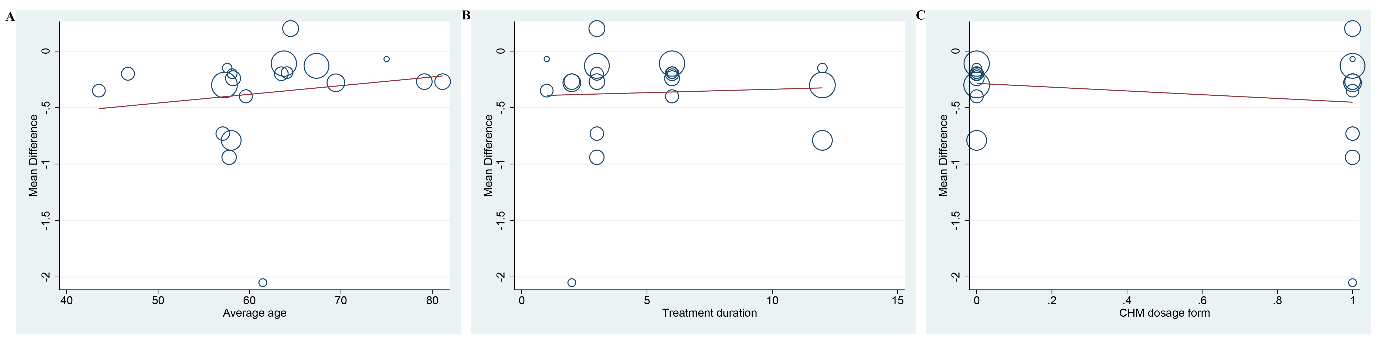


Meta-regression analysis of HbA1c on (a) Average age, (b) Treatment duration and (c) Dosage form.

# Supplementary Material S5. Sensitivity analysis.

5.1 The results of sensitivity analysis of CIMT.

5.2 The results of sensitivity analysis of Crouse score.

5.3 The results of sensitivity analysis of TC.

5.4 The results of sensitivity analysis of TG.

5.5 The results of sensitivity analysis of LDL-C.

5.6 The results of sensitivity analysis of HDL-C.

5.7 The results of sensitivity analysis of FBG.

5.8 The results of sensitivity analysis of 2hPG.

5.9 The results of sensitivity analysis of HbA1c.

5.10 The results of sensitivity analysis of NO.

5.11 The results of sensitivity analysis of ET-1.

5.12 The results of sensitivity analysis of HOMA-IR.

# Supplementary material S6. Subgroup analysis of Crouse score and HOMA-IR.

6.1 Summary of subgroup analysis results.

| **Subgroups** | **Number of**  **studies** | **Result (95% CI)** | **P-value for overall effect** | **I^2^(%)** | **P-value for subgroup difference** |
| --- | --- | --- | --- | --- | --- |
| Crouse score |  |  |  |  |  |
| All studies | 4 | MD: -1.21 [-1.35, -1.07] | ＜0.001 | 0 |  |
| Average age |  |  |  |  |  |
| ≤ 60 years old | 2 | MD: -1.20 [-1.34, -1.06] | ＜0.001 | 55 | 0.48 |
| ＞60 years old | 2 | MD: -1.61 [-2.71, -0.50] | 0.004 | 0 |  |
| Treatment duration |  |  |  |  |  |
| ＜6months | 3 | MD: -1.37 [-1.62, -1.13] | ＜0.001 | 0 | 0.11 |
| ≥ 6months | 1 | MD: -1.13 [-1.30, -0.96] | ＜0.001 | - |  |
| Dosage form |  |  |  |  |  |
| Decoction | 2 | MD: -1.61 [-2.71, -0.50] | 0.004 | 0 | 0.48 |
| Other dosage form | 2 | MD: -1.20 [-1.34, -1.06] | ＜0.001 | 55 |  |
| HOMA-IR |  |  |  |  |  |
| All studies | 6 | SMD: -0.88 [-1.36, -0.41] | ＜0.001 | 90 |  |
| Average age |  |  |  |  |  |
| ≤ 60 years old | 5 | SMD: -0.93 [-1.47, -0.39] | ＜0.001 | 92 | 0.51 |
| ＞60 years old | 1 | SMD: -0.62 [-1.19, -0.05] | 0.030 | - |  |
| Treatment duration |  |  |  |  |  |
| ＜6months | 1 | SMD: -0.98 [-1.27, -0.69] | ＜0.001 | - | 0.55 |
| ≥ 6months | 5 | SMD: -0.86 [-1.47, -0.25] | 0.006 | 92 |  |
| Dosage form |  |  |  |  |  |
| Decoction | 1 | SMD: -0.52 [-1.03, -0.00] | 0.050 | - | 0.31 |
| Other dosage form | 5 | SMD: -0.95 [-1.49, -0.41] | ＜0.001 | 92 |  |

6.2 Subgroup analysis of Crouse score.


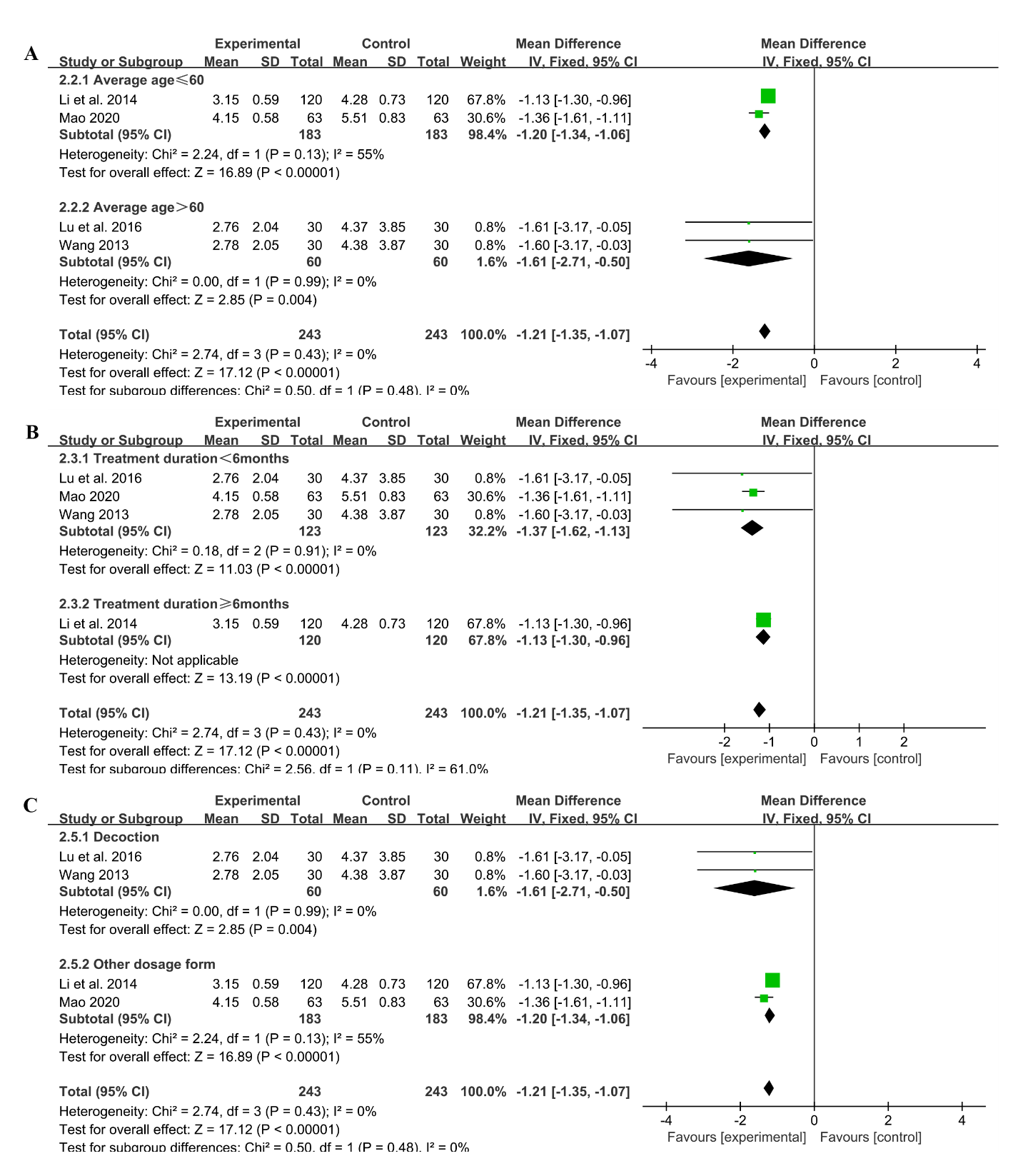


Subgroup analysis of Crouse score on (a) Average age, (b) Treatment duration and (c) Dosage form.

6.3 Subgroup analysis of HOMA-IR.


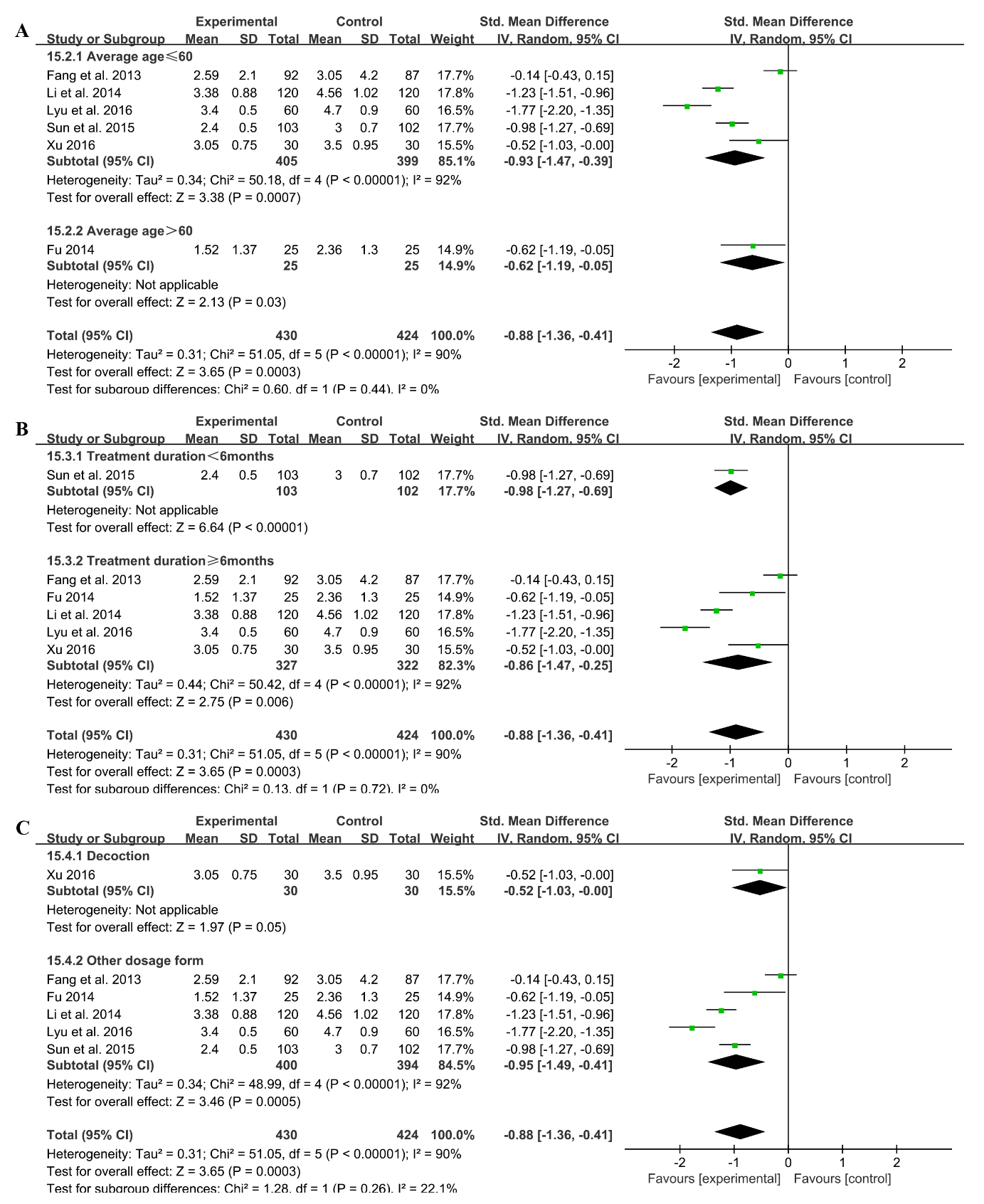


Subgroup analysis of HOMA-IR on (a) Average age, (b) Treatment duration and (c) Dosage form.

# Supplementary material S7. Publication Bias

7.1 The results of publication bias of TC.

7.2 The results of publication bias of LDL-C.

7.3 The results of publication bias of HDL-C.

7.4 The results of publication bias of CIMT.

7.5 The results of publication bias of TG.

7.6 The results of publication bias of FBG.

7.7 The results of publication bias of 2hPG.

7.8 The results of publication bias of HbA1c.

# Supplementary Material S8. Assessment of evidence quality for each outcome

| **Quality assessment** | | | | | | | **No of patients** | | **Effect** | | **Quality** | **Importance** |  |
| --- | --- | --- | --- | --- | --- | --- | --- | --- | --- | --- | --- | --- | --- |
|  |  |  |  |  |  |  |  |  |  |  |  |  |  |
| **No of studies** | **Design** | **Risk of bias** | **Inconsistency** | **Indirectness** | **Imprecision** | **Other considerations** | **Treatment** | **Control** | **Relative (95% CI)** | **Absolute** |  |  |  |
| **CIMT (Better indicated by lower values)** | | | | | | | | | | | | |  |
| 26 | randomised trials | serious^1^ | serious^2^ | no serious indirectness | no serious imprecision | none | 1299 | 1289 | - | MD 0.12 lower (0.16 to 0.08 lower) | ⊕⊕OO LOW | CRITICAL |  |
| **Crouse score (Better indicated by lower values)** | | | | | | | | | | | | |  |
| 5 | randomised trials | serious^1^ | no serious inconsistency | no serious indirectness | no serious imprecision | none | 346 | 345 | - | MD 1.23 lower (1.35 to 1.12 lower) | ⊕⊕⊕O MODERATE | CRITICAL |  |
| **TC (Better indicated by lower values)** | | | | | | | | | | | | |  |
| 21 | randomised trials | serious^1^ | serious^2^ | no serious indirectness | no serious imprecision | reporting bias^4^ | 1103 | 1098 | - | MD 0.34 lower (0.54 to 0.14 lower) | ⊕OOO VERY LOW | CRITICAL |  |
| **TG (Better indicated by lower values)** | | | | | | | | | | | | |  |
| 21 | randomised trials | serious^1^ | serious^2^ | no serious indirectness | no serious imprecision | none | 1103 | 1098 | - | MD 0.26 lower (0.37 to 0.15 lower) | ⊕⊕OO LOW | CRITICAL |  |
| **LDL-C (Better indicated by lower values)** | | | | | | | | | | | | |  |
| 21 | randomised trials | serious^1^ | serious^2^ | no serious indirectness | no serious imprecision | reporting bias^4^ | 1107 | 1101 | - | MD 0.36 lower (0.47 to 0.25 lower) | ⊕OOO VERY LOW | CRITICAL |  |
| **HDL-C (Better indicated by higher values)** | | | | | | | | | | | | |  |
| 17 | randomised trials | serious^1^ | serious^2^ | no serious indirectness | no serious imprecision | reporting bias^4^ | 889 | 875 | - | MD 0.22 higher (0.13 to 0.3 higher) | ⊕OOO VERY LOW | CRITICAL |  |
| **FBG (Better indicated by lower values)** | | | | | | | | | | | | |  |
| 22 | randomised trials | serious^1^ | serious^2^ | no serious indirectness | no serious imprecision | none | 1123 | 1120 | - | MD 0.33 lower (0.5 to 0.16 lower) | ⊕⊕OO LOW | IMPORTANT |  |
| **2hPG (Better indicated by lower values)** | | | | | | | | | | | | |  |
| 15 | randomised trials | serious^1^ | serious^2^ | no serious indirectness | no serious imprecision | none | 707 | 701 | - | MD 0.52 lower (0.95 to 0.09 lower) | ⊕⊕OO LOW | IMPORTANT |  |
| **HbA1c (Better indicated by lower values)** | | | | | | | | | | | | |  |
| 20 | randomised trials | serious^1^ | serious^2^ | no serious indirectness | no serious imprecision | none | 1068 | 1065 | - | MD 0.36 lower (0.51 to 0.21 lower) | ⊕⊕OO LOW | IMPORTANT |  |
| **NO (Better indicated by higher values)** | | | | | | | | | | | | |  |
| 4 | randomised trials | serious^1^ | serious^2^ | no serious indirectness | serious^3^ | none | 153 | 144 | - | MD 6.84 higher (1.16 to 12.53 higher) | ⊕OOO VERY LOW | IMPORTANT |  |
| **ET-1 (Better indicated by lower values)** | | | | | | | | | | | | |  |
| 4 | randomised trials | serious^1^ | no serious inconsistency | no serious indirectness | no serious imprecision | none | 153 | 144 | - | MD 4.44 lower (5.8 to 3.08 lower) | ⊕⊕⊕O MODERATE | IMPORTANT |  |
| **HOMA-IR (Better indicated by lower values)** | | | | | | | | | | | | |  |
| 7 | randomised trials | serious^1^ | serious^2^ | no serious indirectness | no serious imprecision | none | 480 | 473 | - | SMD 0.83 lower (1.25 to 0.42 lower) | ⊕⊕OO LOW | IMPORTANT |  |
| **HOMA-β (Better indicated by higher values)** | | | | | | | | | | | | |  |
| 2 | randomised trials | serious^1^ | no serious inconsistency | no serious indirectness | no serious imprecision | none | 153 | 151 | - | SMD 0.64 higher (0.41 to 0.87 higher) | ⊕⊕⊕O MODERATE | IMPORTANT |  |

^1^ Poor methodological quality, such as not using blinding or not reporting in detail the specific methods for generating random sequences and allocation concealment.
^2^ Large differences in the effect size of each study point or small overlap of confidence intervals, or large heterogeneity.

^3^ Small sample size or wide confidence interval.

^4^ The funnel plot showed asymmetric distribution and Egger's test indicated possible publication bias.
